# Synthesis and Biological Evaluation of Bicyclo[1.1.1]pentane-Containing Aromatic Lipoxin A_4_ Analogues

**DOI:** 10.1021/acs.orglett.2c02345

**Published:** 2022-08-08

**Authors:** Benjamin Owen, Monica de Gaetano, Andrew Gaffney, Catherine Godson, Patrick J. Guiry

**Affiliations:** † Centre for Synthesis and Chemical Biology, School of Chemistry, University College Dublin, Belfield, Dublin 4, Ireland; ‡ School of Biology & Environmental Science, Diabetes Complications Research Centre, UCD Conway Institute, University College Dublin, Belfield, Dublin 4, Ireland; § School of Medicine, Diabetes Complications Research Centre, UCD Conway Institute, University College Dublin, Belfield, Dublin 4, Ireland

## Abstract

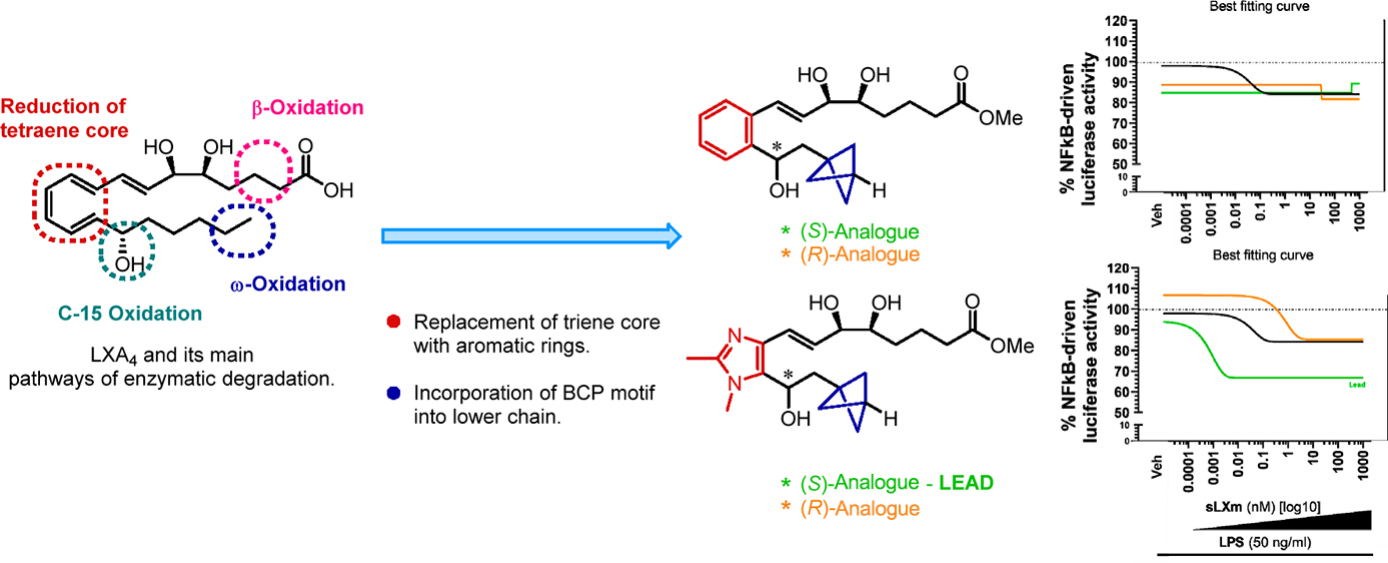

Lipoxins are important drivers of inflammation resolution, suggesting a potential therapeutic benefit. Bicyclo[1.1.1]pentanes (BCPs) are potential isosteric replacements for arenes and/or alkyl groups within drug candidates. We carried out an asymmetric synthesis of four BCP-containing synthetic lipoxin A_4_ mimetics (BCP-sLXms) in which the key steps were a Suzuki coupling, an asymmetric ketone reduction, and a triethylborane-initiated radical bicyclopentylation. These mimetics were screened for their impact on inflammatory responses, and one imidazolo-BCP-sLXm (**6a**) was found to possess high anti-inflammatory activity.

Inflammation is a critical response to infection and injury, and it is vital that the amplitude and duration of the inflammatory response be controlled in space and time. The precise regulation of the onset, duration, and resolution of inflammation reflects responses to distinct signaling molecules produced at specific times.^1^ Dysregulation of these processes underpins the pathology of numerous prevalent diseases.^2^ Lipoxins (LXs) make up a class of endogenously generated eicosanoids typically generated through transcellular metabolism at a site of inflammation.^3^ The generation of lipoxins marks the initiation of the resolution phase of inflammation. Additional lipid mediators have been identified, which promote the resolution of inflammation, and these are collectively described as specialized pro-resolving mediators (SPMs), which typically act on specific G protein-coupled receptors, including FPR2.^4^ LXA_4_ (**1**) and its aspirin-triggered C-15 epimer, AT-LXA_4_, have been shown to activate the FPR2 receptor and inhibit the recruitment of polymorphonuclear neutrophils (PMNs) to the site of inflammation, while promoting recruitment of monocytes and stimulating the nonphlogistic phagocytosis (efferocytosis) of apoptotic PMNs.^5^

Although their potent anti-inflammatory properties have been well documented, their chemical and metabolic instability decreases the therapeutic exploitation of these actions. Metabolic instability is characterized by oxidation of the alcohol at C-15, reduction of the double bond between C-13 and C-14, and ω-oxidation at C-20 by P450 enzymes (Figure 1). There has been much interest in designing stable synthetic lipoxin mimetics,^6^ and we have previously described the synthesis of LXA_4_ mimetic **2** in which the triene of native LXA_4_ was replaced by a benzene ring.^7^ Since then, we have also described the synthesis and biological evaluation of a number of heteroaromatic LXA_4_ analogues containing different five- and six-membered heterocycles in place of the triene core. These have included pyridine, oxazole, imidazole (**3**), and quinoxaline (**4**) analogues that have shown favorable anti-inflammatory properties comparable or superior to those of native LXA_4_ (Figure 1).^8−10^ As part of our ongoing structure–activity relationship (SAR) studies, we sought to explore the effect of incorporating a bicyclo[1.1.1]pentane (BCP) moiety into the lower C-16–C-20 alkyl chain of our (hetero)aromatic LXA_4_ mimetics. In recent years, there has been significant interest in BCPs as sp^3^-rich surrogates within potentially bioactive molecules for *para*-substituted arenes as well as for *tert*-butyl groups and alkynes.^11−14^ We wanted to determine whether a BCP ring could also serve as a more rigid and metabolically resistant bioisostere for alkyl chains in fatty acid-derived molecules. Previous studies have shown that the incorporation of a phenoxy or *p*-fluorophenoxy substituent into the lower alkyl chain of LXA_4_ as a way of blocking ω-oxidation has beneficial effects on the compound’s metabolic stability and anti-inflammatory properties.^15^ A more recent study by Ishimura also demonstrated the potential benefits of incorporating small aliphatic rings into fatty acid derivatives as a way of increasing conformational rigidity.^16^ With this in mind, we selected four target BCP-containing analogues to be synthesized. These were benzo analogue **5a** and imidazolo analogue **6a**, as well as their C-15 epimers **5b** and **6b**, respectively, which were chosen so that their anti-inflammatory properties could be readily compared with those of native LXA_4_ as well as the current lead compound of our ongoing SAR studies, imidazole **3** and quinoxaline **4**. Our retrosynthetic analysis (Scheme 1) proposes that all of the analogues could be synthesized via a Suzuki coupling between boronic ester “upper chain” **10** and a BCP-containing “lower chain” (**9** or **11**), followed by a stereoselective ketone reduction and acetonide deprotection.

**Figure 1:**
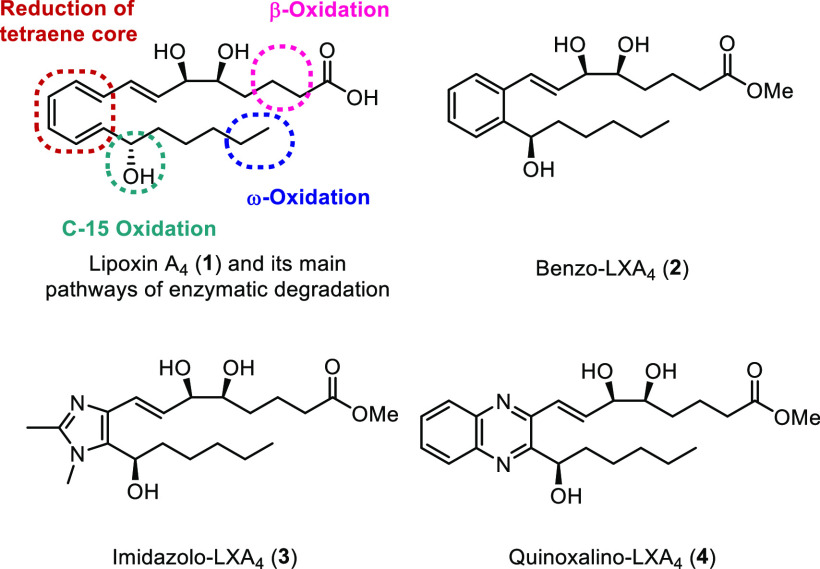
Lipoxin A_4_ (**1**) and examples of aromatic synthetic LXA_4_ mimetics (**2–4**).

**Scheme 1:**
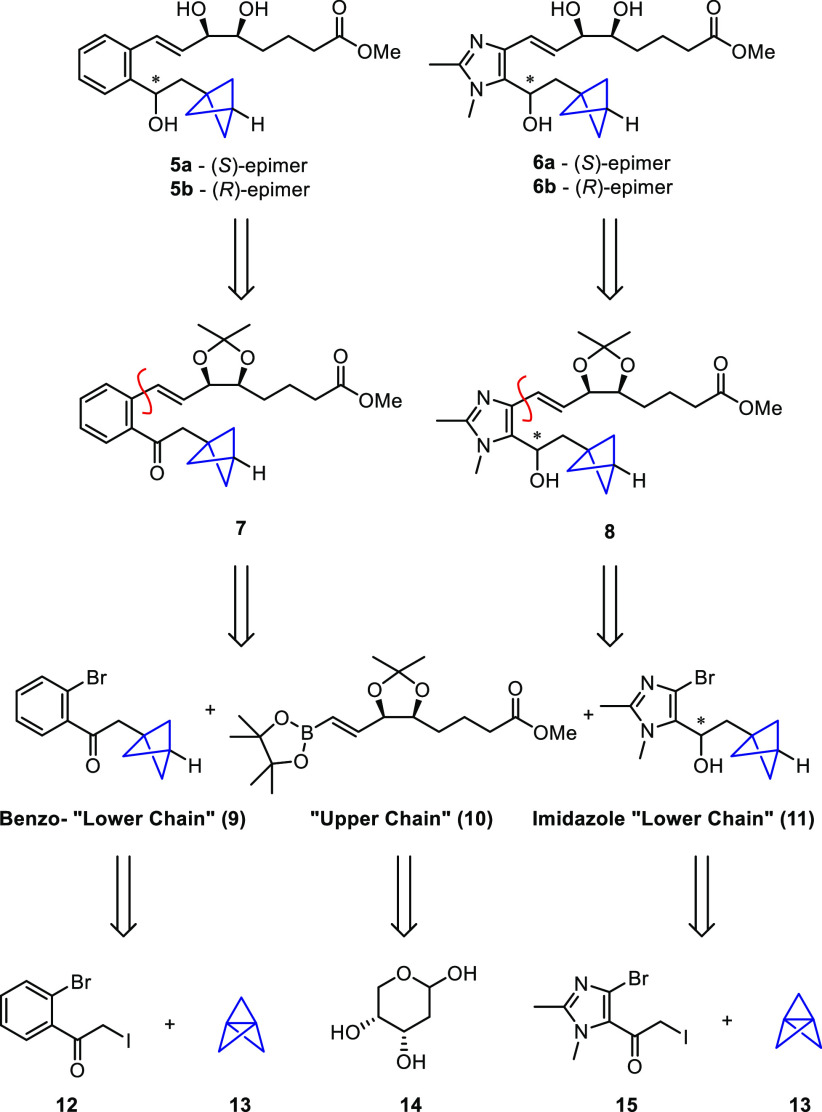
Retrosynthetic Analysis of Target BCP-Containing Aromatic LXA_4_ Mimetics **5** and **6**

Inspired by recent work reported by Anderson,^17−19^ we believed the key BCP moiety could be readily installed via a triethylborane-initiated atom transfer radical addition (ATRA) reaction between α-iodoketone **12** or **15** and [1.1.1]propellane (**13**).

The synthesis of boronic ester **10** was recently reported by us for the synthesis of our quinoxaline LXA_4_ analogues.^10^ The modular nature of our retrosynthetic strategy means that the same coupling partner can easily be used in a number of different Suzuki reactions to produce a wide array of different heteroaromatic LXA_4_ mimetics, including the target BCP-containing analogues. With boronic ester **10** in hand, we turned our attention to the synthesis of the BCP-containing lower chain unit **9** (Scheme 2). Iodoketone **12** was prepared from 2′-bromoacetophenone (**16**) via an α-bromination/Finkelstein sequence and then used as a substrate for the radical bicyclopentylation. Pleasingly, upon reaction with a solution of [1.1.1]propellane (**13**) in the presence of substoichiometric BEt_3,_ complete conversion to iodo-BCP **17** was observed. However, the subsequent deiodination reaction, which was carried out immediately after the bicyclopentylation, proved to be somewhat problematic. To our surprise, tributyltin hydride in the presence of BEt_3_ resulted in no reaction, whereas switching the hydrogen atom source to tris(trimethylsilyl)silane (TTMSS) resulted in the successful formation of the desired ketone **9**, albeit alongside a complex mixture of side products. Following column chromatography, ketone **9** was obtained as an inseparable mixture with debrominated product **18** in an approximately 2.5:1 ratio as determined by ^1^H NMR spectroscopic analysis. On the basis of this ratio, the yield of **9** was calculated to be approximately 28% over two steps. Despite the somewhat disappointing yield, the mixture of **9** and **18** was relatively easy to obtain and could be carried forward to the subsequent Suzuki coupling without any further attempts at purification. The desired coupled product **7** was obtained in 62% yield following a microwave-assisted reaction with **10** in the presence of Pd(PPh_3_)_4_ and aqueous K_2_CO_3_, after which the unreactive impurity **18** was readily removed via column chromatography. From this common intermediate, both C-15 epimers, **19a** and **19b**, were selectively formed via an asymmetric reduction of the ketone using either enantiomer of DIP chloride. (−)-DIP chloride afforded *S*-epimer **19a** in 71% yield and a de of 98%, and (+)-DIP-chloride afforded *R*-epimer **19b** in 51% yield and a de of 96%. Finally, the acetonide deprotection of both compounds was successfully carried out using camphorsulfonic acid (CSA), and target BCP-containing LXA_4_ analogues **5a** and **5b** were obtained in 45% and 24% yields, respectively.

**Scheme 2:**
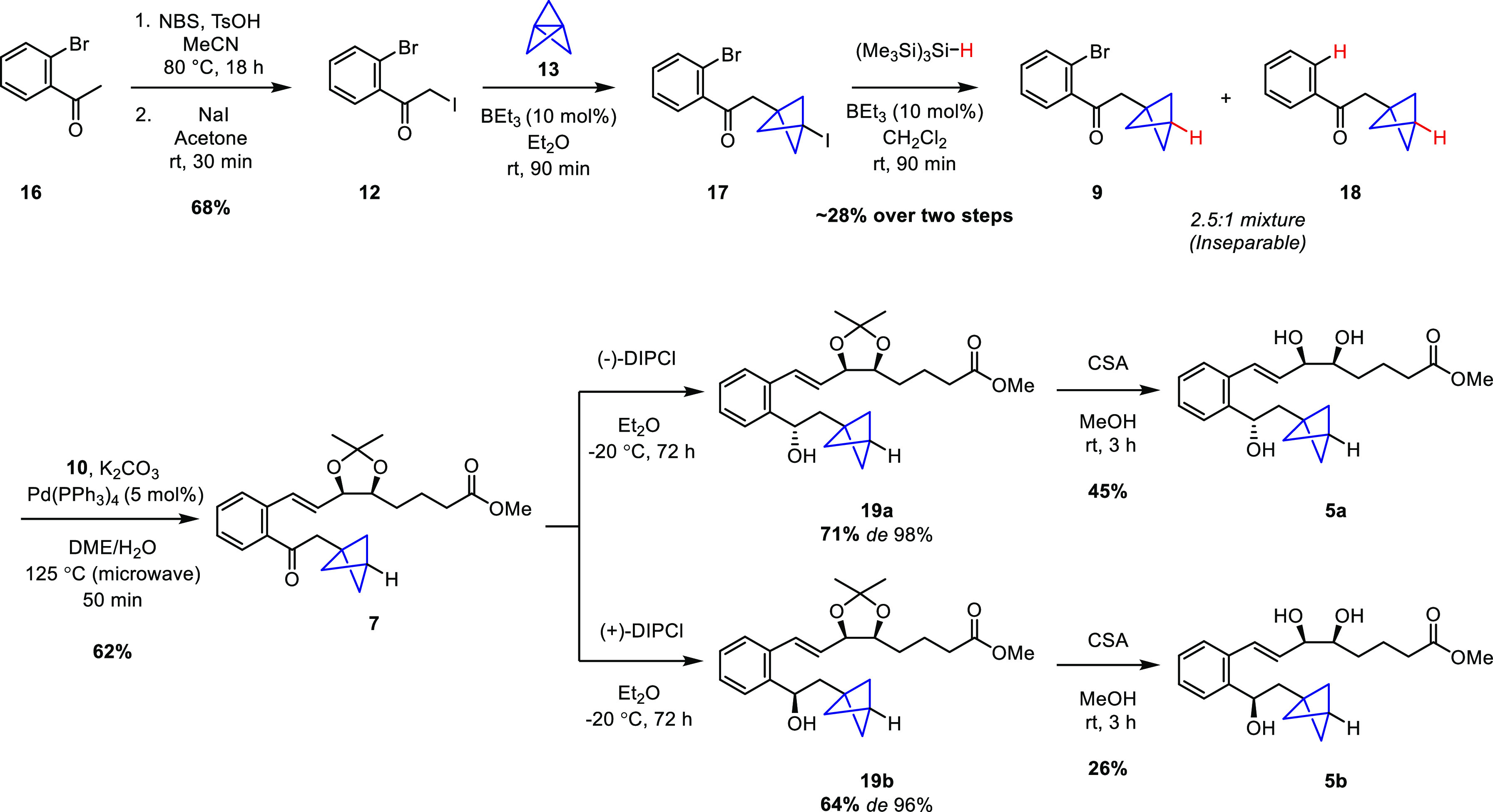
Asymmetric Synthesis of BCP-Containing Benzo-LXA_4_ Mimetics **5a** and **5b**

A very similar approach was used to synthesize the imidazole lower chain **11** (Scheme 3). 1,2-Dimethylimidazole (**20**) was first dibrominated with *N*-bromosuccinimide and then selectively acetylated using *n*-butyllithium and acetyl chloride to form methyl ketone **22**. Once again, an α-bromination/Finkelstein sequence converted **22** to iodoketone **15**, and a BEt_3_-initiated ATRA reaction with **13** followed by a TTMSS-mediated deiodination led to the formation of the desired BCP-containing ketone **24**, which was successfully isolated in a somewhat low yield of 34% over two steps. On the basis of the synthesis of our previously reported imidazolo-LXA_4_ mimetics,^9^ we decided to carry out the asymmetric reduction of **24** before the Suzuki coupling by carrying out an asymmetric hydrogenation in the presence of Noyori’s catalyst, RuCl_2_[DM-BINAP][DAIPEN], under 20 bar of hydrogen gas. By using either enantiomer of the ruthenium catalyst, both enantiomers of **11** could be obtained in high enantiomeric excess following recrystallization from chloroform by vapor diffusion of pentane. The (*R*,*R*)-Ru catalyst afforded (*S*)-**11** in 98% ee, while the (*S*,*S*)-Ru catalyst afforded (*R*)-**11** in 99% ee. In each case, the absolute configuration was confirmed by X-ray crystallography.

**Scheme 3:**
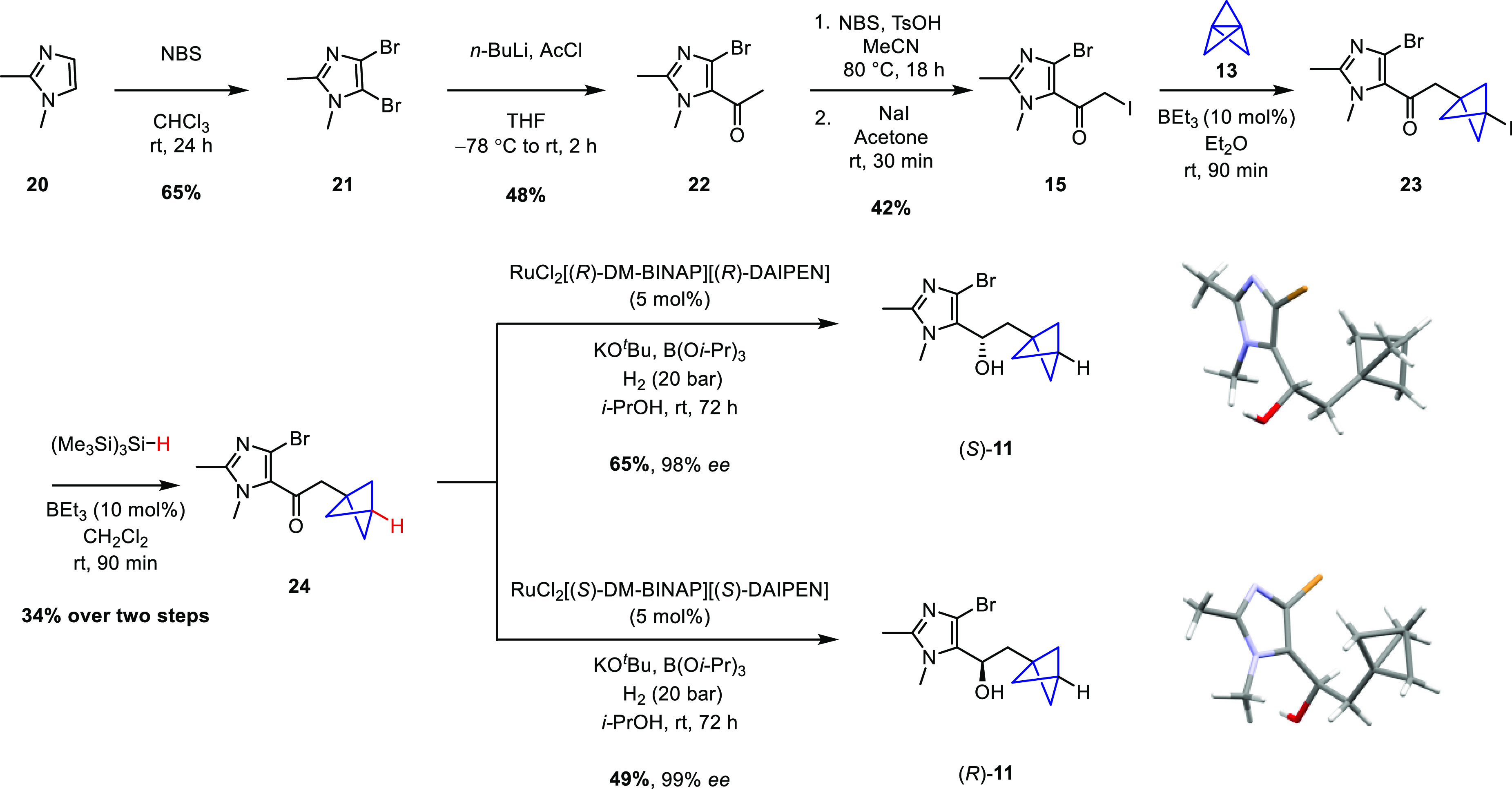
Asymmetric Synthesis of BCP-Containing Imidazole Coupling Partners (*S*)-**11** and (*R*)-**11**

The Suzuki coupling between **10** and **11** was initially attempted using the same microwave-assisted conditions that were used to form **7**, but no reaction was observed. However, after changing the catalyst to PdCl_2_(dppf) and refluxing the reaction mixture in toluene for 18 h, we obtained the desired coupled products **8a** and **8b** in 73% and 66% yields, respectively. Finally, the acetonide deprotection of both compounds was successfully carried out using ZrCl_4_, and two more target BCP-containing LXA_4_ analogues, **6a** and **6b**, were isolated in 71% and 77% yields, respectively (Scheme 4).

**Scheme 4:**
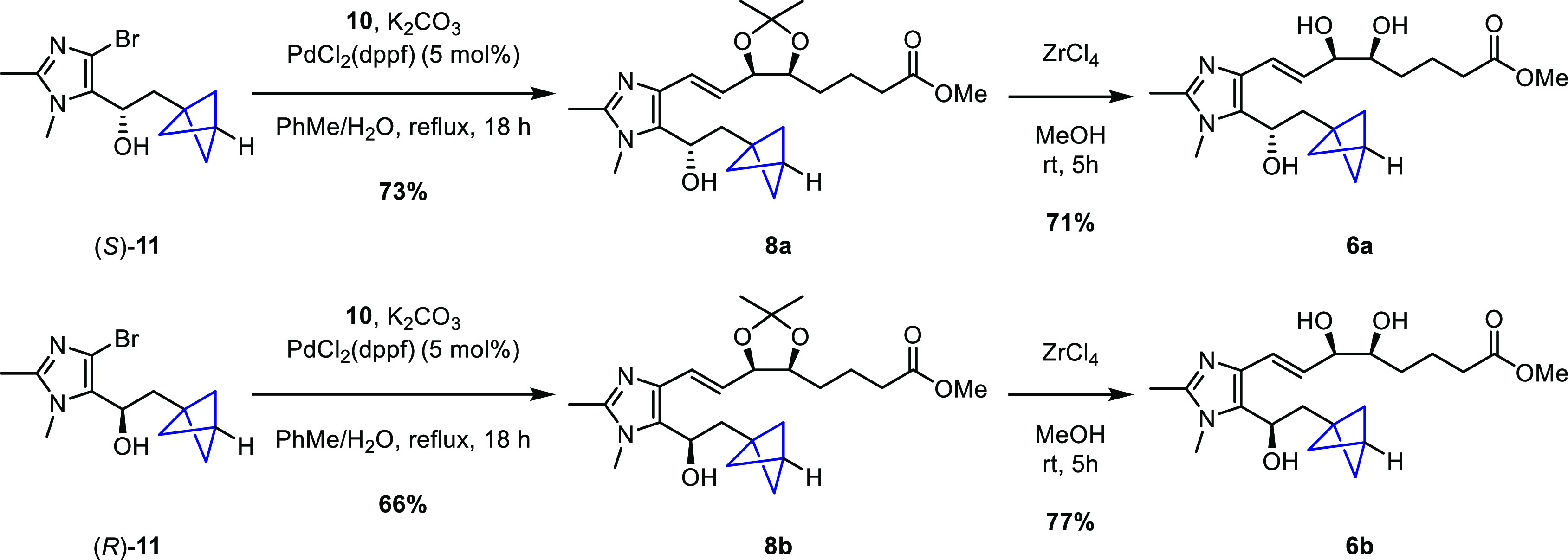
Synthesis of BCP-Containing Imidazolo-LXA_4_ Mimetics **6a** and **6b**

The four BCP-sLXms [derivatives of benzo-LXA_4_^7^ (**5a** and **5b**) or imidazolo-LXA_4_ (**6a** and **6b**)] were screened for their impact on inflammatory responses, by measuring *in vitro* NFκB activity and the downstream release of pro-inflammatory cytokines from human monocyte cell lines stably transfected for a NFκB-driven luciferase reporter (THP-1-Lucia) (Figure 2a). Concentration–response studies (ranging from 1 fM to 1 mM) showed that BCP-sLXm **6a** was the most efficacious and potent (IC_50_ in the picomolar range) anti-inflammatory compound, significantly attenuating lipopolysaccharide (LPS)-induced NFκB activity in monocytes by ∼50% and downregulating the LPS-triggered release of a series of pro-inflammatory cytokines [TNFα, MCP1, and MIP1α (see Table S15)].

**Figure 2:**
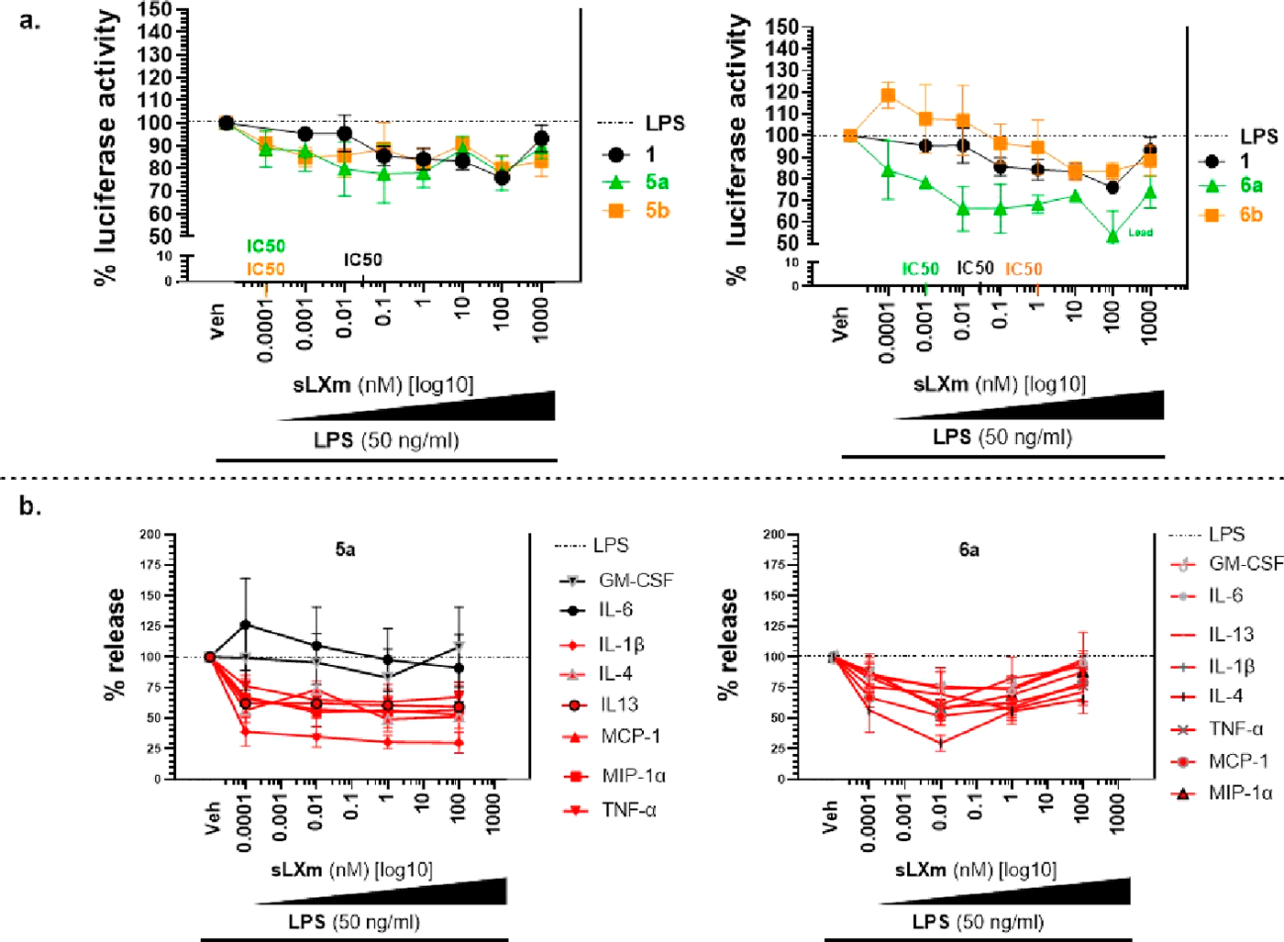
Effect of BCP-sLXms on (a) LPS-induced NFκB-driven luciferase activity in monocytes and (b) pro-inflammatory cytokine release.

In this study, the asymmetric synthesis of four novel BCP-containing sLXm analogues was successfully carried out via a modular approach relying on a Suzuki cross-coupling between a common “upper chain” and different BCP-containing “lower chains”. The data from biological evaluation clearly demonstrate the therapeutic potential of BCP-sLXms as novel inflammatory regulators.

## Supporting Information Available

Experimental procedures, characterization data, and spectral data of all compounds (PDF)
