# Supplementary material for: Synthesis and Biological Evaluation of Bicyclo[1.1.1]pentane-Containing Aromatic Lipoxin A4 Analogues
Source: Org Lett. 2022 Aug 8;24(32):6049–53. doi: 10.1021/acs.orglett.2c02345 (PMC9400386; doi:10.1021/acs.orglett.2c02345)
Supplement: Supplementary file 1 — ol2c02345_si_001.pdf [file ol2c02345_si_001.pdf]

Synthesis and Biological Evaluation of Bicyclo[1.1.1]pentane-containing Aromatic Lipoxin A$_4$ Analogs

Benjamin Owen[1], Monica de Gaetano[2], Andrew Gaffney[3], Catherine Godson[3] and Patrick J. Guiry[1]

[1] Centre for Synthesis and Chemical Biology, School of Chemistry, University College Dublin, Belfield, Dublin 4, Ireland.

[2] School of Biology & Environmental Science, Diabetes Complications Research Centre, UCD Conway Institute, University College Dublin, Belfield, Dublin 4, Ireland.

[3] School of Medicine, Diabetes Complications Research Centre, UCD Conway Institute, University College Dublin, Belfield, Dublin 4, Ireland.

Supporting Information

Table of Contents

**1.**	**General Experimental Considerations**	**S2**
**2.**	**Experimental Procedures and Characterization Data**	**S4**
**3.**	**SFC Chromatograms**	**S28**
**4.**	**X-Ray Crystallographic Data for Compounds (*S*)-11 and (*R*)-11**	**S31**
**5.**	**Biological Data**	**S51**
**6.**	**References**	**S59**
**7.**	**NMR Spectra**	**S60**

1. General Experimental Considerations

^{1}H-NMR Spectroscopy: ^{1}H and ^{13}C NMR spectra were obtained using Varian VNMRS 300, 400, 500 and 600 MHz spectrometers at room temperature. Proton and carbon chemical shifts are quoted in ppm. ^{1}H NMR spectra were recorded using an internal deuterium lock for the residual protons in $CDCl_3$ (δ 7.26). ^{13}C NMR spectra were recorded using an internal deuterium lock in $CDCl_3$ (δ 77.0). Assignments were determined either on the basis of unambiguous chemical shift or coupling patterns, COSY, HSQC and/or NOESY experiments. Peak multiplicities are defined as: s = singlet, d = doublet, t = triplet, q = quartet, m = multiplet, br. = broad; coupling constants (*J*) are reported to the nearest 0.1 Hz.

Infrared Spectroscopy: Infrared spectra were recorded on a Varian 3100 FT-IR spectrometer with the sample being prepared as a thin film on a diamond ATR module. A─rption maxima (ν_{max}) are quoted in wavenumbers (cm^{-1}).

Supercritical Fluid Chromatography: SFC was performed on a Waters UPC^2 using a Chiralcel-IA3, IB3, IC3 or ID3 column.

Mass Spectrometry: High-resolution mass spectra (HRMS) were recorded using a Waters Micromass LCT time-of-flight mass spectrometer.

Optical Rotation: Optical rotation measurements were recorded using a Schmidt-Haensch Unipol L2000 polarimeter at 589 nm and are quoted in units of deg $cm^3dm^{-1}g^{-1}$.

X-Ray Crystallography: Crystal data were collected using a Rigaku Oxford Diffraction (former Agilent Technologies, former Oxford Diffraction) SuperNova A diffractometer. A full sphere of the reciprocal space was scanned by phi-omega scans. Pseudoempirical absorption correction based on the redundant reflections was performed by the program SADABS (X-1). The structures were solved by direct methods using SHELXS-97 (X2) and refined by full matrix least-squares on F2 for all data using SHELXS-97 (X-3). All hydrogen atoms were added at calculated positions and refined using a riding model. Their isotropic temperature factors were fixed to 1.2 times (1.5 times for methyl groups) the equivalent isotropic displacement parameters of the carbon atom that the H-atom is attached to. Anisotropic temperature factors were used for all non-hydrogen atoms. Friedel opposites were merged in the refinement.

Reagents, Solvents and Techniques: Reagents were purchased from Sigma-Aldrich, Fischer, Acros or Fluorochem and used without further purification unless otherwise state. Dry tetrahydrofuran was obtained from a Puresol Grubbs system unless otherwise stated. When appropriate, reactions were performed under a nitrogen atmosphere with oven dried glassware. Oxygen free nitrogen was supplied by BOC gases and used without further drying. Column chromatography was performed with Merck Kieselgel 60 F254 (230-400 mesh) silica gel. Thin-layer chromatography was performed on aluminium sheets pre-coated plates with silica gel 60 F_{254}, or aluminium oxide 60 F_{254}. The plates were realised with ultraviolet fluorescence. Solvent was removed from solutions using a Büchi rotary evaporator with an integrated vacuum pump.

2. Experimental Procedures and Characterization Data

Scheme S1. Synthesis of "upper chain" coupling partner **10**.[1]

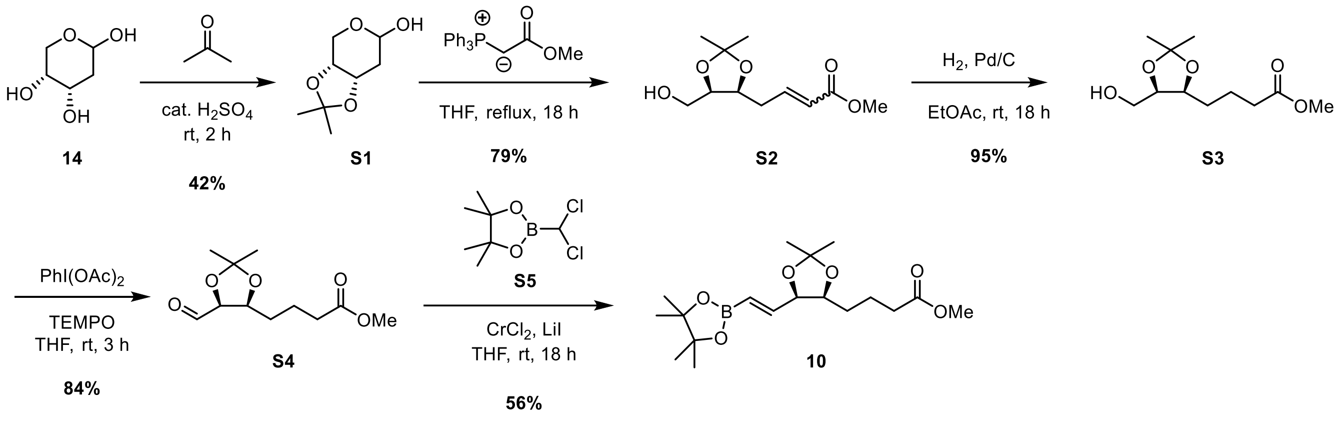

(3a*R*,7a*S*)-2,2-dimethyltetrahydro-4*H*-[1,3]dioxolo[4,5-c]pyran-6-ol (S1)

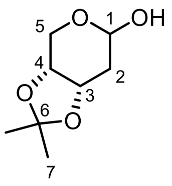

S1

To a flask equipped with stirrer bar was suspended 2-deoxy-D-ribose (5.00 g, 37.28 mmol) in acetone (125 mL). Conc. H_2SO_4 (3 drops) was added and the resulting mixture was stirred at rt for 2 h. $NaHCO_3$ was added until the pH was ~ 7, after which the solution was filtered and concentrated *in vacuo*. Purification by column chromatography (SiO_2, cyclohexane/EtOAc, 1:1) afforded the desired product as a pale yellow oil (2.76 g, 42%). The product was isolated as a mixture of α- and β- anomers in a ratio of approx. 2:1.

Minor diastereomer:

^{1}H NMR (400 MHz, $CDCl_3$) δ 5.10 – 5.02 (1H, m, H1), 4.40 (1H, dt, *J* = 5.8, 4.7 Hz, H3), 4.23 – 4.11 (1H, m, H4), 3.97 – 3.94 (1H, m, H5), 3,90 (1H, broad s , -OH), 3.73 – 3.66 (1H, m, H5), 2.15 – 1.99 (2H, m, H2),1.56 (3H, s, H7), 1.35 (3H, s, H7). **^{13}C NMR** (101 MHz, $CDCl_3$) δ 109.5, 91.6, 71.3, 70.8, 60.8, 32.5, 28.1, 25.7.

Major diastereomer:

^{1}H NMR (400 MHz, $CDCl_3$) δ 5.25 (1H, dd, *J* = 7.1, 4.3 Hz, H1), 4.47 (1H, dt, *J* = 6.6, 4.3 Hz, H3), 4.23 – 4.11 (1H, m, H4), 3.94 – 3.91 (1H, m, H5), 3.74 – 3.65 (1H, m, H5), 3.04 (1H, broad s, -OH), 2.23 (1H, dt, *J* = 14.8, 4.3 Hz, H2), 1.77 (ddd, *J* = 14.8, 7.1, 4.3 Hz, H2), 1.49 (3H, s, H7), 1.34 (3H, s, H7). **^{13}C NMR** (101 MHz, $CDCl_3$) δ 108.9, 91.1, 71.8, 70.5, 62.2, 32.3, 27.4, 25.5.
Spectroscopic data in agreement with those previously reported.[1]

methyl-4-((4S,5R)-5-(hydroxymethyl)-2,2-dimethyl-1,3-dioxolan-4-yl)but-2-enoate (S2)

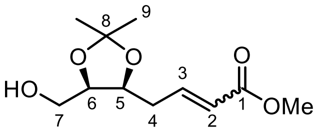

S2

To a round-bottom flask was added **S1** (2.46 g, 14.1 mmol) and methyl (triphenylphosphoranylidene)acetate (5.67 g, 17.0 mmol) in dry THF (45 mL). The resulting mixture was refluxed at 66 °C in an oil bath for 18 h. The solution was then concentrated *in vacuo* and the residue was re-dissolved in EtOAc (20 mL). The resulting solution was washed with H_2O (2 x 20 mL) and brine (20 mL) then dried over $MgSO_4$, filtered, and concentrated *in vacuo*. Purification by column chromatography (SiO_2, cyclohexane/EtOAc, 3:2) afforded the desired product as a yellow oil (2.57 g, 79%). The product was obtained as a mixture of geometric isomers with an *E*/*Z* ratio of approx. 5:1.

R_f = 0.24 (cyclohexane/EtOAc 3:2)[$KMnO_4$]

E-isomer:

^{1}H NMR (400 MHz, $CDCl_3$) δ 6.97 (1H, dt, *J* = 15.8, 6.9 Hz, H3), 5.93 (1H, dt, *J* = 15.8, 1.6 Hz, H2), 4.34 – 4.25 (1H, m, H5), 4.24 – 4.13 (1H, m, H6), 3.72 (3H, s, -OMe), 3.65 (2H, t, *J* = 5.7 Hz, 2H) 2.58 – 2.48 (1H, m, H4), 2.48 – 2.39 (1H, m, H4), 1.96 (1H, dd, *J* = 6.3, 5.7 Hz, -OH), 1.47 (3H, s, H9), 1.35 (3H, s, H9). **^{13}C NMR** (101 MHz, $CDCl_3$) δ 166.6, 144.8, 123.2, 108.5, 77.5, 75.3, 61.4, 51.5, 32.4, 27.9, 25.3.

Z-isomer:

^{1}H NMR (400 MHz, $CDCl_3$) δ 6.35 (1H, ddd, *J* = 11.5, 7.9, 6.6 Hz, H3), 5.88 (1H, dt, *J* = 11.5, 1.8 Hz, H2), 4.32 – 4.25 (1H, m, H5), 4.24 – 4.17 (1H, m, H6), 3.70 (3H, s, -OMe), 3.70 – 3.66 (2H, m, H7), 3.07 (1H, dddd, *J* = 15.6, 7.9, 4.1, 1.8 Hz, H4), 2.79 (1H, dddd, *J* = 15.6, 9.3, 6.6, 1.8 Hz, H4), 2.04 (1H, t, *J* = 6.1 Hz, -OH), 1.47 (3H, s, H9), 1.35 (3H, s, H9). **^{13}C NMR** (101 MHz, $CDCl_3$) δ 166.7, 146.0, 120.9, 108.5, 77.8, 76.3, 61.5, 51.2, 29.2, 28.0, 25.4.

Spectroscopic data in agreement with those previously reported.[1]

methyl 4-((4S,5R)-5-(hydroxymethyl)-2,2-dimethyl-1,3-dioxolan-4-yl)butanoate (S3)

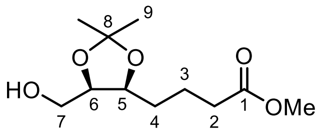

S3

To a round-bottom flask was added **S2** (2.67 g, 11.6 mmol) in EtOAc (20 mL). Pd/C (~10 wt. %, 1.2 g) was added and the flask was stoppered. The flask was evacuated and the reaction mixture was placed under an atmosphere of H_2 gas using a balloon. The resulting mixture was stirred at rt for 18 h. The mixture was then filtered through a plug of silica and concentrated *in vacuo* to afford the desired compound as a yellow oil (2.57 g, 95%).

R_f = 0.28 (CyHex/EtOAc, 3:2) [$KMnO_4$] **^{1}H NMR** (400 MHz, $CDCl_3$) δ 4.21 – 4.09 (2H, m, H5, H6), 3.66 (3H, s, -OMe), 3.64 – 3.56 (2H, m, H7), 2.46 – 2.27 (2H, m, H2), 1.97 (1H, dd, *J* = 7.0, 5.2 Hz, -OH), 1.91 – 1.78 (1H, m, H3), 1.77 – 1.65 (1H, m, H3), 1.64 – 1.48 (2H, m, H4), 1.45 (3H, s, H9), 1.38 – 1.30 (3H, m, H9). **^{13}C NMR** (101 MHz, $CDCl_3$) δ 174.0, 108.3, 78.0, 76.8, 61.8, 51.7, 33.8, 28.5, 28.3, 25.6, 22.3.

Spectroscopic data in agreement with those previously reported.[1]

methyl 4-((4S,5S)-5-formyl-2,2-dimethyl-1,3-dioxolan-4-yl)butanoate (S4)

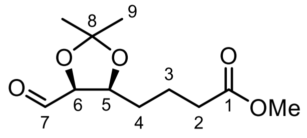

S4

To a round-bottom flask was added **S3** (2.57 g, 11.1 mmol) in CH_2Cl_2 (30 mL). TEMPO (0.170 g, 1.11 mmol) and (diacetoxyiodo)benzene (4.28 g, 13.3 mmol) were added to the flask in a single portion. The resulting mixture was stirred vigorously at rt for 3 h. The solution was then concentrated *in vacuo*. Purification by column chromatography (SiO_2, cyclohexane/EtOAc, 4:1) afforded the desired compound as an orange oil (2.13 g, 84%).

R_f = 0.24 (cyclohexane/EtOAc, 4:1) [$KMnO_4$] **^{1}H NMR** (400 MHz, $CDCl_3$) δ 9.64 (1H, dd, J = 3.3, 0.7 Hz, H7), 4.38 – 4.30 (1H, m, H5), 4.26 (1H, dd, J = 7.1, 3.3 Hz, H6), 3.67 (3H, s, -OMe), 2.35 (2H, t, J = 7.4 Hz, H2), 1.91 – 1.78 (1H, m, H3), 1.77 – 1.68 (1H, m, H3), 1.68 – 1.56 (1H, m, H4), 1.56 – 1.45 (1H, m, H4), 1.58 (3H, s, H9), 1.41 (3H, s, H9). **^{13}C NMR** (101 MHz, $CDCl_3$) δ 202.0, 173.4, 110.5, 81.8, 78.1, 51.4, 33.3, 29.0, 27.4, 25.1, 21.8.

Spectroscopic data in agreement with those previously reported.[1]

2-(dichloromethyl)-4,4,5,5-tetramethyl-1,3,2-dioxaborolane (S5)

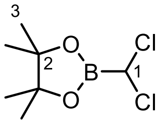

S5

To a flame-dried two-necked flask under N_2 was added dry dichloromethane (3.2 mL, 50.0 mmol) in dry THF (70 mL). The solution was cooled to −100 ˚C and *n*-BuLi (2.5 M in hexanes, 20.0 mL, 50.0 mmol) was added dropwise over 20 min. The resulting mixture was stirred at −100 ˚C for 30 min. Trimethylborate (5.77 mL, 50.0 mmol) was added in a single portion and the resulting mixture was stirred at −100 ˚C for a further 30 min. HCl (5 M, 50 mL) was added and the mixture was stirred vigorously and allowed to warm to rt. The product was extracted into Et_2O (3 x 50 mL), and the combined organic layers were dried over $MgSO_4$, filtered, and concentrated *in vacuo*. The residue was then dissolved in toluene (70 mL) and transferred to a flame-dried two-necked flask under N_2. With backflow of N_2, pinacol (5.91 g, 50.0 mmol) was added and the resulting mixture was stirred under reflux for 48 h. The resulting solution was concentrated *in vacuo*. Purification by reduced pressure distillation (b.p. 64 – 66 ˚C, 0.2 mbar) afforded the desired compound as a colorless oil (6.47 g, 61%).

^{1}H NMR (400 MHz, $CDCl_3$) δ 5.34 (1H, s, H1), 1.33 (12H, s, H3). **^{13}C NMR** (101 MHz, $CDCl_3$) δ 85.9, 24.6, (C1 gives a very broad signal between δ 57.5 and 52.2 barely detectable above the baseline).

Spectroscopic data in agreement with those previously reported.[1]

methyl 4-((4S,5R)-2,2-dimethyl-5-((E)-2-(4,4,5,5-tetramethyl-1,3,2-dioxaborolan-2-yl)vinyl)-1,3-dioxolan-4-yl)butanoate (10)

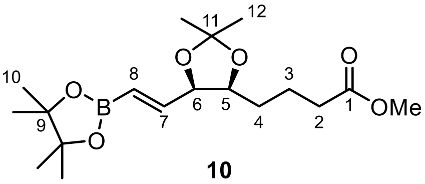

10

To a flame-dried Schlenk tube under N_2 was added **S4** (1.56 g, 6.77 mmol) and boronic ester **S5** (2.86 g, 13.6 mmol) in dry THF (70 mL). A solution of LiI (3.63 g, 27.1 mmol) in dry THF (20 mL) was added to the mixture dropwise. With backflow of N_2, $CrCl_2$ (5.00 g, 40.7 mmol) was added and the Schlenk tube immediately stoppered. The resulting mixture was stirred at rt for 18 h. The mixture was then poured onto ice water (100 mL) and extracted into dichloromethane (3 x 100 mL). The combined organic layers were dried over $MgSO_4$, filtered and concentrated *in vacuo*. Purification by column chromatography (SiO_2, cyclohexane/EtOAc, 9:1) afforded the desired compound as a pale yellow oil (1.34 g, 56%).

R_f = 0.29 (cyclohexane/EtOAc, 4:1) [PMA] **^{1}H NMR** (400 MHz, $CDCl_3$) δ 6.47 (1H, dd, *J* = 18.0, 6.8 Hz, H7), 5.69 (1H, dd, *J* = 18.0, 1.3 Hz, H8), 4.52 (1H, td, *J* = 6.4, 1.2 Hz, H6), 4.15 (1H, ddd, *J* = 9.2, 6.4, 4.4 Hz, H5), 3.65 (3H, s, -OMe), 2.34 (2H, td, *J* = 7.6, 1.9 Hz, H2), 1.87 – 1.73 (1H, m, H3), 1.72 – 1.59 (1H, m, H3), 1.55 – 1.37 (2H, m, H4), 1.47 (3H, s, H12), 1.34 (3H, s, H12), 1.25 (12H, s, H10). **^{13}C NMR** (101 MHz, $CDCl_3$) δ 173.9, 148.1, 121.9 (br), 108.6, 83.5, 80.5, 78.1, 51.6, 33.9, 30.0, 28.2, 25.8, 24.9, 24.8, 21.9.

Spectroscopic data in agreement with those previously reported.[1]

[1.1.1]propellane (13)

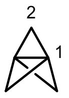

13

According to the procedure described by Gianatassio et al.[2] To a flame-dried flask equipped with a stirrer bar was added 1,1-dibromo-2,2-bis(chloromethyl)cyclopropane (14.0 g, 47.2 mmol). The reaction vessel was placed under N_2, and anhydrous Et_2O (30 mL) was added. The reaction vessel was cooled to −45 °C. Phenyllithium (1.9 M in Bu_2O, 50.0 mL, 94.3 mmol) was added dropwise over 15 min at –45 °C, and the resulting mixture was stirred for 15 min at −45 °C. The cooling bath was replaced with an ice bath, and the reaction mixture was warmed to 0 °C and stirred at this temperature for 2 h. The mixture was then distilled at room temperature using a rotary evaporator, the receiving flask of which was immersed in a dry ice/acetone bath. The distillate was transferred in a flame-dried septum-sealed bottle under inert atmosphere, and stored at −20 °C. The approximate yield was determined by ^{1}H NMR spectroscopy with 1,2-dichloroethane as an internal standard. The concentration of the [1.1.1]propellane solution ranged between 0.62 M and 1.10 M, with yields of 45-61%.

^{1}H NMR (300 MHz, $CDCl_3$) δ 1.94 (6H, s, H2).

Spectroscopic data in agreement with those previously reported.[2]

1-(2-bromophenyl)-2-iodoethan-1-one (12)

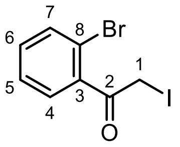

12

To a flask containing the 2'-bromoacetophenone (1.00 mL, 7.44 mmol) in MeCN (30 mL) was added N-bromosuccinimide (1.46 g, 8.18 mmol) and *p*-toluenesulfonic acid (1.41 g, 8.18 mmol). The resulting mixture was stirred at 80 °C in an oil bath for 18 h. After cooling to rt, the mixture was concentrated *in vacuo*. $NaHCO_3$ (aq. sat., 40 mL) was added and the product was extracted into dichloromethane (3 x 30 mL). The combined organic layers were dried over $MgSO_4$, filtered and concentrated *in vacuo*. The residue was dissolved in acetone (30 mL) and sodium iodide (1.23 g, 8.18 mmol) was added. The mixture was stirred at rt for 30 min. Water (30 mL) was added and the product was extracted into dichloromethane (3 x 10 mL). The combined organic layers were dried over $MgSO_4$, filtered, and concentrated *in vacuo*. Purification by column chromatography (SiO_2, cyclohexane/Et_2O, 19:1) afforded the desired compound as a pale yellow oil (1.64 g, 68%).

R_f = 0.28 (cyclohexane/Et_2O, 19:1) [UV/$KMnO_4$] **^{1}H NMR** (400 MHz, $CDCl_3$) δ 7.62 (1H, dd, *J* = 7.7, 1.4 Hz, H7), 7.50 (1H, dd, *J* = 7.6, 1.8 Hz, H4), 7.42 – 7.30 (2H, m, H5, H6), 4.42 (2H, s, H1). **^{13}C NMR** (101 MHz, $CDCl_3$) δ 196.3, 138.6, 133.8, 132.5, 130.3, 127.6, 119.3, 6.1.

Spectroscopic data in agreement with those previously reported.[3]

2-(bicyclo[1.1.1]pentan-1-yl)-1-(2-bromophenyl)ethan-1-one (9) and 2-(bicyclo[1.1.1]pentan-1-yl)-1-phenylethan-1-one (18)

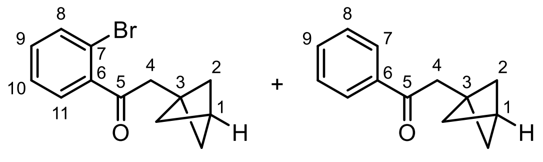

9 **18**

To a flask containing **12** (0.650 g, 2.00 mmol) in Et_2O (2 mL) was added [1.1.1]propellane (0.55 M in Et_2O, 4.73 mL, 2.60 mmol). Triethylborane (1.0 M in hexanes, 0.20 mL, 0.20 mmol) was syringed directly into the solution and the resulting mixture was stirred at rt for 90 min. The solution was concentrated *in vacuo* to afford crude **17** which was immediately dissolved in dichloromethane (5 mL). Tris(trimethylsilyl)silane (0.80 mL, 2.60 mmol) was added followed by triethylborane (1.0 M in hexanes, 0.20 mL, 0.20 mmol) and the resulting mixture was stirred at rt for 30 min. More triethylborane (1.0 M in hexanes, 0.20 mL, 0.20 mmol) was added and the mixture was stirred at rt for a further 1 h. The mixture was then washed with $NaHCO_3$ (aq. sat., 3 x 5 mL) and the organic layer was dried over $MgSO_4$, filtered, and concentrated *in vacuo*. Purification by column chromatography (SiO_2, cyclohexane/Et_2O, 49:1) afforded the product as a colorless oil. The product was obtained as a 2.5:1 mixture of the **9** (~0.147 g, 28%) to **18** (~0.042 g, 11%).

Mixture: $\mathbf{R_f}$ = 0.52 (pentane/Et_2O, 9:1) [UV/PMA] **IR** (film) ν_{max}/cm^{-1} 2967, 2907, 2871, 1695, 1587, 1428, 1288, 1257, 1218, 1200, 1000, 765, 621.

Compound 9: **^{1}H NMR** (400 MHz, $CDCl_3$) δ 7.60 (1H, ddd, *J* = 7.9, 1.1, 0.6 Hz, H8), 7.41 – 7.32 (2H, m, H10, H11), 7.28 (1H, ddd, *J* = 7.9, 6.6, 2.5 Hz, H9), 3.11 (2H, s, H4), 2.46 (1H, s, H1), 1.78 (6H, s, H2). **^{13}C NMR** (101 MHz, $CDCl_3$) δ 202.5, 141.9, 134.0, 131.7, 129.0, 127.5, 119.2, 51.8, 45.9, 41.3, 28.9. **HRMS** (ESI) $[M+H]^+$ calc. 265.0223 for $[C_{13}H_{14}{}^{79}BrO]^+$; found 265.0222, calc. 267.0203 for $[C_{13}H_{14}{}^{81}BrO]^+$; found 267.0202.

Compound 23: **^{1}H NMR** (400 MHz, $CDCl_3$) δ 7.94 – 7.87 (2H, m, H7), 7.58 – 7.51 (1H, m, H9), 7.49 – 7.42 (2H, m, H8), 3.11 (2H, s, H4), 2.47 (1H, s, H1), 1.78 (6H, s, H2). **^{13}C NMR** (101 MHz, $CDCl_3$) δ 198.9, 137.4, 133.1, 128.7, 128.6, 119.2, 51.8, 42.3, 41.6, 28.5.

methyl 4-((4*S*,5*R*)-5-((*E*)-2-(2-(bicyclo[1.1.1]pentan-1-yl)acetyl)styryl)-2,2-dimethyl-1,3-dioxolan-4-yl)butanoate (7)

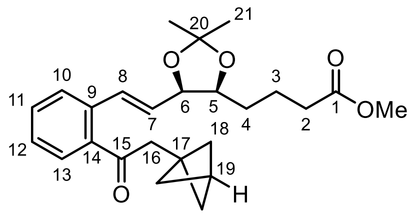

7

To a microwave vial was added **10** (0.162 g, 0.457 mmol) and **9** (0.146 g, 0.549 mmol) in 1,2-dimethoxyethane (2 mL). $Pd(PPh_3)_4$ (0.0264 g, 0.0229 mmol) was added followed by K_2CO_3 (2.0 M in H_2O, 0.46 mL, 0.92 mmol). The vial was placed in a microwave reactor and heated at 125 °C for 50 min. The mixture was then diluted with dichloromethane (10 mL) and washed with H_2O (2 x 10 mL) and brine (10 mL). The organic layer was dried over $MgSO_4$, filtered and concentrated *in vacuo*. Purification by column chromatography (SiO_2, cyclohexane/EtOAc, 9:1) afforded the desired compound as a pale yellow oil (0.116 g, 62%).

R_f = 0.11 (cyclohexane/EtOAc, 9:1) [UV/PMA] **^{1}H NMR** (400 MHz, $CDCl_3$) δ 7.59 – 7.50 (2H, m, H10, H13), 7.43 (1H, td, *J* = 7.4, 0.9 Hz, H11), 7.32 (1H, td, *J* = 7.6, 1.3 Hz, H12), 7.06 (1H, d, *J* = 15.8 Hz, H8), 6.03 (1H, dd, *J* = 15.8, 8.2 Hz, H7), 4.69 (1H, ddd, *J* = 8.2, 6.2, 1.0 Hz, H6), 4.21 (1H, ddd, *J* = 8.9, 6.2, 4.5 Hz, H5), 3.63 (3H, s, -OMe), 3.03 (2H, s, H16), 2.45 (1H, s, H19), 2.34 (2H, t, *J* = 7.4 Hz, H2), 1.91 – 1.78 (1H, m, H3), 1.73 (6H, s, H18), 1.77 – 1.45 (3H, m, H3, H4), 1.52 (3H, s, H21), 1.39 (3H, s, H21). **^{13}C NMR** (101 MHz, $CDCl_3$) δ 203.0, 173.9, 138.1, 136.4, 132.2, 131.5, 128.8, 128.5, 127.9, 127.5, 108.5, 79.6, 78.5, 51.8, 51.6, 45.6, 41.7, 34.0, 30.1, 28.7, 28.5, 25.8, 21.9. **$[\alpha]_D$** = –9.29 ° (c = 1.09 g/100 cm^3, $CHCl_3$) **IR** (film) ν_{max}/cm^{-1} 2965, 2908, 2871, 1737, 1684, 1436, 1379, 1250, 1217, 1166. **HRMS** (ESI) $[M+Na]^+$ calc. 435.2142 for $[C_{25}H_{32}O_5Na]^+$; found 435.2145.

methyl 4-((4*S*,5*R*)-5-((*E*)-2-((*S*)-2-(bicyclo[1.1.1]pentan-1-yl)-1-hydroxyethyl)styryl)-2,2-dimethyl-1,3-dioxolan-4-yl)butanoate (19a)

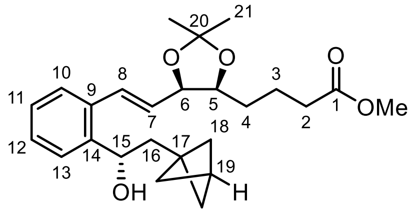

19a

To a flame-dried Schlenk tube under N_2 was added **7** (0.090 g, 0.218 mmol) in dry Et_2O (4 mL). The solution was cooled to –20 °C and (-)-DIP-chloride (0.126 g, 0.393 mmol) was added with backflow of N_2. The resulting mixture was stirred at –20 °C for 72 h. Diethanolamine (1 mL) was then added and the mixture was stirred at rt for a further 4 h. Cyclohexane (5 mL) was added and the resulting suspension was filtered under suction. The residue was washed with Et_2O (3 x 5 mL) and the filtrate was concentrated *in vacuo*. Purification by column chromatography (SiO_2, cyclohexane/EtOAc, 4:1) afforded the desired compound as a pale yellow oil (0.064 g, 71%, *dr* 99:1).

R_f = 0.25 (cyclohexane/EtOAc, 4:1) [UV/PMA] **^{1}H NMR** (400 MHz, $CDCl_3$) δ 7.47 (1H, dd, *J* = 7.6, 1.5 Hz, H13), 7.41 (1H, dd, *J* = 7.5, 1.6 Hz, H10), 7.31 – 7.18 (2H, m, H11, H12), 6.92 (1H, d, *J* = 15.6 Hz, H8), 6.01 (1H, dd, *J* = 15.6, 7.7 Hz, H7), 5.03 (1H, t, *J* = 6.5 Hz, H15), 4.69 (1H, ddd, *J* = 7.7, 6.2, 1.1 Hz, H6), 4.21 (1H, ddd, *J* = 8.6, 6.2, 4.9 Hz, H5), 3.61 (3H, s, -OMe), 2.46 (1H, s, H19), 2.42 – 2.26 (2H, m H2), 2.03 (1H, s, -OH), 1.90 – 1.83 (2H, m, H16), 1.91 – 1.75 (1H, m, H3), 1.72 (6H, s, H18), 1.78 – 1.63 (1H, m, H3), 1.65 – 1.42 (2H, m, H4), 1.53 (3H, s, H21), 1.40 (3H, s, H21). **^{13}C NMR** (101 MHz, $CDCl_3$) δ 174.0, 142.0, 134.3, 130.2, 128.3, 128.2, 127.6, 126.7, 125.8, 108.5, 79.6, 78.4, 69.6, 51.7, 51.3, 43.7, 41.0, 33.9, 30.2, 28.5, 28.4, 25.8, 21.8. **$[\alpha]_D$** = –32.74 ° (c = 1.07 g/100 cm^3, $CHCl_3$). **IR** (film) ν_{max}/cm^{-1} 3467 (broad), 2959, 2906, 2868, 1737, 1479, 1379, 1246, 1216, 1166, 1044, 970, 759. **HRMS** (ESI) $[M+Na]^+$ calc. 437.2298 for $[C_{25}H_{34}O_5Na]^+$; found 437.2304. *dr* = 99:1 as determined by SFC (chiralpak ID column, sCO_2:0.2% NH_4OH in MeOH, 99:1 for 0 – 1 min, gradient to 40:60 for 1 – 5 min, 3.00 mL/min, T = 35 ˚C); R_T = 3.00 min [(*S*)-major], R_T = 3.32 min [(*R*)-minor].

methyl 4-((4*S*,5*R*)-5-((*E*)-2-((*R*)-2-(bicyclo[1.1.1]pentan-1-yl)-1-hydroxyethyl)styryl)-2,2-dimethyl-1,3-dioxolan-4-yl)butanoate (19b)

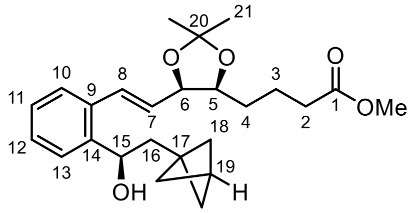

19b

To a flame-dried Schlenk tube under N_2 was added **7** (0.116 g, 0.281 mmol) in dry Et_2O (5 mL). The solution was cooled to –20 °C and (+)-DIP-chloride (0.162 g, 0.506 mmol) was added with backflow of N_2. The resulting mixture was stirred at –20 °C for 72 h. Diethanolamine (1 mL) was then added and the mixture was stirred at rt for a further 4 h. Cyclohexane (5 mL) was added and the resulting suspension was filtered under suction. The residue was washed with Et_2O (3 x 5 mL) and the filtrate was concentrated *in vacuo*. Purification by column chromatography (SiO_2, cyclohexane/EtOAc, 4:1) afforded the desired compound as a pale yellow oil (0.075 g, 64%, *dr* 98:2).

^{1}H NMR (400 MHz, $CDCl_3$) δ 7.46 (1H, dd, *J* = 7.6, 1.6 Hz, H13), 7.41 (1H, dd, *J* = 7.5, 1.6 Hz, H10), 7.32 – 7.19 (2H, m, H11, H12), 6.93 (1H, d, *J* = 15.7 Hz, H8), 6.00 (1H, dd, *J* = 15.7, 7.7 Hz, H7), 5.02 (1H, dd, *J* = 8.3, 4.5 Hz, H15), 4.69 (1H, ddd, *J* = 7.7, 6.2, 1.1 Hz, H6), 4.22 (1H, ddd, *J* = 8.9, 6.2, 4.6 Hz, H5), 3.63 (3H, s, -OMe), 2.47 (1H, s, H19), 2.39 – 2.29 (2H, m, H2), 2.00 (1H, s, -OH), 1.97 – 1.77 (3H, m, H3, H16), 1.72 (6H, d, *J* = 2.1 Hz, H18), 1.77 – 1.63 (1H, m, H3), 1.64 – 1.42 (2H, m, H4), 1.53 (3H, s, H21), 1.40 (3H, s, H21). **^{13}C NMR** (101 MHz, $CDCl_3$) δ 173.9, 142.0, 134.4, 130.5, 128.3, 128.2, 127.6, 126.9, 125.8, 108.5, 79.6, 78.5, 70.0, 51.7, 51.3, 43.7, 41.2, 34.0, 30.3, 28.5, 28.4, 25.8, 21.9. **$[\alpha]_D$** = +70.92 ° (c = 1.21 g/100 cm^3, $CHCl_3$). **IR** (film) ν_{max}/cm^{-1} 3467 (broad), 2959, 2906, 2868, 1737, 1479, 1379, 1245, 1215, 1166, 1044, 970, 759. **HRMS** (ESI) $[M+Na]^+$ calc. 437.2298 for $[C_{25}H_{34}O_5Na]^+$; found 437.2299. *dr* = 49:1 as determined by SFC (chiralpak ID column, sCO_2:0.2% NH_4OH in MeOH, 99:1 for 0 – 1 min, gradient to 40:60 for 1 – 5 min, 3.00 mL/min, T = 35 ˚C); R_T = 2.95 min [(*S*)-minor], R_T = 3.27 min [(*R*)-major].

methyl (5*S*,6*R*,*E*)-8-(2-((*S*)-2-(bicyclo[1.1.1]pentan-1-yl)-1-hydroxyethyl)phenyl)-5,6-dihydroxyoct-7-enoate (5a)

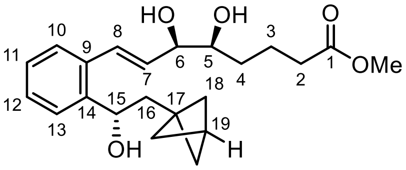

5a

To a vial containing **19a** (0.031 g, 0.075 mmol) in MeOH (2 mL) was added camphorsulfonic acid (0.017 g, 0.075 mmol) and the resulting mixture was stirred at rt for 3 h. The solution was then concentrated *in vacuo* at room temperature. Purification by column chromatography (SiO_2, EtOAc/cyclohexane, 3:2) afforded the desired compound as a pale yellow oil (0.0125 g, 45%).

R_f = 0.20 (EtOAc/cyclohexane, 3:2) [UV/PMA] **^{1}H NMR** (500 MHz, $CDCl_3$) δ 7.46 – 7.38 (2H, m, H10, H13), 7.31 – 7.19 (2H, m, H11, H12), 6.98 (1H, d, *J* = 15.8 Hz, H8), 6.10 (1H, dd, *J* = 15.8, 6.9 Hz, H7), 5.01 (1H, dd, *J* = 7.8, 5.2 Hz, H15), 4.27 (1H, ddd, *J* = 6.9, 3.8, 1.3 Hz, H6), 3.76 (1H, dt, *J* = 8.9, 3.8 Hz, H5), 3.65 (3H, s, -OMe), 2.45 (1H, s, H19), 2.40 – 2.31 (2H, m, H2), 1.95 – 1.78 (3H, m, H3, H16), 1.77 – 1.63 (1H, m, H3), 1.70 (6H, s, H18), 1.58 – 1.42 (2H, m, H4). **^{13}C NMR** (126 MHz, $CDCl_3$) δ 174.4, 141.7, 134.6, 130.2, 129.9, 128.1, 127.7, 126.8, 126.0, 76.0, 74.1, 70.0, 51.8, 51.3, 43.6, 40.9, 33.9, 31.6, 28.4, 21.2. **$[\alpha]_D$**= –8.84° (c = 1.25 g/100 cm^3, $CHCl_3$). **IR** (film) ν_{max}/cm^{-1} 3397 (broad), 2958, 2925, 2868, 1720, 1438, 1197, 1049, 1006, 970, 759. **HRMS** (ESI) $[M+Na]^+$ calc. 397.1985 for $[C_{22}H_{30}O_5Na]^+$; found 397.1991.

methyl (5*S*,6*R*,*E*)-8-(2-((*R*)-2-(bicyclo[1.1.1]pentan-1-yl)-1-hydroxyethyl)phenyl)-5,6-dihydroxyoct-7-enoate (5b)

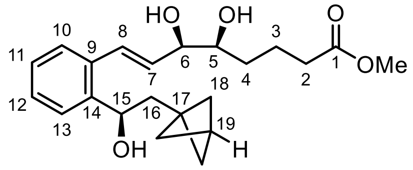

5b

To a vial containing **19b** (0.032 g, 0.077 mmol) in MeOH (2 mL) was added camphorsulfonic acid (0.018 g, 0.077 mmol) and the resulting mixture was stirred at rt for 3 h. The solution was then concentrated *in vacuo* at room temperature. Purification by column chromatography (SiO_2, EtOAc/cyclohexane, 3:2) afforded the desired compound as a pale yellow oil (0.0075 g, 26%).

R_f = 0.20 (EtOAc/cyclohexane, 3:2) [UV/PMA] **^{1}H NMR** (500 MHz, $CDCl_3$) δ 7.48 – 7.39 (2H, m, H10, H13), 7.32 – 7.21 (2H, m, H11, H12), 6.97 (1H, d, *J* = 15.8 Hz, H8), 6.12 (1H, dd, *J* = 15.8, 6.7 Hz, H7), 5.01 (1H, dd, *J* = 7.9, 5.0 Hz, H15), 4.27 (1H, dd, *J* = 6.7, 3.6 Hz, H6), 3.77 (1H, dt, *J* = 8.2, 3.6 Hz, H5), 3.65 (3H, s, -OMe), 2.46 (1H, s, H19), 2.39 – 2.33 (2H, m, H2), 1.94 – 1.81 (3H, m, H3, H16), 1.79 – 1.66 (1H, m, H3), 1.70 (6H, d, *J* = 1.3 Hz, H18), 1.58 – 1.42 (2H, m, H4). **^{13}C NMR** (126 MHz, $CDCl_3$) δ 174.3, 141.7, 134.6, 130.4, 129.9, 128.2, 127.7, 126.8, 125.8, 76.0, 74.0, 69.9, 51.8, 51.3, 43.6, 40.9, 33.9, 31.7, 28.4, 21.3. **$[\alpha]_D$** = +11.28 ° (c = 0.85 g/100 cm^3, $CHCl_3$). **IR** (film) ν_{max}/cm^{-1} 3396 (broad), 2959, 2924, 2868, 1721, 1438, 1197, 1051, 1005, 970, 759. **HRMS** (ESI) $[M+Na]^+$ calc. 397.1985 for $[C_{22}H_{30}O_5Na]^+$; found 397.1988.

4,5-dibromo-1,2-dimethyl-1H-imidazole (21)

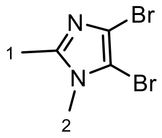

21

To a round-bottom flask was added 1,2-dimethylimidazole (2.50 g, 26.0 mmol) in $CHCl_3$ (100 mL). N-Bromosuccinimide (10.20 g, 57.2 mmol) was added portionwise and the resulting mixture was stirred at rt for 24 h. HCl (5 M, 50 mL) was added and the organic layer was extracted into H_2O (3 x 50 mL). The combined aqueous layers were neutralized with NaOH (aq., 5 M, 50 mL) and the resulting suspension was collected via suction filtration. The residue was washed with H_2O (3 x 20 mL) and dried in vacuo for 24 h to afford the desired compound as a white solid (4.32 g, 65%).

^{1}H NMR (400 MHz, $CDCl_3$) δ 3.52 (3H, s, H2), 2.38 (3H, s, H1). **^{13}C NMR** (101 MHz, $CDCl_3$) δ 145.7, 114.8, 103.1, 32.9, 14.3.

Spectroscopic data in agreement with those previously reported.[4]

1-(1,2,4-trimethyl-1H-imidazol-5-yl)ethan-1-one (22)

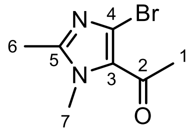

22

To a flame-dried Schlenk tube under N_2 was added **21** (2.00 g, 7.88 mmol) in dry THF (15 mL). The solution was cooled to –78 °C and *n*-BuLi (2.5 M in hexanes, 3.15 mL, 7.88 mmol) was added dropwise. The resulting mixture was stirred at –78 °C for 5 min then transferred via cannula to a round-bottom flask containing acetyl chloride (2.81 mL, 39.4 mmol) in dry THF (15 mL) pre-cooled to –78 °C. The resulting mixture was stirred at –78 °C for 1 h then warmed to rt and stirred for a further 1 h. EtOH (10 mL) was added and the mixture was stirred for 10 min. $NaHCO_3$ (aq. sat., 40 mL) was added and the product was extracted into dichloromethane (3 x 40 mL). The combined organic layers were dried over $MgSO_4$, filtered, and concentrated *in vacuo*. Purification by column chromatography (SiO_2, cyclohexane/EtOAc, 1:1) afforded the desired compound as a pale-yellow solid (1.64 g, 48%).

R_f = 0.28 (Cyclohexane/EtOAc 2:3) [UV/$KMnO_4$] **^{1}H NMR** (400 MHz, $CDCl_3$) δ 3.79 (3H, s, H7), 2.63 (3H, s, H1), 2.39 (3H, s, H6). **^{13}C NMR** (101 MHz, $CDCl_3$) δ 188.7, 150.1, 128.8, 123.8, 34.3, 30.8, 13.5.

Spectroscopic data in agreement with those previously reported.[4]

1-(4-bromo-1,2-dimethyl-1H-imidazol-5-yl)-2-iodoethan-1-one (15)

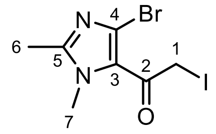

15

To a flask containing **27** (2.82 g, 13.0 mmol) in MeCN (50 mL) was added N-Bromosuccinimide (2.54 g, 14.3 mmol) and *p*-toluenesulfonic acid (3.36 g, 19.5 mmol). The resulting mixture was stirred at 80 °C in an oil bath for 18 h. After cooling to rt, the mixture was concentrated *in vacuo*. $NaHCO_3$ (aq. sat., 40 mL) was added and the product was extracted into dichloromethane (3 x 30 mL). The combined organic layers were dried over $MgSO_4$, filtered and concentrated *in vacuo*. The residue was dissolved in acetone (50 mL) and sodium iodide (2.34 g, 15.6 mmol) was added. The mixture was stirred at rt for 30 min. Water (50 mL) was added and the product was extracted into dichloromethane (3 x 30 mL). The combined organic layers were dried over $MgSO_4$, filtered and concentrated *in vacuo*. Purification by column chromatography (SiO_2, cyclohexane/EtOAc, 1:1) afforded the desired compound as an orange solid (1.88 g, 42%).

R_f = 0.22 (cyclohexane/EtOAc, 1:1) [UV/$KMnO_4$] **^{1}H NMR** (400 MHz, $CDCl_3$) δ 4.50 (2H, s, H1), 3.80 (3H, s, H7), 2.44 (3H, s, H6). **^{13}C NMR** (101 MHz, $CDCl_3$) δ 183.5, 151.4, 125.9, 124.1, 34.5, 13.7, 6.7. **m.p.** = 61 – 62 °C (decomp.). **IR** (film) ν_{max}/cm^{-1} 1754, 1646, 1490. 1461, 1341, 1257, 1019, 982. **HRMS** (ESI) $[M+H]^+$ calc. 342.8938 for $[C_7H_9{}^{79}Br^{127}IN_2O]^+$; found 342.8958, calc. 344.8917 for $[C_7H_9{}^{81}Br^{127}IN_2O]^+$; found 344.8955.

2-(bicyclo[1.1.1]pentan-1-yl)-1-(4-bromo-1,2-dimethyl-1H-imidazol-5-yl)ethan-1-one (24)

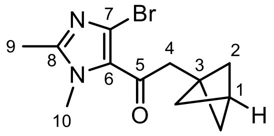

24

To a round-bottom flask containing **15** (1.00 g, 2.92 mmol) in Et_2O (5 mL) was added [1.1.1]propellane (0.62 M in Et_2O, 6.11 mL, 3.79 mmol). Triethylborane (1.0 M in hexanes, 0.29 mL, 0.29 mmol) was syringed directly into the mixture, the flask was stoppered, and the resulting mixture was stirred at rt for 90 min. The reaction mixture was then concentrated *in vacuo* to afford crude **23** which was immediately dissolved in dichloromethane (20 mL). Tris(trimethylsilyl)silane (1.17 mL, 3.79 mmol) was added followed by triethylborane (1.0 M in hexanes, 0.29 mL, 0.29 mmol) and the resulting mixture was stirred at rt for 30 min. More triethylborane (1.0 M in hexanes, 0.29 mL, 0.29 mmol) was added and the reaction was stirred for a further 1 h. The solution was then washed with $NaHCO_3$ (aq. sat., 3 x 10 mL), dried over Na_2SO_4, filtered, and concentrated *in vacuo*. Purification by column chromatography (SiO_2, cyclohexane/EtOAc, 1:1) afforded the desired compound as a yellow oil (0.279 g, 34%).

R_f = 0.34 (cyclohexane/EtOAc, 1:1) [UV/PMA] **^{1}H NMR** (400 MHz, $CDCl_3$) δ 3.77 (3H, s, H10), 3.21 (2H, s, H4), 2.47 (1H, s, H1), 2.38 (3H, s, H9), 1.81 (6H, s, H2). **^{13}C NMR** (101 MHz, $CDCl_3$) δ 189.9, 150.0, 128.8, 123.1, 51.8, 44.2, 41.7, 34.3, 29.0, 13.6. **IR** (film) ν_{max}/cm^{-1} 2966, 2907, 2871, 1652, 1488, 1464, 1362, 1252, 1228, 1194. **HRMS** (ESI) $[M+H]^+$ calc. 283.0441 for $[C_{12}H_{16}{}^{79}BrN_2O]^+$; found 283.0454, calc. 285.0421 for $[C_{12}H_{16}{}^{81}BrN_2O]^+$; found 285.0472.

(*S*)-2-(bicyclo[1.1.1]pentan-1-yl)-1-(4-bromo-1,2-dimethyl-1H-imidazol-5-yl)ethan-1-ol [(*S*)-11]

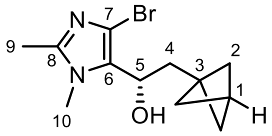

(*S*)-**11**

To a vial containing **24** (0.140 g, 0.494 mmol) in dry *i*-PrOH (3 mL) was added $RuCl_2$[(*R*)-DM-BINAP][(*R*)-DAIPEN] (0.0302 g, 0.0247 mmol) followed by potassium *tert*-butoxide (0.055 g, 0.494 mmol). A few drops of triisopropyl borate were added and the vial was placed in a Parr hydrogenator. The hydrogenator was flushed with H_2 (3 x 20 bar) and the reaction mixture was stirred under H_2 (20 bar) at rt for 72 h. Water (5 mL) was then added and the product was extracted into dichloromethane (3 x 5 mL). The combined organic layers were dried over Na_2SO_4, filtered and concentrated *in vacuo*. Purification by column chromatography (SiO_2, 100% EtOAc) afforded the desired product as a white solid (0.091 g, 65%). The product was recrystallized from $CHCl_3$ by vapour diffusion of pentane to afford crystals with 98% *ee* (0.069 g, 49%). Crystals suitable for X-ray diffraction were grown from $CHCl_3$ by slow vapour diffusion of pentane.

R_f = 0.27 (100% EtOAc) [PMA] **^{1}H NMR** (500 MHz, $CDCl_3$) δ 4.87 (1H, td, *J* = 7.5, 3.1 Hz, H5), 3.62 (3H, s, H10), 2.43 (1H, s, H1), 2.34 (1H, d, *J* = 3.1 Hz, -OH), 2.31 (3H, s, H9), 2.11 (1H, dd, *J* = 14.3, 7.5 Hz, H4), 1.96 (1H, dd, *J* = 14.3, 7.5 Hz, H4), 1.69 (3H, dd, *J* = 9.5, 1.7 Hz, H2), 1.63 (3H, dd, *J* = 9.5, 1.7 Hz, H2). **^{13}C NMR** (101 MHz, $CDCl_3$) δ 146.0, 129.4, 112.8, 77.5, 64.9, 51.0, 43.0, 38.2, 32.1, 28.3, 13.3. **m.p.** = 193 – 194 °C. **$[\alpha]_D$** = -23.57 ° (c = 1.04 g/100 cm^3, $CHCl_3$). **IR** (film) ν_{max}/cm^{-1} 3160 (broad), 2961, 2922, 2904, 2870, 1464, 1402, 1347, 1242, 1196, 1036, 998, 774, 709. **HRMS** (ESI) $[M+H]^+$ calc. 285.0598 for $[C_{12}H_{18}{}^{79}BrN_2O]^+$; found 285.0602, calc. 287.0577 for $[C_{12}H_{18}{}^{81}BrN_2O]^+$; found 287.0583. *ee* = 98% as determined by SFC (chiralpak IC column, sCO_2:MeOH, 99:1 for 0 – 1 min, gradient to 60:40 for 1 – 5 min, 3.00 mL/min, T = 35 ˚C); R_T = 3.58 min [(*R*)-minor], R_T = 3.67 min [(*S*)-major].

(*R*)-2-(bicyclo[1.1.1]pentan-1-yl)-1-(4-bromo-1,2-dimethyl-1H-imidazol-5-yl)ethan-1-ol [(*R*)-11]

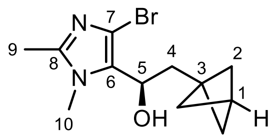

(*R*)-**11**

To a vial containing **24** (0.140 g, 0.494 mmol) in dry *i*-PrOH (3 mL) was added $RuCl_2$[(*S*)-DM-BINAP][(*S*)-DAIPEN] (0.0302 g, 0.0247 mmol) followed by potassium *tert*-butoxide (0.055 g, 0.494 mmol). A few drops of triisopropyl borate were added and the vial was placed in a Parr hydrogenator. The hydrogenator was flushed with H_2 (3 x 20 bar) and the reaction mixture was stirred under H_2 (20 bar) at rt for 72 h. Water (5 mL) was added and the product was extracted into dichloromethane (3 x 5 mL). The combined organic layers were dried over Na_2SO_4, filtered and concentrated *in vacuo*. Purification by column chromatography (100% EtOAc) afforded the desired product as a white solid (0.069 g, 49%). The product was recrystallized from $CHCl_3$ by vapour diffusion of pentane to afford crystals with 99% *ee* (0.048 g, 34%). Crystals suitable for X-ray diffraction were grown from $CHCl_3$ by slow vapour diffusion of pentane.

R_f = 0.27 (100% EtOAc) [PMA] **^{1}H NMR** (500 MHz, $CDCl_3$) δ 4.86 (1H, td, *J* = 7.5, 2.9 Hz, H5), 3.62 (3H, s, H10), 2.48 (1H, d, *J* = 2.9 Hz, -OH), 2.43 (1H, s, H1), 2.31 (3H, s, H9), 2.10 (1H, dd, *J* = 14.3, 7.5 Hz, H4), 1.96 (1H, dd, *J* = 14.3, 7.5 Hz, H4), 1.69 (3H, dd, *J* = 9.5, 1.7 Hz, H2), 1.62 (3H, dd, *J* = 9.5, 1.7 Hz, H2). **^{13}C NMR** (126 MHz, $CDCl_3$) δ 146.0, 129.3, 112.9, 64.9, 51.0, 42.9, 38.2, 32.0, 28.3, 13.3. **m.p.** = 193 – 194 °C. **$[\alpha]_D$** = +18.65 ° (c = 0.98 g/100 cm^3, $CHCl_3$). **IR** (film) ν_{max}/cm^{-1} 3160 (broad), 2961, 2922, 2904, 2869, 1464, 1402, 1347, 1242, 1196, 1036, 998, 774, 709. *ee* = 99% as determined by SFC (chiralpak IC column, sCO_2:MeOH, 99:1 for 0 – 1 min, gradient to 60:40 for 1 – 5 min, 3.00 mL/min, T = 35 ˚C); R_T = 3.57 min [(*R*)-major], R_T = 3.67 min [(*S*)-minor].

methyl 4-((4*S*,5*R*)-5-((*E*)-2-(5-((*S*)-2-(bicyclo[1.1.1]pentan-1-yl)-1-hydroxyethyl)-1,2-dimethyl-1H-imidazol-4-yl)vinyl)-2,2-dimethyl-1,3-dioxolan-4-yl)butanoate [8a]

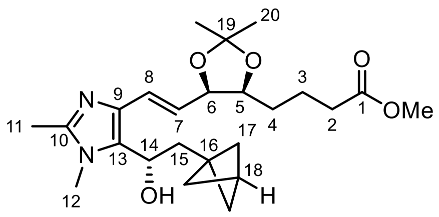

8a

To a Schlenk tube under N_2 was added (*S*)-**11** (0.029 g, 0.102 mmol), **10** (0.040 g, 0.112 mmol) and $PdCl_2$(dppf)•CH_2Cl_2 (0.0042 g, 0.0051 mmol) in toluene (1 mL). K_2CO_3 (2.0 M in H_2O, 0.15 mL, 0.31 mmol) was added and the resulting mixture was refluxed at 110 °C in an oil bath for 18 h. The mixture was then diluted with dichloromethane (10 mL) and washed with H_2O (2 x 10 mL) followed by brine (10 mL). The organic layer was dried over $MgSO_4$, filtered and concentrated *in vacuo*. Purification by column chromatography (SiO_2, CH_2Cl_2/MeOH, 19:1) afforded the desired compound as a pale orange oil (0.032 g, 73%).

R_f = 0.41 (CH_2Cl_2/MeOH, 19:1) [UV/PMA] **^{1}H NMR** (400 MHz, $CDCl_3$) δ 6.40 (1H, dd, *J* = 15.4, 1.1 Hz, H8), 6.18 (1H, dd, *J* = 15.4, 6.8 Hz, H7), 4.88 (1H, t, *J* = 7.4 Hz, H14), 4.65 (1H, t, *J* = 6.8 Hz, H6), 4.15 (1H, ddd, *J* = 8.9, 6.2, 4.5 Hz, H5), 3.61 (3H, s, -OMe), 3.56 (3H, s, H12), 2.40 (1H, s, H18), 2.35 – 2.27 (2H, m, H2), 2.25 (3H, s, H11), 2.08 (1H, dd, *J* = 14.3, 7.4 Hz, H15), 1.85 (1H, d, *J* = 14.3, 7.4 Hz, H15), 1.81 – 1.73 (1H, m, H3), 1.63 (3H, dd, *J* = 9.6, 1.6 Hz, H17), 1.58 (3H, dd, *J* = 9.5, 1.6 Hz, H17), 1.72 – 1.41 (3H, m, H3, H4), 1.49 (3H, s, H20), 1.37 (3H, s, H20). **^{13}C NMR** (101 MHz, $CDCl_3$) δ 174.1, 146.3, 133.8, 130.3, 123.0, 122.7, 108.2, 79.5, 78.5, 63.9, 51.6, 50.9, 43.1, 38.4, 34.0, 31.8, 30.2, 28.4, 28.4, 25.9, 22.0, 13.2. **$[\alpha]_D$** = –2.24 ° (c = 1.03 g/100 cm^3, $CHCl_3$). **IR** (film) ν_{max}/cm^{-1} 3170 (broad), 2958, 2907, 2867, 1736, 1436, 1406, 1370, 1244, 1215, 1198, 1026, 972. **HRMS** (ESI) M^+ calc. 432.2619 for $[C_{24}H_{36}N_2O_5]^+$; found 432.2619.

methyl 4-((4*S*,5*R*)-5-((*E*)-2-(5-((*R*)-2-(bicyclo[1.1.1]pentan-1-yl)-1-hydroxyethyl)-1,2-dimethyl-1H-imidazol-4-yl)vinyl)-2,2-dimethyl-1,3-dioxolan-4-yl)butanoate [8b]

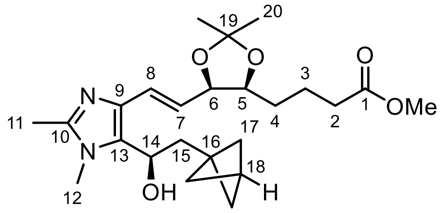

8b

To a Schlenk tube under N_2 was added (*R*)-**11** (0.030 g, 0.105 mmol), **10** (0.041 g, 0.116 mmol) and $PdCl_2$(dppf)•CH_2Cl_2 (0.0043 g, 0.0053 mmol) in toluene (1 mL). K_2CO_3 (2.0 M in H_2O, 0.16 mL, 0.32 mmol) was added and the resulting mixture was refluxed at 110 °C in an oil bath for 18 h. The mixture was then diluted with dichloromethane (10 mL) and washed with H_2O (2 x 10 mL) followed by brine (10 mL). The organic layer was dried over $MgSO_4$, filtered and concentrated *in vacuo*. Purification by column chromatography (SiO_2, CH_2Cl_2/MeOH, 19:1) afforded the desired compound as a pale orange oil (0.030 g, 66%).

R_f = 0.38 (CH_2Cl_2/MeOH, 19:1) [UV/PMA] **^{1}H NMR** (400 MHz, $CDCl_3$) δ 6.35 (1H, dd, *J* = 15.4, 0.8 Hz, H8), 6.14 (1H, dd, *J* = 15.4, 8.3 Hz, H7), 4.86 (1H, t, *J* = 7.5 Hz, H14), 4.59 (1H, dd, *J* = 8.3, 6.1 Hz, H6), 4.13 (1H, ddd, *J* = 8.8, 6.1, 4.3 Hz, H5), 3.61 (3H, s, -OMe), 3.54 (3H, s, H12), 2.37 (1H, s, H18), 2.34 – 2.24 (2H, m, H2), 2.19 (3H, s, H11), 2.07 (1H, dd, *J* = 14.4, 7.5 Hz, H15), 1.85 (1H, dd, *J* = 14.4, 7.5 Hz, H15), 1.81 – 1.72 (1H, m, H3), 1.59 (3H, dd, *J* = 9.6, 1.6 Hz, H17), 1.54 (3H, dd, *J* = 9.6, 1.6 Hz, H17), 1.70 – 1.39 (3H, m, H3, H4), 1.49 (3H, s, H20), 1.37 (3H, s, H20). **^{13}C NMR** (101 MHz, $CDCl_3$) δ 174.0, 146.1, 133.6, 130.7, 124.2, 122.3, 108.2, 80.4, 78.5, 63.4, 51.6, 50.9, 43.1, 38.3, 33.9, 31.8, 30.1, 28.5, 28.3, 25.9, 21.9, 13.0. **$[\alpha]_D$** = +13.15° (c = 1.07 g/100 cm^3, $CHCl_3$). **IR** (film) ν_{max}/cm^{-1} 3165 (broad), 2959, 2907, 2867, 1737, 1436, 1406, 1378, 1369, 1320, 1215, 1198, 1165, 1028, 972, 879. **HRMS** (ESI) $[M+H]^+$ calc. 433.2697 for $[C_{24}H_{37}N_2O_5]^+$; found 433.2708.

methyl (5*S*,6*R*,*E*)-8-(5-((*S*)-2-(bicyclo[1.1.1]pentan-1-yl)-1-hydroxyethyl)-1,2-dimethyl-1H-imidazol-4-yl)-5,6-dihydroxyoct-7-enoate [6a]

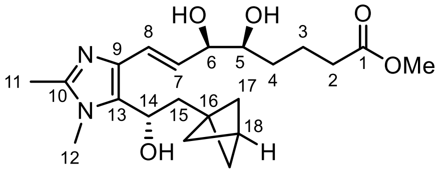

6a

To a vial containing **8a** (0.031 g, 0.072 mmol) in MeOH (1 mL) was added $ZrCl_4$ (0.0084 g, 0.036 mmol). The resulting mixture was stirred at rt for 7 h. The solution was then concentrated *in vacuo* at room temperature. Purification by column chromatography (SiO_2, CH_2Cl_2/MeOH, 9:1) afforded the desired compound as a pale yellow oil (0.020 g, 71%).

R_f = 0.19 (CH_2Cl_2/MeOH, 9:1) [UV/PMA] **^{1}H NMR** (500 MHz, $CDCl_3$) δ 6.44 (1H, dd, *J* = 15.7, 1.3 Hz, H8), 6.24 (1H, dd, *J* = 15.7, 6.5 Hz, H7), 4.87 (1H, t, *J* = 7.5 Hz, H14), 4.13 (1H, dd, *J* = 6.5, 2.9 Hz, H6), 3.65 – 3.60 (1H, m, H5), 3.63 (3H, s, -OMe), 3.57 (3H, s, H12), 2.38 (1H, s, H18), 2.29 (3H, s, H11), 2.32 – 2.26 (2H, m, H2), 2.07 (1H, dd, *J* = 14.3, 7.5 Hz, H15), 1.88 (1H, dd, *J* = 14.3, 7.5 Hz, H15), 1.85 – 1.75 (1H, m, H3), 1.70 – 1.57 (1H, m, H3), 1.60 (3H, dd, *J* = 9.6, 1.7 Hz, H17), 1.57 – 1.49 (1H, m, H4), 1.55 (3H, dd, *J* = 9.5, 1.7 Hz, H17), 1.48 – 1.37 (1H, m, H4). **^{13}C NMR** (126 MHz, $CDCl_3$) δ 174.5, 146.0, 133.4, 130.3, 126.8, 121.9, 75.8, 74.3, 63.6, 51.7, 50.9, 43.1, 38.4, 34.0, 31.8, 31.7, 28.4, 21.5, 12.9. **$[\alpha]_D$** = –12.10 ° (c = 0.95 g/100 cm^3, $CHCl_3$). **IR** (film) ν_{max}/cm^{-1} 3354 (broad), 2959, 2907, 2868, 1735, 1436, 1407, 1198, 1078, 1035, 1005, 972, 754. **HRMS** (ESI) $[M+H]^+$ calc. 393.2384 for $[C_{21}H_{33}N_2O_5]^+$; found 393.2386.

methyl (5*S*,6*R*,*E*)-8-(5-((*R*)-2-(bicyclo[1.1.1]pentan-1-yl)-1-hydroxyethyl)-1,2-dimethyl-1H-imidazol-4-yl)-5,6-dihydroxyoct-7-enoate [6b]

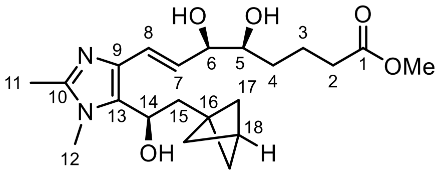

6b

To a vial containing **8b** (0.030 g, 0.069 mmol) in MeOH (1 mL) was added $ZrCl_4$ (0.0081 g, 0.035 mmol). The resulting mixture was stirred at rt for 7 h. The solution was then concentrated *in vacuo* at room temperature. Purification by column chromatography (SiO_2, CH_2Cl_2/MeOH, 9:1) afforded the desired compound as a pale yellow oil (0.022 g, 77%).

R_f = 0.22 (CH_2Cl_2/MeOH, 9:1) [UV/PMA] **^{1}H NMR** (500 MHz, $CDCl_3$) δ 6.43 (1H, d, *J* = 15.7 Hz, H8), 6.28 (1H, dd, *J* = 15.6, 6.6 Hz, H7), 4.87 (1H, t, *J* = 7.6 Hz, H14), 4.09 (1H, dd, *J* = 6.8, 3.7 Hz, H6), 3.68 – 3.64 (1H, m, H5), 3.63 (3H, s, -OMe), 3.59 (3H, s, H12), 2.37 (1H, s, H18), 2.30 (3H, s, H11), 2.32 – 2.26 (2H, m, H2), 2.07 (1H, dd, *J* = 14.3, 7.6 Hz, H15), 1.90 (1H, dd, *J* = 14.3, 7.6 Hz, H15), 1.86 – 1.75 (1H, m, H3), 1.69 – 1.53 (1H, m, H3), 1.58 (3H, dd, *J* = 9.6, 1.7 Hz, H17), 1.52 (3H, dd, *J* = 9.6, 1.7 Hz, H17), 1.55 – 1.37 (2H, m, H4). **^{13}C NMR** (126 MHz, $CDCl_3$) δ 174.4, 146.2, 133.6, 130.2, 126.4, 122.2, 76.0, 74.4, 63.4, 51.7, 50.9, 43.0, 38.3, 34.0, 32.0, 31.9, 28.4, 21.5, 12.9. **$[\alpha]_D$** = +5.71 ° (c = 1.05 g/100 cm^3, $CHCl_3$). **IR** (film) ν_{max}/cm^{-1} 3372 (broad), 2960, 2907, 2868, 1736, 1522, 1436, 1407, 1198, 1078, 1033, 1005, 972. **HRMS** (ESI) $[M+H]^+$ calc. 393.2384 for $[C_{21}H_{33}N_2O_5]^+$; found 393.2387.

3. SFC Chromatograms

3.1 Compounds 19a and 19b

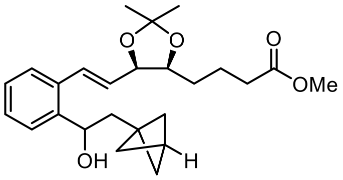

19a + 19b

	Processed Channel	Retention Time (min)	Area	% Area	Height
1	PDA 246.9 nm	2.785	2078433	48.83	1190535
2	PDA 246.9 nm	3.133	2177868	51.17	1213365

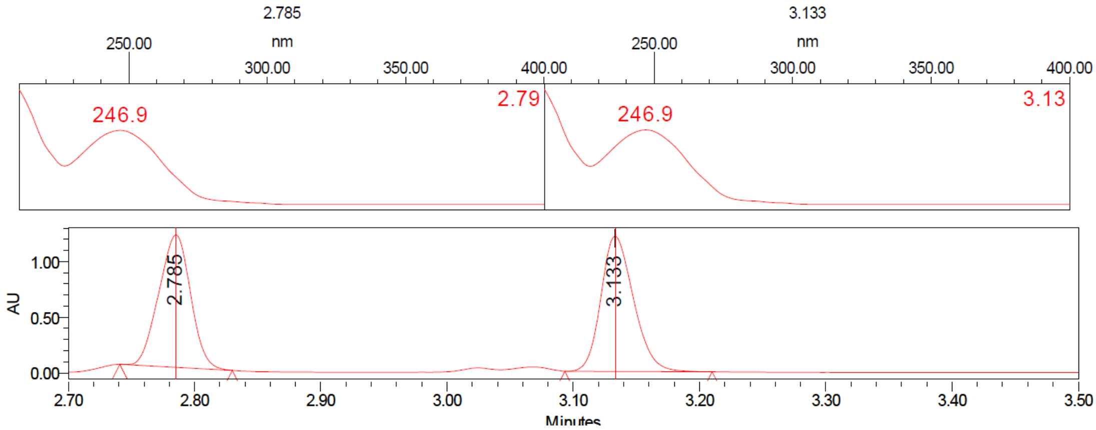

Figure S1. SFC (chiralpak ID column, sCO_2:0.2% NH_4OH in MeOH, 99:1 for 0 – 1 min, gradient to 40:60 for 1 – 5 min, 3.00 mL/min, T = 35 ˚C); R_T = 2.79 min [(*S*)-epimer], R_T = 3.13 min [(*R*)-epimer].

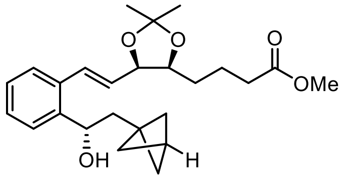

19a

	Processed Channel	Retention Time (min)	Area	% Area	Height
1	PDA 246.9 nm	3.003	4300055	99.22	2867726
2	PDA 246.9 nm	3.324	33818	0.78	24622

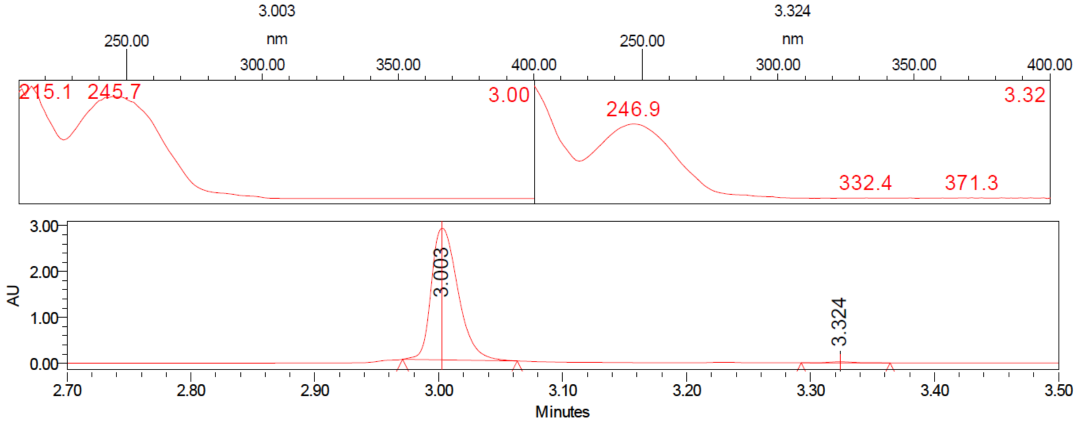

Figure S2. *dr* = 99:1 as determined by SFC (chiralpak ID column, sCO_2:0.2% NH_4OH in MeOH, 99:1 for 0 – 1 min, gradient to 40:60 for 1 – 5 min, 3.00 mL/min, T = 35 ˚C); R_T = 3.00 min [(*S*)-major], R_T = 3.32 min [(*R*)-minor].

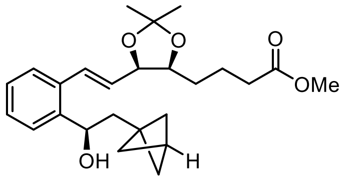

19b

	Processed Channel	Retention Time (min)	Area	% Area	Height
1	PDA 246.9 nm	2.949	65716	2.01	27057
2	PDA 246.9 nm	3.274	3208355	97.99	1546324

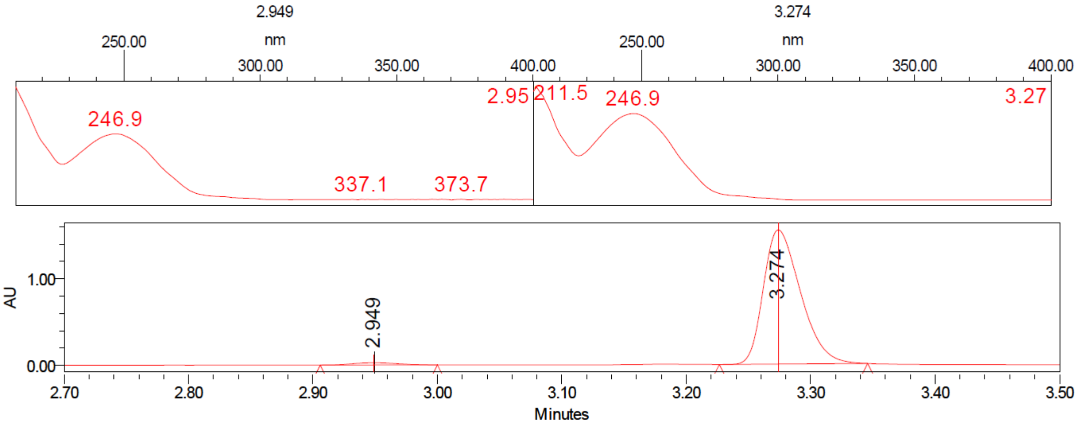

Figure S3. *dr* = 49:1 as determined by SFC (chiralpak ID column, sCO_2:0.2% NH_4OH in MeOH, 99:1 for 0 – 1 min, gradient to 40:60 for 1 – 5 min, 3.00 mL/min, T = 35 ˚C); R_T = 2.95 min [(*S*)-minor], R_T = 3.27 min [(*R*)-major].

3.2 Compounds *rac*-11, (*S*)-11 and (*R*)-11

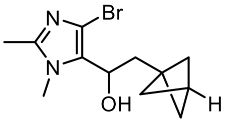

rac-**11**

	Processed Channel	Retention Time (min)	Area	% Area	Height
1	PDA 218.6 nm	3.573	27359	55.11	13984
2	PDA 218.6 nm	3.667	22283	44.89	11631

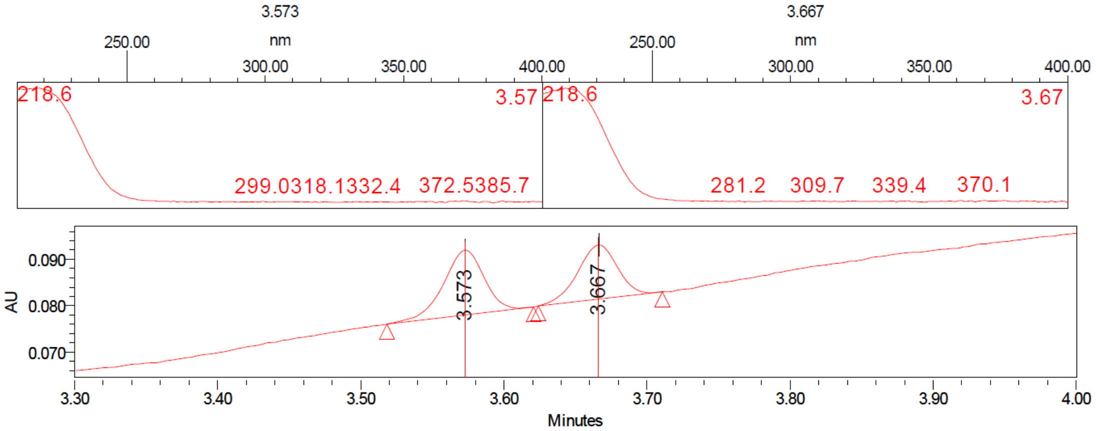

Figure S4. SFC (chiralpak IC column, sCO_2:MeOH, 99:1 for 0 – 1 min, gradient to 60:40 for 1 – 5 min, 3.00 mL/min, T = 35 ˚C); R_T = 3.58 min [(*R*)-enantiomer], R_T = 3.67 min [(*S*)-enantiomer].

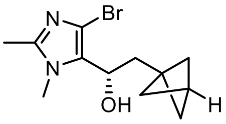

(*S*)-**11**

	Processed Channel	Retention Time (min)	Area	% Area	Height
1	PDA 218.6 nm	3.579	3414	0.80	1912
2	PDA 218.6 nm	3.665	424316	99.20	223479

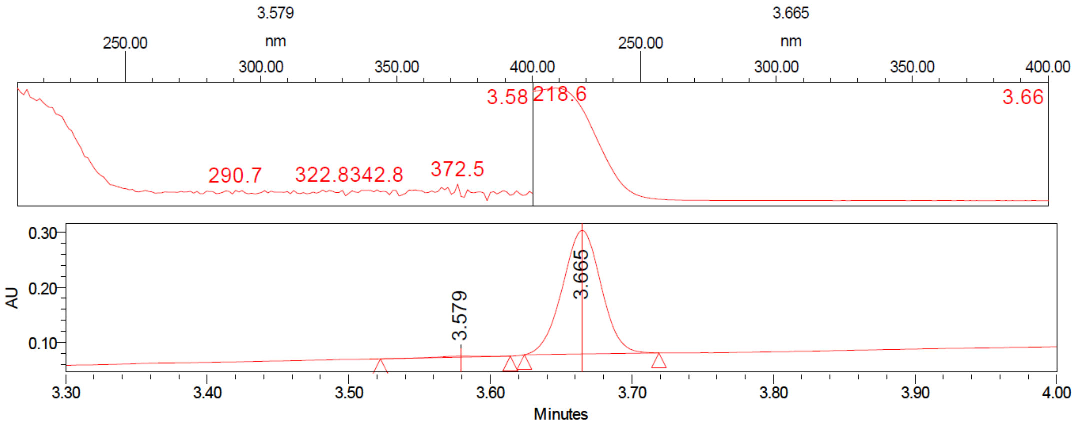

Figure S5. *ee* = 98% as determined by SFC (chiralpak IC column, sCO_2:MeOH, 99:1 for 0 – 1 min, gradient to 60:40 for 1 – 5 min, 3.00 mL/min, T = 35 ˚C); R_T = 3.58 min [(*R*)-minor], R_T = 3.67 min [(*S*)-major].

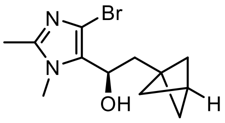

(*R*)-**11**

	Processed Channel	Retention Time (min)	Area	% Area	Height
1	PDA 218.6 nm	3.572	460449	99.51	251671
2	PDA 218.6 nm	3.668	2257	0.49	1261

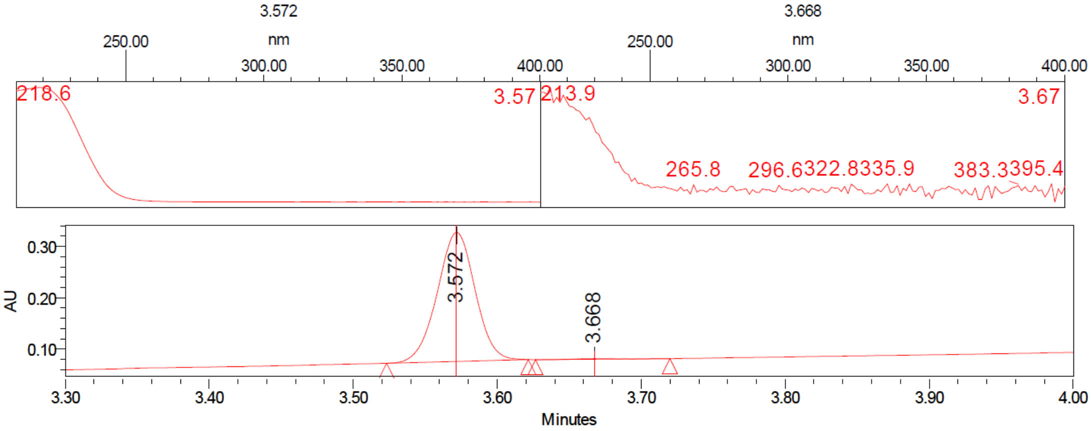

Figure S6. *ee* = 99% as determined by SFC (chiralpak IC column, sCO_2:MeOH, 99:1 for 0 – 1 min, gradient to 60:40 for 1 – 5 min, 3.00 mL/min, T = 35 ˚C); R_T = 3.57 min [(*R*)-major], R_T = 3.67 min [(*S*)-minor].

4. X-ray Crystallographic Data for Compounds (*S*)-11 and (*R*)-11

4.1 (*S*)-11

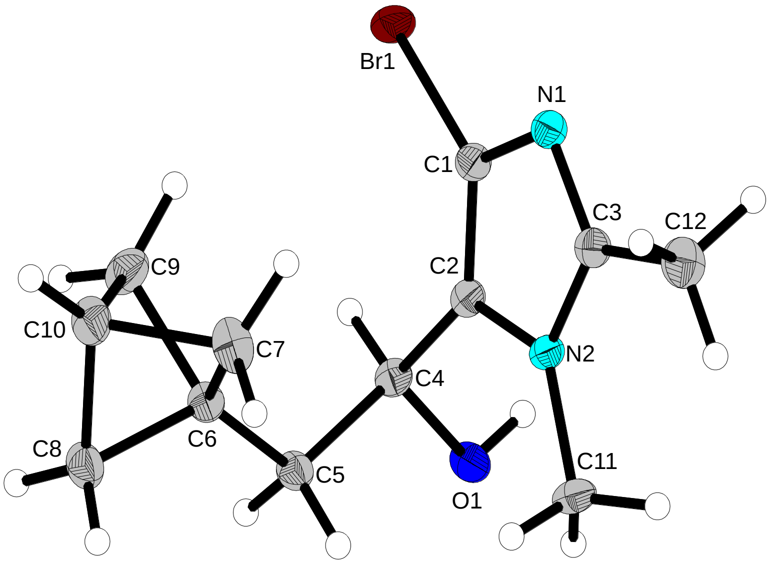

(*S*)-**11**, molecule; thermal ellipsoids are drawn on the 50% level.

Table S1. Crystal data and structure refinement for (*S*)-**11**.

Identification code	gui198	
Empirical formula	$C_{12} H_{17} N_2 O Br$	
Formula weight	285.18	
Temperature	100(2) K	
Wavelength	1.54184 Å	
Crystal system	Orthorhombic	
Space group	$P2_12_12_1$ (#19)	
Unit cell dimensions	a = 8.79582(8) Å	α = 90°.
	b = 9.8976(1) Å	β = 90°.
	c = 14.1690(2) Å	γ = 90°.
Volume	1233.52(2) $Å^3$	
Z	4	
Density (calculated)	1.536 Mg/m^3	
Absorption coefficient	4.391 mm^{-1}	
F(000)	584	
Crystal size	0.190 x 0.120 x 0.110 mm^3	
Theta range for data collection	5.452 to 76.770°.	

Index ranges	–11<=h<=11, –12<=k<=12, –17<=l<=15
Reflections collected	12794
Independent reflections	2578 [R(int) = 0.0209]
Completeness to theta = 67.684°	100.0 %
Absorption correction	Semi–empirical from equivalents
Max. and min. transmission	1.00000 and 0.80002
Refinement method	Full–matrix least–squares on F^2
Data / restraints / parameters	2578 / 0 / 148
Goodness–of–fit on F^2	1.058
Final R indices [I>2sigma(I)]	R1 = 0.0161, wR2 = 0.0408
R indices (all data)	R1 = 0.0165, wR2 = 0.0412
Absolute structure parameter	–0.031(6)
Extinction coefficient	n/a
Largest diff. peak and hole	0.190 and –0.364 e.$Å^{-3}$

Table S2. Atomic coordinates (x 10^4) and equivalent isotropic displacement parameters ($Å^2$x 10^3)
for (*S*)-**11**. U(eq) is defined as one third of the trace of the orthogonalized U^{ij} tensor.

Atom	x	y	z	U(eq)
N(1)	7471(2)	8426(2)	5208(1)	16(1)
C(1)	7974(2)	7196(2)	4897(1)	16(1)
Br(1)	7858(1)	6770(1)	3607(1)	23(1)
C(2)	8546(2)	6412(2)	5600(1)	14(1)
C(4)	9145(2)	4996(2)	5583(1)	15(1)
O(1)	10658(2)	4895(2)	5944(1)	19(1)
C(5)	8187(2)	3982(2)	6140(2)	16(1)
C(6)	6525(2)	3956(2)	5887(2)	16(1)
C(7)	5286(2)	5041(2)	6089(2)	23(1)
C(8)	5348(2)	2883(2)	6220(2)	22(1)
C(9)	5802(3)	3808(3)	4887(2)	23(1)
C(10)	4442(2)	3868(2)	5587(2)	22(1)
N(2)	8395(2)	7223(2)	6398(1)	14(1)
C(11)	8835(2)	6858(2)	7362(1)	21(1)
C(3)	7757(2)	8411(2)	6126(1)	16(1)
C(12)	7434(3)	9567(2)	6771(2)	24(1)

Table S3. Bond lengths [Å] and angles [°] for (*S*)-**11**.

N(1)–C(3)	1.326(2)
N(1)–C(1)	1.368(3)
C(1)–C(2)	1.359(3)
C(1)–Br(1)	1.8780(19)
C(2)–N(2)	1.394(3)
C(2)–C(4)	1.497(3)
C(4)–O(1)	1.430(2)
C(4)–C(5)	1.530(3)
C(4)–H(4)	1.0000
O(1)–H(1O1)	0.8400
C(5)–C(6)	1.505(3)
C(5)–H(5A)	0.9900
C(5)–H(5B)	0.9900
C(6)–C(8)	1.556(3)
C(6)–C(7)	1.557(3)
C(6)–C(9)	1.560(3)
C(7)–C(10)	1.551(3)
C(7)–H(7A)	0.9900
C(7)–H(7B)	0.9900
C(8)–C(10)	1.546(3)
C(8)–H(8A)	0.9900
C(8)–H(8B)	0.9900
C(9)–C(10)	1.555(3)
C(9)–H(9A)	0.9900
C(9)–H(9B)	0.9900
C(10)–H(10)	1.0000
N(2)–C(3)	1.359(3)
N(2)–C(11)	1.465(2)
C(11)–H(11A)	0.9800
C(11)–H(11B)	0.9800
C(11)–H(11C)	0.9800
C(3)–C(12)	1.491(3)
C(12)–H(12A)	0.9800
C(12)–H(12B)	0.9800
C(12)–H(12C)	0.9800

C(3)–N(1)–C(1)	104.17(16)
C(2)–C(1)–N(1)	113.12(17)
C(2)–C(1)–Br(1)	127.20(15)
N(1)–C(1)–Br(1)	119.67(14)
C(1)–C(2)–N(2)	103.31(17)
C(1)–C(2)–C(4)	130.78(18)
N(2)–C(2)–C(4)	125.86(18)
O(1)–C(4)–C(2)	112.80(17)
O(1)–C(4)–C(5)	106.37(16)
C(2)–C(4)–C(5)	114.34(17)
O(1)–C(4)–H(4)	107.7
C(2)–C(4)–H(4)	107.7
C(5)–C(4)–H(4)	107.7
C(4)–O(1)–H(1O1)	109.5
C(6)–C(5)–C(4)	115.05(17)
C(6)–C(5)–H(5A)	108.5
C(4)–C(5)–H(5A)	108.5
C(6)–C(5)–H(5B)	108.5
C(4)–C(5)–H(5B)	108.5
H(5A)–C(5)–H(5B)	107.5
C(5)–C(6)–C(8)	125.83(18)
C(5)–C(6)–C(7)	128.67(19)
C(8)–C(6)–C(7)	87.12(16)
C(5)–C(6)–C(9)	127.83(18)
C(8)–C(6)–C(9)	86.54(16)
C(7)–C(6)–C(9)	86.91(17)
C(10)–C(7)–C(6)	74.60(15)
C(10)–C(7)–H(7A)	116.0
C(6)–C(7)–H(7A)	116.0
C(10)–C(7)–H(7B)	116.0
C(6)–C(7)–H(7B)	116.0
H(7A)–C(7)–H(7B)	113.0
C(10)–C(8)–C(6)	74.76(15)
C(10)–C(8)–H(8A)	116.0
C(6)–C(8)–H(8A)	116.0
C(10)–C(8)–H(8B)	116.0

C(6)–C(8)–H(8B)	116.0
H(8A)–C(8)–H(8B)	113.0
C(10)–C(9)–C(6)	74.38(15)
C(10)–C(9)–H(9A)	116.1
C(6)–C(9)–H(9A)	116.1
C(10)–C(9)–H(9B)	116.1
C(6)–C(9)–H(9B)	116.1
H(9A)–C(9)–H(9B)	113.1
C(8)–C(10)–C(7)	87.68(16)
C(8)–C(10)–C(9)	87.07(17)
C(7)–C(10)–C(9)	87.29(16)
C(8)–C(10)–H(10)	127.1
C(7)–C(10)–H(10)	127.1
C(9)–C(10)–H(10)	127.1
C(3)–N(2)–C(2)	107.93(17)
C(3)–N(2)–C(11)	125.91(17)
C(2)–N(2)–C(11)	126.15(17)
N(2)–C(11)–H(11A)	109.5
N(2)–C(11)–H(11B)	109.5
H(11A)–C(11)–H(11B)	109.5
N(2)–C(11)–H(11C)	109.5
H(11A)–C(11)–H(11C)	109.5
H(11B)–C(11)–H(11C)	109.5
N(1)–C(3)–N(2)	111.47(17)
N(1)–C(3)–C(12)	123.84(19)
N(2)–C(3)–C(12)	124.68(18)
C(3)–C(12)–H(12A)	109.5
C(3)–C(12)–H(12B)	109.5
H(12A)–C(12)–H(12B)	109.5
C(3)–C(12)–H(12C)	109.5
H(12A)–C(12)–H(12C)	109.5
H(12B)–C(12)–H(12C)	109.5

Symmetry transformations used to generate equivalent atoms:

Table S4. Anisotropic displacement parameters ($Å^2$x 10^3) for (*S*)-**11**. The anisotropic displacement factor exponent takes the form: $-2\square^2[h^2 a^{*2}U^{11} + ... + 2 h k a^* b^* U^{12}]$

Atom	U^{11}	U^{22}	U^{33}	U^{23}	U^{13}	U^{12}
N(1)	14(1)	17(1)	17(1)	2(1)	–1(1)	–1(1)
C(1)	15(1)	17(1)	14(1)	1(1)	1(1)	–2(1)
Br(1)	33(1)	23(1)	12(1)	1(1)	–2(1)	0(1)
C(2)	12(1)	18(1)	13(1)	0(1)	1(1)	–1(1)
C(4)	12(1)	17(1)	16(1)	0(1)	–1(1)	2(1)
O(1)	12(1)	21(1)	24(1)	7(1)	–1(1)	0(1)
C(5)	15(1)	16(1)	19(1)	2(1)	–1(1)	1(1)
C(6)	14(1)	16(1)	18(1)	0(1)	0(1)	1(1)
C(7)	16(1)	18(1)	34(1)	–2(1)	5(1)	2(1)
C(8)	18(1)	19(1)	29(1)	4(1)	–2(1)	–3(1)
C(9)	18(1)	31(1)	22(1)	0(1)	–4(1)	–2(1)
C(10)	13(1)	22(1)	32(1)	2(1)	–3(1)	0(1)
N(2)	15(1)	16(1)	13(1)	0(1)	–1(1)	0(1)
C(11)	26(1)	24(1)	14(1)	2(1)	–5(1)	1(1)
C(3)	14(1)	17(1)	17(1)	1(1)	1(1)	–1(1)
C(12)	29(1)	19(1)	24(1)	–4(1)	1(1)	2(1)

Table S5. Hydrogen coordinates (x 10^4) and isotropic displacement parameters (Å^{2}x 10^3) for (*S*)-**11**.

Atom	x	y	z	U(eq)
H(4)	9165	4691	4910	18
H(1O1)	11238	5395	5628	28
H(5A)	8613	3067	6039	20
H(5B)	8283	4192	6820	20
H(7A)	5025	5187	6761	28
H(7B)	5374	5894	5728	28
H(8A)	5489	1958	5968	26
H(8B)	5091	2895	6900	26
H(9A)	5918	4593	4460	28
H(9B)	5967	2931	4566	28
H(10)	3335	3821	5428	27
H(11A)	9081	7679	7718	31
H(11B)	9727	6266	7343	31
H(11C)	7991	6386	7671	31
H(12A)	7841	10401	6497	36
H(12B)	7916	9403	7384	36
H(12C)	6333	9657	6856	36

Table S6. Torsion angles [°] for (*S*)-**11**.

C(3)–N(1)–C(1)–C(2)	0.8(2)
C(3)–N(1)–C(1)–Br(1)	–178.12(14)
N(1)–C(1)–C(2)–N(2)	–0.5(2)
Br(1)–C(1)–C(2)–N(2)	178.32(15)
N(1)–C(1)–C(2)–C(4)	176.93(19)
Br(1)–C(1)–C(2)–C(4)	–4.2(3)
C(1)–C(2)–C(4)–O(1)	124.4(2)
N(2)–C(2)–C(4)–O(1)	–58.7(3)
C(1)–C(2)–C(4)–C(5)	–113.9(2)
N(2)–C(2)–C(4)–C(5)	63.0(3)
O(1)–C(4)–C(5)–C(6)	178.19(17)
C(2)–C(4)–C(5)–C(6)	53.0(2)
C(4)–C(5)–C(6)–C(8)	169.68(19)
C(4)–C(5)–C(6)–C(7)	–70.4(3)
C(4)–C(5)–C(6)–C(9)	51.9(3)
C(5)–C(6)–C(7)–C(10)	–178.3(2)
C(8)–C(6)–C(7)–C(10)	–43.00(15)
C(9)–C(6)–C(7)–C(10)	43.68(15)
C(5)–C(6)–C(8)–C(10)	–179.5(2)
C(7)–C(6)–C(8)–C(10)	43.14(16)
C(9)–C(6)–C(8)–C(10)	–43.94(15)
C(5)–C(6)–C(9)–C(10)	177.8(2)
C(8)–C(6)–C(9)–C(10)	43.71(15)
C(7)–C(6)–C(9)–C(10)	–43.59(15)
C(6)–C(8)–C(10)–C(7)	–43.31(14)
C(6)–C(8)–C(10)–C(9)	44.09(15)
C(6)–C(7)–C(10)–C(8)	43.33(15)
C(6)–C(7)–C(10)–C(9)	–43.84(15)
C(6)–C(9)–C(10)–C(8)	–44.04(15)
C(6)–C(9)–C(10)–C(7)	43.77(15)
C(1)–C(2)–N(2)–C(3)	0.0(2)
C(4)–C(2)–N(2)–C(3)	–177.60(19)
C(1)–C(2)–N(2)–C(11)	179.63(18)
C(4)–C(2)–N(2)–C(11)	2.0(3)
C(1)–N(1)–C(3)–N(2)	–0.8(2)

C(1)–N(1)–C(3)–C(12)	178.38(19)
C(2)–N(2)–C(3)–N(1)	0.5(2)
C(11)–N(2)–C(3)–N(1)	–179.11(18)
C(2)–N(2)–C(3)–C(12)	–178.66(19)
C(11)–N(2)–C(3)–C(12)	1.7(3)

Symmetry transformations used to generate equivalent atoms:

Table S7. Hydrogen bonds for (*S*)-**11** [Å and °].

D–H...A	d(D–H)	d(H...A)	d(D...A)	<(DHA)
O(1)–H(1O1)...N(1)#1	0.84	1.99	2.823(2)	174.9

Symmetry transformations used to generate equivalent atoms:
#1 x+1/2,–y+3/2,–z+1

4.2 (*R*)-11

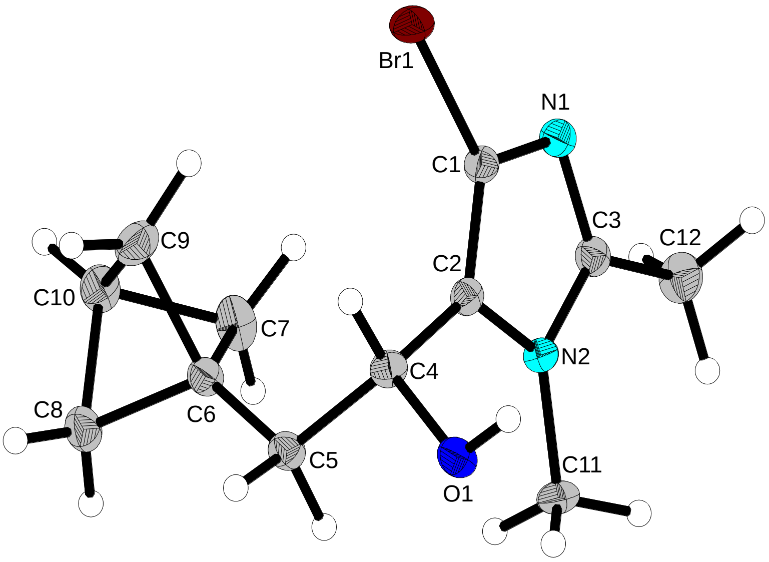

(*R*)-**11**, molecule; thermal ellipsoids are drawn on the 50% level.

Table S8. Crystal data and structure refinement for (*R*)-**11**.

Identification code	gui212	
Empirical formula	C_{12} H_{17} N_2 O Br	
Formula weight	285.18	
Temperature	100(2) K	
Wavelength	1.54184 Å	
Crystal system	Orthorhombic	
Space group	$P2_12_12_1$ (#19)	
Unit cell dimensions	a = 8.7951(2) Å	□= 90°.
	b = 9.8962(1) Å	□= 90°.
	c = 14.1699(2) Å	□ = 90°.
Volume	1233.32(4) $Å^3$	
Z	4	
Density (calculated)	1.536 Mg/m^3	
Absorption coefficient	4.391 mm^{-1}	
F(000)	584	
Crystal size	0.170 x 0.120 x 0.080 mm^3	
Theta range for data collection	5.452 to 76.801°.	
Index ranges	–10<=h<=11, –12<=k<=12, –12<=l<=17	
Reflections collected	12884	
Independent reflections	2572 [R(int) = 0.0216]	

Completeness to theta = 67.684°	100.0 %
Absorption correction	Gaussian
Max. and min. transmission	0.956 and 0.579
Refinement method	Full–matrix least–squares on F^2
Data / restraints / parameters	2572 / 0 / 152
Goodness–of–fit on F^2	1.042
Final R indices [I>2sigma(I)]	R1 = 0.0156, wR2 = 0.0402
R indices (all data)	R1 = 0.0159, wR2 = 0.0404
Absolute structure parameter	–0.029(6)
Extinction coefficient	0.0035(2)
Largest diff. peak and hole	0.202 and –0.239 e.Å^{-3}

Table S9. Atomic coordinates (x 10^4) and equivalent isotropic displacement parameters ($Å^2$x 10^3) for (*R*)-**11**. U(eq) is defined as one third of the trace of the orthogonalized U^{ij} tensor.

Atom	x	y	z	U(eq)
N(1)	2529(2)	1575(2)	4792(1)	17(1)
C(1)	2025(2)	2803(2)	5102(1)	16(1)
Br(1)	2142(1)	3229(1)	6392(1)	23(1)
C(2)	1453(2)	3587(2)	4400(1)	14(1)
C(4)	856(2)	5006(2)	4416(1)	16(1)
O(1)	–657(2)	5106(2)	4055(1)	20(1)
C(5)	1813(2)	6018(2)	3862(1)	17(1)
C(6)	3475(2)	6043(2)	4115(1)	16(1)
C(7)	4713(2)	4959(2)	3912(2)	24(1)
C(8)	4652(2)	7118(2)	3781(2)	22(1)
C(9)	4199(3)	6192(3)	5115(2)	24(1)
C(10)	5555(2)	6130(2)	4412(2)	23(1)
N(2)	1604(2)	2776(2)	3602(1)	15(1)
C(11)	1162(3)	3142(2)	2638(1)	21(1)
C(3)	2244(2)	1589(2)	3873(1)	17(1)
C(12)	2562(3)	431(2)	3229(2)	25(1)

Table S10. Bond lengths [Å] and angles [°] for (*R*)-**11**.

N(1)–C(3)	1.326(2)
N(1)–C(1)	1.366(2)
C(1)–C(2)	1.359(3)
C(1)–Br(1)	1.8790(18)
C(2)–N(2)	1.393(2)
C(2)–C(4)	1.498(3)
C(4)–O(1)	1.429(2)
C(4)–C(5)	1.526(3)
C(4)–H(4)	1.0000
O(1)–H(1O1)	0.78(4)
C(5)–C(6)	1.505(3)
C(5)–H(5A)	0.9900
C(5)–H(5B)	0.9900
C(6)–C(7)	1.555(3)
C(6)–C(8)	1.559(3)
C(6)–C(9)	1.561(3)
C(7)–C(10)	1.547(3)
C(7)–H(7A)	0.9900
C(7)–H(7B)	0.9900
C(8)–C(10)	1.545(3)
C(8)–H(8A)	0.9900
C(8)–H(8B)	0.9900
C(9)–C(10)	1.556(3)
C(9)–H(9A)	0.9900
C(9)–H(9B)	0.9900
C(10)–H(10)	1.0000
N(2)–C(3)	1.358(3)
N(2)–C(11)	1.465(2)
C(11)–H(11A)	0.9800
C(11)–H(11B)	0.9800
C(11)–H(11C)	0.9800
C(3)–C(12)	1.491(3)
C(12)–H(12A)	0.9800
C(12)–H(12B)	0.9800
C(12)–H(12C)	0.9800

C(3)–N(1)–C(1)	104.18(16)
C(2)–C(1)–N(1)	113.14(16)
C(2)–C(1)–Br(1)	127.18(15)
N(1)–C(1)–Br(1)	119.67(14)
C(1)–C(2)–N(2)	103.30(16)
C(1)–C(2)–C(4)	130.84(17)
N(2)–C(2)–C(4)	125.80(17)
O(1)–C(4)–C(2)	112.68(16)
O(1)–C(4)–C(5)	106.50(15)
C(2)–C(4)–C(5)	114.47(17)
O(1)–C(4)–H(4)	107.6
C(2)–C(4)–H(4)	107.6
C(5)–C(4)–H(4)	107.6
C(4)–O(1)–H(1O1)	111(2)
C(6)–C(5)–C(4)	115.06(16)
C(6)–C(5)–H(5A)	108.5
C(4)–C(5)–H(5A)	108.5
C(6)–C(5)–H(5B)	108.5
C(4)–C(5)–H(5B)	108.5
H(5A)–C(5)–H(5B)	107.5
C(5)–C(6)–C(7)	128.66(18)
C(5)–C(6)–C(8)	125.73(18)
C(7)–C(6)–C(8)	87.09(16)
C(5)–C(6)–C(9)	127.90(17)
C(7)–C(6)–C(9)	86.96(17)
C(8)–C(6)–C(9)	86.58(16)
C(10)–C(7)–C(6)	74.58(15)
C(10)–C(7)–H(7A)	116.0
C(6)–C(7)–H(7A)	116.0
C(10)–C(7)–H(7B)	116.0
C(6)–C(7)–H(7B)	116.0
H(7A)–C(7)–H(7B)	113.0
C(10)–C(8)–C(6)	74.55(15)
C(10)–C(8)–H(8A)	116.0
C(6)–C(8)–H(8A)	116.0
C(10)–C(8)–H(8B)	116.0

C(6)–C(8)–H(8B)	116.0
H(8A)–C(8)–H(8B)	113.0
C(10)–C(9)–C(6)	74.17(15)
C(10)–C(9)–H(9A)	116.1
C(6)–C(9)–H(9A)	116.1
C(10)–C(9)–H(9B)	116.1
C(6)–C(9)–H(9B)	116.1
H(9A)–C(9)–H(9B)	113.1
C(8)–C(10)–C(7)	87.90(16)
C(8)–C(10)–C(9)	87.25(17)
C(7)–C(10)–C(9)	87.45(16)
C(8)–C(10)–H(10)	127.0
C(7)–C(10)–H(10)	127.0
C(9)–C(10)–H(10)	127.0
C(3)–N(2)–C(2)	107.94(16)
C(3)–N(2)–C(11)	125.96(17)
C(2)–N(2)–C(11)	126.10(17)
N(2)–C(11)–H(11A)	109.5
N(2)–C(11)–H(11B)	109.5
H(11A)–C(11)–H(11B)	109.5
N(2)–C(11)–H(11C)	109.5
H(11A)–C(11)–H(11C)	109.5
H(11B)–C(11)–H(11C)	109.5
N(1)–C(3)–N(2)	111.43(17)
N(1)–C(3)–C(12)	123.87(19)
N(2)–C(3)–C(12)	124.69(17)
C(3)–C(12)–H(12A)	109.5
C(3)–C(12)–H(12B)	109.5
H(12A)–C(12)–H(12B)	109.5
C(3)–C(12)–H(12C)	109.5
H(12A)–C(12)–H(12C)	109.5
H(12B)–C(12)–H(12C)	109.5

Symmetry transformations used to generate equivalent atoms:

Table S11. Anisotropic displacement parameters ($Å^2$x 10^3) for (*R*)-**11**. The anisotropic displacement factor exponent takes the form: $-2\square^2[h^2 a^{*2}U^{11} + ... + 2 h k a^* b^* U^{12}]$

Atom	U^{11}	U^{22}	U^{33}	U^{23}	U^{13}	U^{12}
N(1)	15(1)	18(1)	17(1)	2(1)	0(1)	0(1)
C(1)	16(1)	17(1)	14(1)	0(1)	1(1)	–2(1)
Br(1)	34(1)	24(1)	13(1)	1(1)	–2(1)	0(1)
C(2)	12(1)	17(1)	14(1)	0(1)	1(1)	–2(1)
C(4)	13(1)	18(1)	17(1)	0(1)	–1(1)	2(1)
O(1)	13(1)	21(1)	24(1)	7(1)	0(1)	–1(1)
C(5)	15(1)	16(1)	18(1)	2(1)	0(1)	1(1)
C(6)	16(1)	16(1)	18(1)	2(1)	0(1)	0(1)
C(7)	16(1)	20(1)	35(1)	–2(1)	5(1)	2(1)
C(8)	18(1)	19(1)	29(1)	4(1)	–2(1)	–3(1)
C(9)	19(1)	32(1)	22(1)	0(1)	–5(1)	–3(1)
C(10)	14(1)	22(1)	32(1)	3(1)	–3(1)	0(1)
N(2)	16(1)	16(1)	13(1)	0(1)	–1(1)	0(1)
C(11)	27(1)	23(1)	13(1)	2(1)	–5(1)	0(1)
C(3)	15(1)	17(1)	18(1)	1(1)	1(1)	–1(1)
C(12)	28(1)	21(1)	24(1)	–6(1)	1(1)	3(1)

Table S12. Hydrogen coordinates (x 10^4) and isotropic displacement parameters (Å^{2}x 10^3) for (*R*)-**11**.

Atom	x	y	z	U(eq)
H(4)	835	5310	5089	19
H(1O1)	–1210(40)	4640(30)	4340(20)	42(9)
H(5A)	1718	5809	3181	20
H(5B)	1388	6933	3963	20
H(7A)	4627	4106	4274	28
H(7B)	4972	4814	3240	28
H(8A)	4909	7106	3100	26
H(8B)	4514	8043	4033	26
H(9A)	4036	7069	5436	29
H(9B)	4084	5407	5543	29
H(10)	6662	6175	4569	27
H(11A)	272	3737	2659	32
H(11B)	911	2321	2284	32
H(11C)	2007	3611	2329	32
H(12A)	2060	586	2621	37
H(12B)	2174	–404	3512	37
H(12C)	3662	352	3131	37

Table S13. Torsion angles [°] for (*R*)-**11**.

C(3)–N(1)–C(1)–C(2)	–0.8(2)
C(3)–N(1)–C(1)–Br(1)	178.17(14)
N(1)–C(1)–C(2)–N(2)	0.6(2)
Br(1)–C(1)–C(2)–N(2)	–178.32(15)
N(1)–C(1)–C(2)–C(4)	–176.8(2)
Br(1)–C(1)–C(2)–C(4)	4.4(3)
C(1)–C(2)–C(4)–O(1)	–124.4(2)
N(2)–C(2)–C(4)–O(1)	58.8(3)
C(1)–C(2)–C(4)–C(5)	113.7(2)
N(2)–C(2)–C(4)–C(5)	–63.1(3)
O(1)–C(4)–C(5)–C(6)	–178.18(16)
C(2)–C(4)–C(5)–C(6)	–53.0(2)
C(4)–C(5)–C(6)–C(7)	70.5(3)
C(4)–C(5)–C(6)–C(8)	–169.81(18)
C(4)–C(5)–C(6)–C(9)	–52.0(3)
C(5)–C(6)–C(7)–C(10)	178.1(2)
C(8)–C(6)–C(7)–C(10)	43.00(15)
C(9)–C(6)–C(7)–C(10)	–43.73(15)
C(5)–C(6)–C(8)–C(10)	179.7(2)
C(7)–C(6)–C(8)–C(10)	–43.07(15)
C(9)–C(6)–C(8)–C(10)	44.05(15)
C(5)–C(6)–C(9)–C(10)	–177.8(2)
C(7)–C(6)–C(9)–C(10)	43.51(15)
C(8)–C(6)–C(9)–C(10)	–43.75(15)
C(6)–C(8)–C(10)–C(7)	43.35(14)
C(6)–C(8)–C(10)–C(9)	–44.20(14)
C(6)–C(7)–C(10)–C(8)	–43.45(15)
C(6)–C(7)–C(10)–C(9)	43.89(14)
C(6)–C(9)–C(10)–C(8)	44.22(14)
C(6)–C(9)–C(10)–C(7)	–43.80(15)
C(1)–C(2)–N(2)–C(3)	–0.1(2)
C(4)–C(2)–N(2)–C(3)	177.40(19)
C(1)–C(2)–N(2)–C(11)	–179.69(18)
C(4)–C(2)–N(2)–C(11)	–2.2(3)
C(1)–N(1)–C(3)–N(2)	0.7(2)

C(1)–N(1)–C(3)–C(12)	–178.1(2)
C(2)–N(2)–C(3)–N(1)	–0.4(2)
C(11)–N(2)–C(3)–N(1)	179.19(18)
C(2)–N(2)–C(3)–C(12)	178.43(19)
C(11)–N(2)–C(3)–C(12)	–2.0(3)

Symmetry transformations used to generate equivalent atoms:

Table S14. Hydrogen bonds for (*R*)-**11** [Å and °].

D–H...A	d(D–H)	d(H...A)	d(D...A)	<(DHA)
O(1)–H(1O1)...N(1)#1	0.78(4)	2.05(4)	2.824(2)	173(3)

Symmetry transformations used to generate equivalent atoms:
#1 x–1/2,–y+1/2,–z+1

5. Biological Data

			6a			
			IC50	I max	Max Inhibition (%)	p val
LPS-triggered	NFkB-driven Luciferase activity		1pM	100nM	46 ± 11	*
	Cytokine release	IL-4	100fM	10pM	70 ± 7	***
		MCP-1			48 ± 7	**
		MIP-1α			42 ± 7	**
		TNF-α			41 ± 7	**
		IL-1β			39 ± 17	ns
		IL-13			30 ± 22	ns
		GM-CSF			26 ± 14	*
		IL-6			24 ± 12	*

Table S15. Pharmacodynamic (PD) analysis of the effects of BCP-sLXms 6a on NFκB activity. THP-1 LUCIA monocytes were treated with appropriate controls or BCP-sLXms, as described in Figure legend 2. The table summarises the effects of **6a** on LPS-induced NF-κB activity in monocytes by measuring maximal inhibitory concentration (Imax), half-maximal inhibitory concentration (IC_{50}) and maximal activity (%), as a measure of efficacy and potency relative to LPS-induced response (set at 100%). Statistical analysis was carried out by using Student's unpaired 2-tailed T-test of tested compound *vs* LPS (ns p = not significant; * $p < 0.05$; ** $p < 0.01$; *** $p < 0.001$).

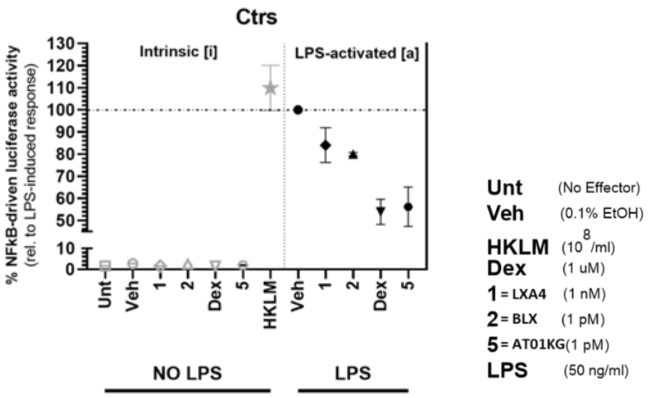

Figure S7. **NF-κB activity assay experimental controls.** % relative luminescence unit (RLU) indicating NF-κB-driven luciferase activity for the various controls used in presence and absence of LPS challenge (as displayed in figure legend).

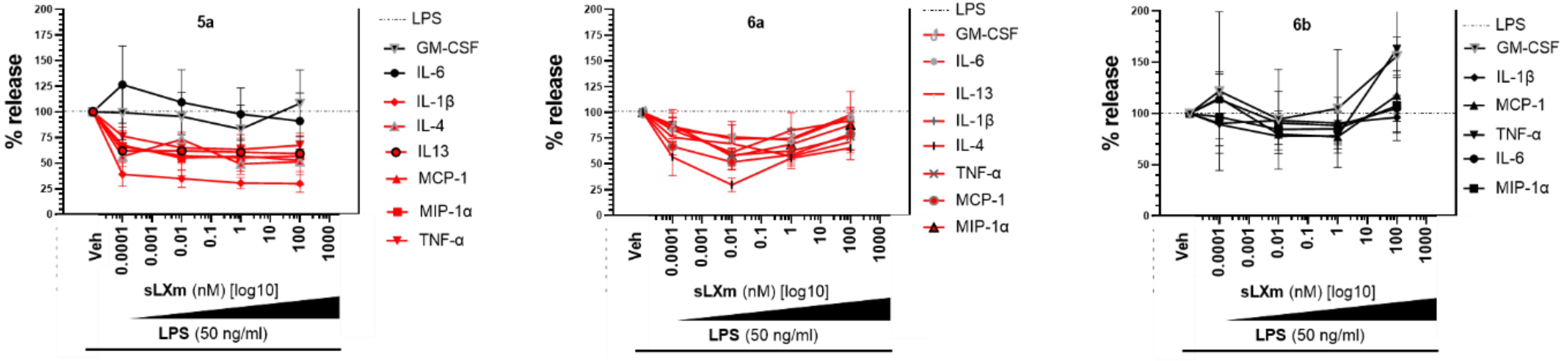

Figure S8. Complete Th1 pro-inflammatory cytokines analysis. 1x10^5 THP-1 LUCIA monocytes were pre-treated for 30 mins at indicated concentrations with **5a**, **6a** or **6b**. After 24 h from the subsequent stimulation with LPS, supernatants were collected and a panel of 8 pro-inflammatory cytokine levels were measured (MIP-1a, MCP-1, TNF-a, IL-1b, IL-6, IL-13, GM-CSF and IL-4). Dose-response curves show downregulated (red) or unmodified (black) cytokines. Statistical analysis was carried out by using Student's unpaired 2-tailed T-test of tested compound *vs* LPS.

Cyto-/chemo-kine ABUNDANCY in THP-1 Monocyte Releasate

MIP-1α

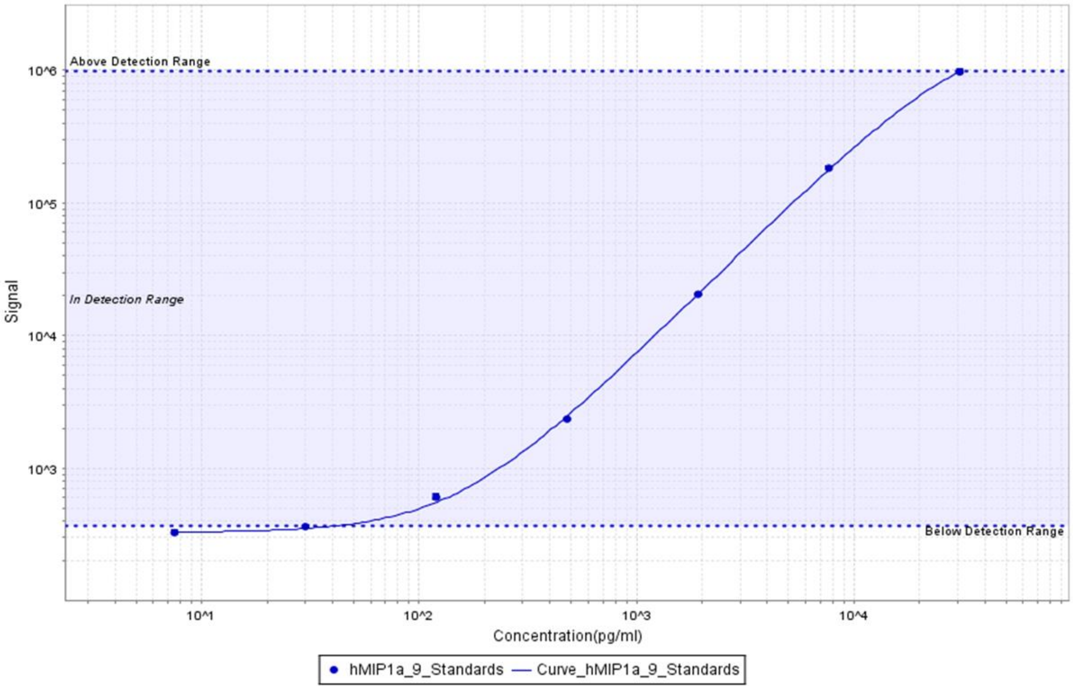

MCP-1

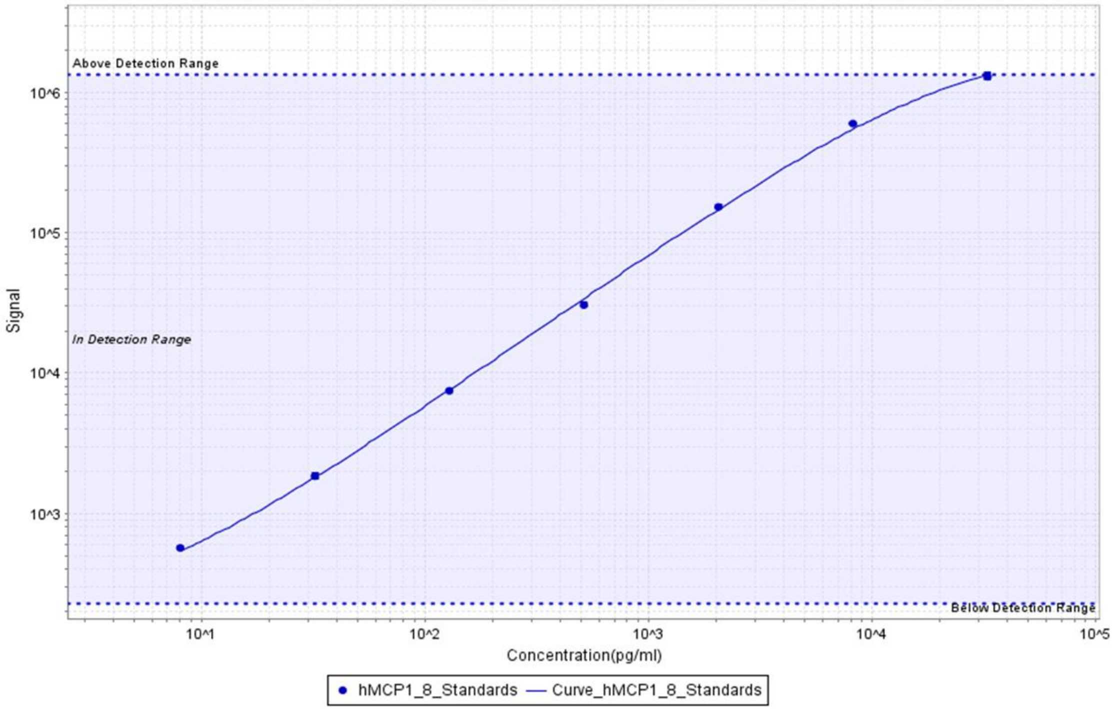

TNF-α

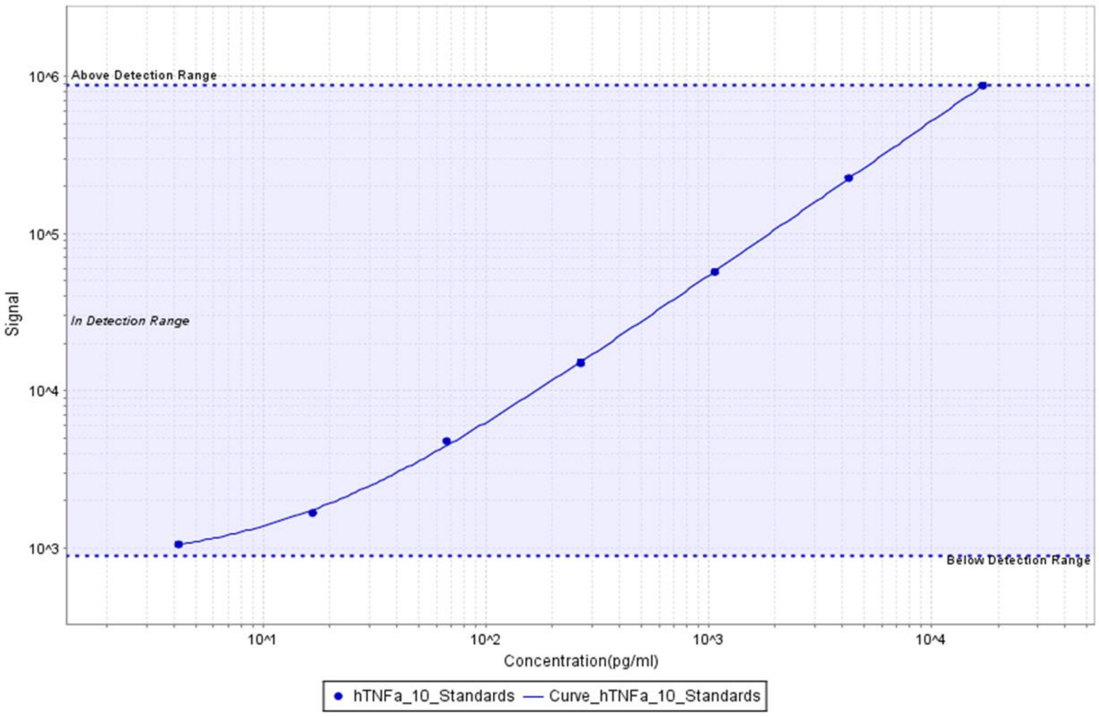
10^6
10^5
10^4
10^3
Above Detection Range
In Detection Range
Below Detection Range
Signal
10^1
10^2
10^3
10^4
Concentration(pg/ml)
hTNFa_10_Standards
Curve_hTNFa_10_Standards

IL-1β

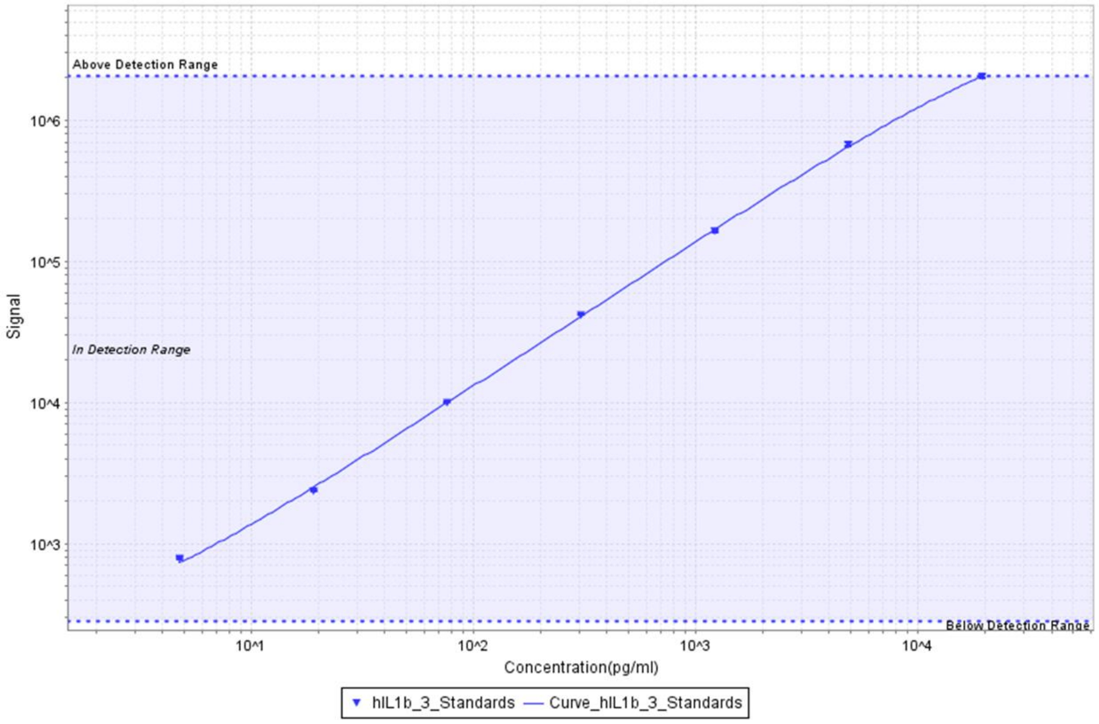
10^6
10^5
10^4
10^3
Above Detection Range
In Detection Range
Below Detection Range
Signal
10^1
10^2
10^3
10^4
Concentration(pg/ml)
hIL1b_3_Standards
Curve_hIL1b_3_Standards

IL-6

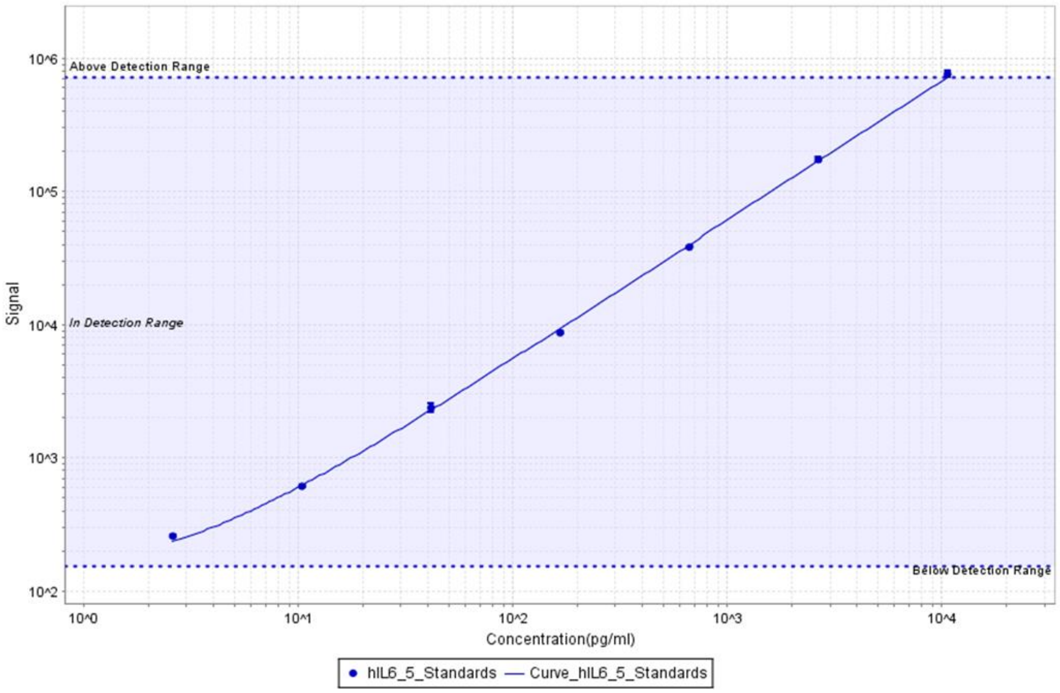

10^6
10^5
10^4
10^3
10^2
Above Detection Range
In Detection Range
Below Detection Range
Signal
10^0
10^1
10^2
10^3
10^4
Concentration(pg/ml)
hIL6_5_Standards
Curve_hIL6_5_Standards

IL-13

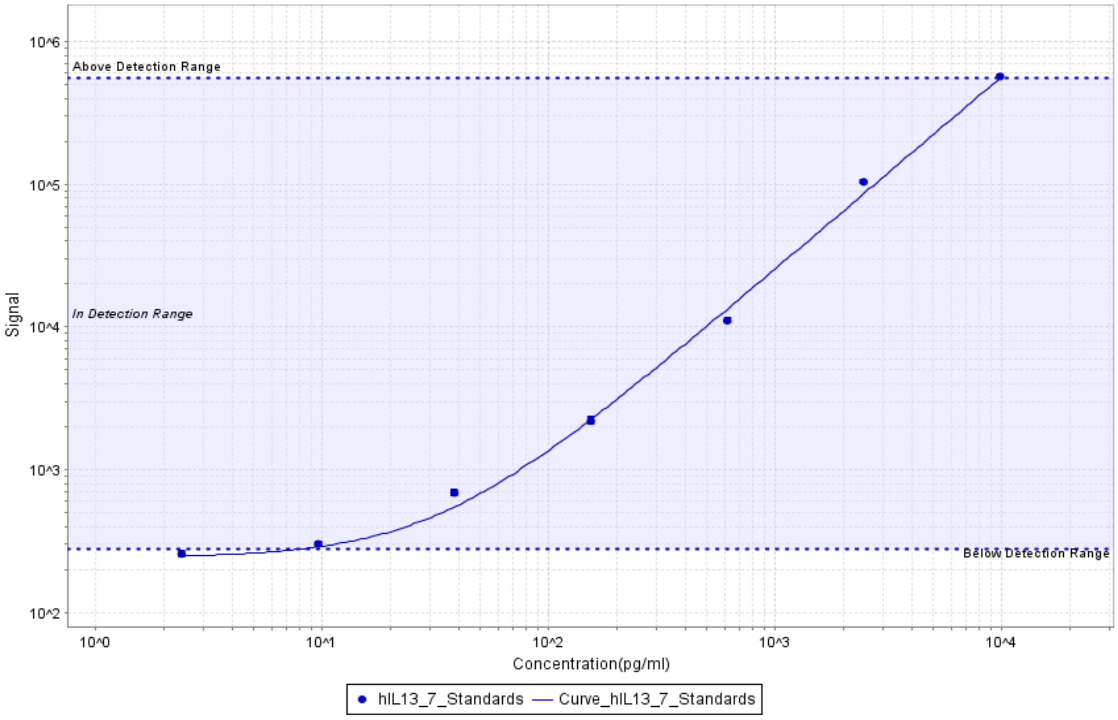

10^6
10^5
10^4
10^3
10^2
Above Detection Range
In Detection Range
Below Detection Range
Signal
10^0
10^1
10^2
10^3
10^4
Concentration(pg/ml)
hIL13_7_Standards
Curve_hIL13_7_Standards

GM-CSF

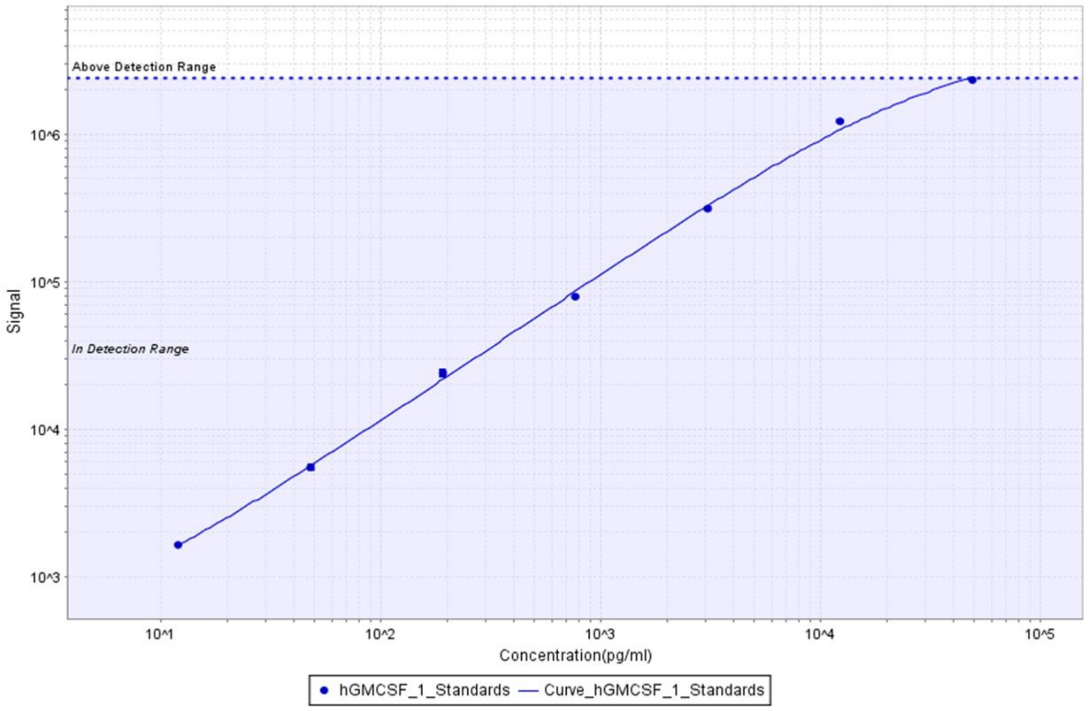

IL-4

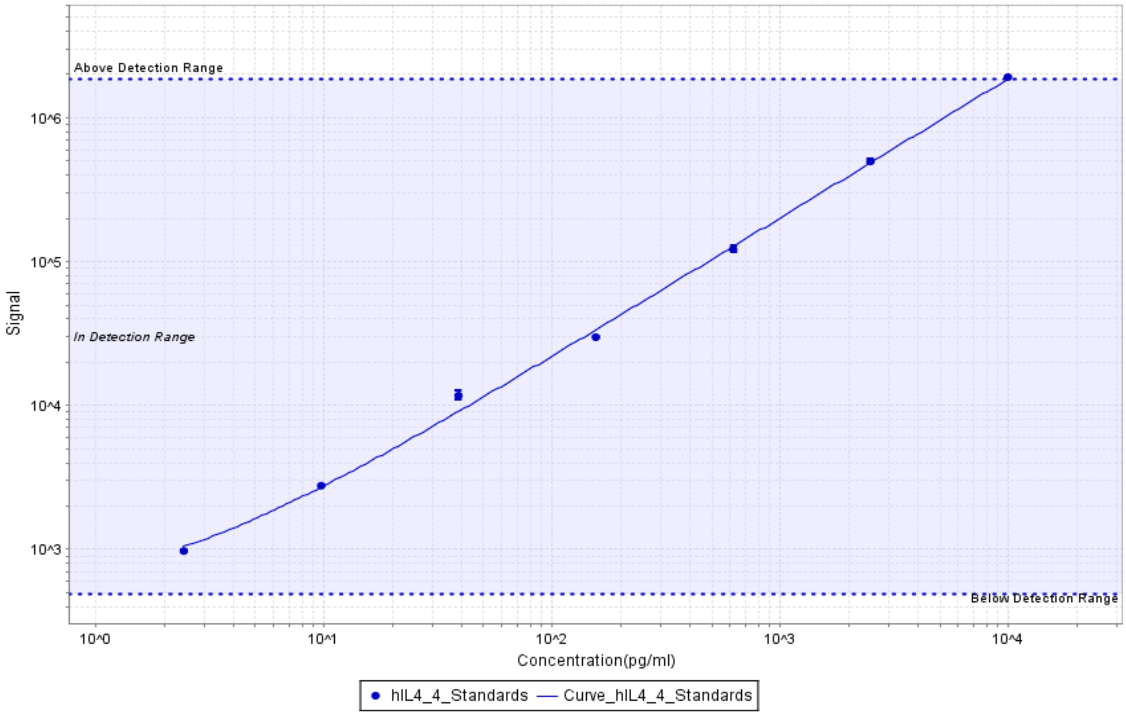

Figure S9 - Standard curves for electrochemiluminescence detection of Th1 pro-inflammatory cytokines. Using a multiplex electrochemiluminescence technology, MIP-1a, MCP-1, TNF-a, IL-1b, IL-6, IL-13, GM-CSF and IL-4 levels were all detected within the standard range, therefore, no dilution of sample was required prior to perform the assay.

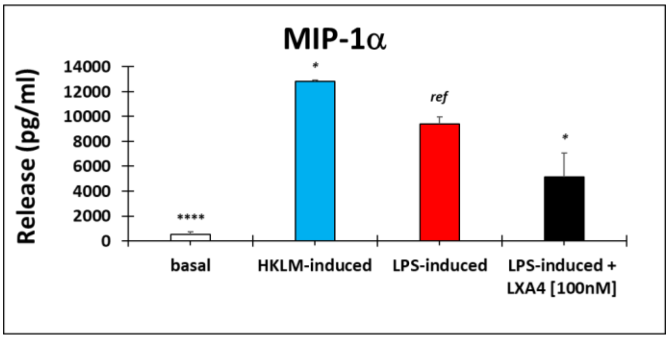
MIP-1α
Release (pg/ml)
14000
12000
10000
8000
6000
4000
2000
0
****
*
ref
*
basal
HKLM-induced
LPS-induced
LPS-induced + LXA4 [100nM]

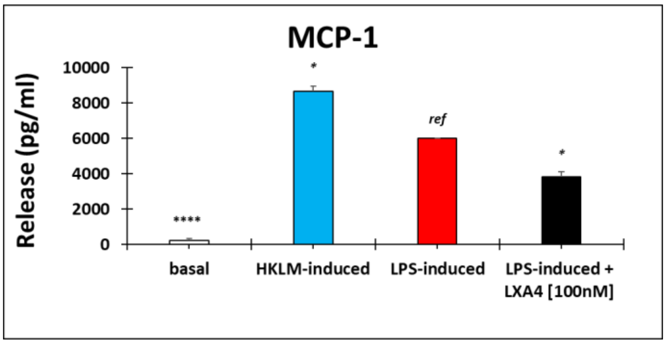
MCP-1
Release (pg/ml)
10000
8000
6000
4000
2000
0
****
*
ref
*
basal
HKLM-induced
LPS-induced
LPS-induced + LXA4 [100nM]

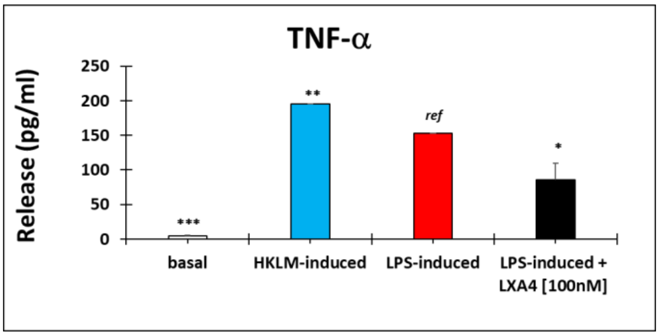
TNF-α
Release (pg/ml)
250
200
150
100
50
0
***
**
ref
*
basal
HKLM-induced
LPS-induced
LPS-induced + LXA4 [100nM]

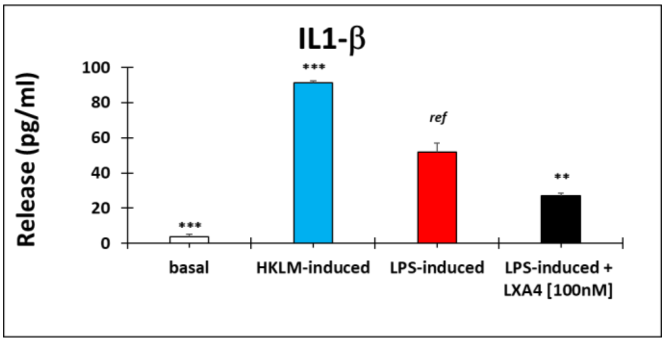
IL1-β
Release (pg/ml)
100
80
60
40
20
0
***
***
ref
**
basal
HKLM-induced
LPS-induced
LPS-induced + LXA4 [100nM]

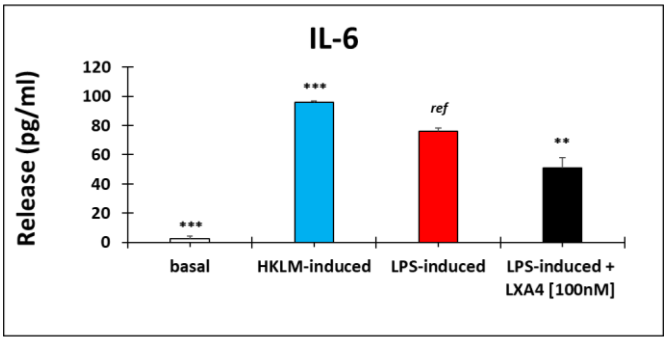
IL-6
Release (pg/ml)
120
100
80
60
40
20
0
***
***
ref
**
basal
HKLM-induced
LPS-induced
LPS-induced + LXA4 [100nM]

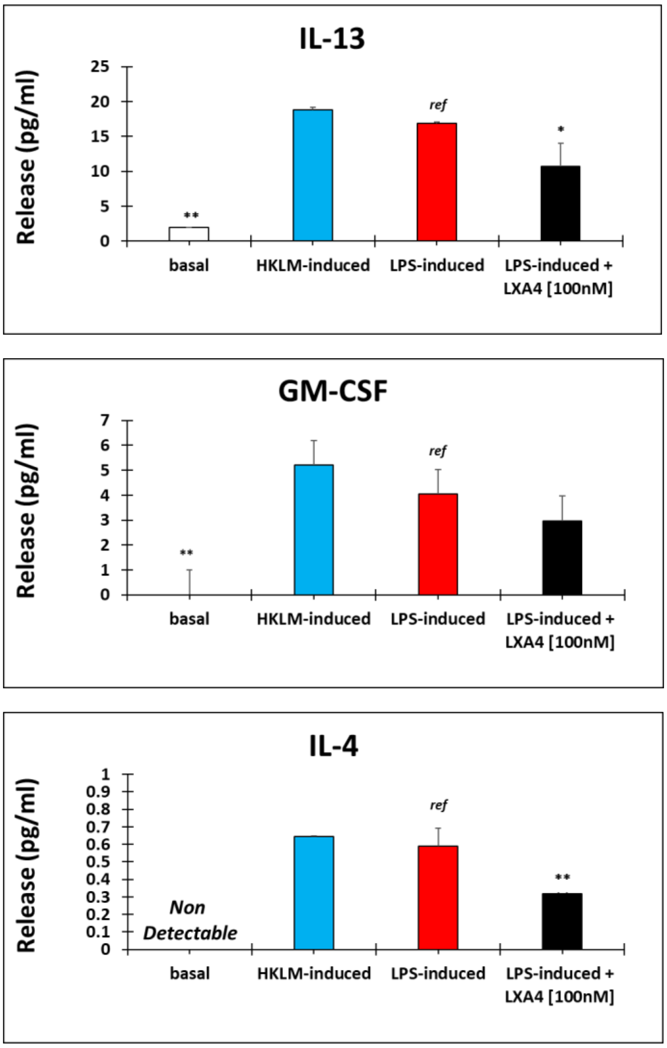

Figure S10 - Control for electrochemiluminescence detection of Th1 pro-inflammatory cytokines. Using a multiplex electrochemiluminescence technology, MIP-1a, MCP-1, TNF-a, IL-1b, IL-6, IL-13, GM-CSF and IL-4 levels were measured as pg/mL after treatment with vehicle (0.01% EtOH), HKLM (10^8/ml), 50ng/mL LPs or LXA4 (100nM).

6. References

(1) de Gaetano, M.; Tighe, C.; Gahan, K.; Zanetti, A.; Chen, J.; Newson, J.; Cacace, A.; Marai, M.; Gaffney, A.; Brennan, E.; Kantharidis, P.; Cooper, M. E.; Leroy, X.; Perretti, M.; Gilroy, D.; Godson, C.; Guiry, P. J. Asymmetric Synthesis and Biological Screening of Quinoxaline-Containing Synthetic Lipoxin A4 Mimetics (QNX-SLXms). *J. Med. Chem.* **2021**, *64* (13), 9193–9216. https://doi.org/10.1021/acs.jmedchem.1c00403.

(2) Gianatassio, R.; Lopchuk, J. M.; Wang, J.; Pan, C.-M.; Malins, L. R.; Prieto, L.; Brandt, T. A.; Collins, M. R.; Gallego, G. M.; Sach, N. W.; Spangler, J. E.; Zhu, H.; Zhu, J.; Baran, P. S. Strain-Release Amination. *Science* **2016**, *351* (6270), 241–246. https://doi.org/10.1126/science.aad6252.

(3) Wang, Z.; Wang, L.; Wang, Z.; Li, P.; Zhang, Y. A Practical Synthesis of α-Bromo/Iodo/Chloroketones from Olefins under Visible-Light Irradiation Conditions. *Chin. Chem. Lett.* **2021**, *32* (1), 429–432. https://doi.org/10.1016/j.cclet.2020.02.022.

(4) de Gaetano, M.; Butler, E.; Gahan, K.; Zanetti, A.; Marai, M.; Chen, J.; Cacace, A.; Hams, E.; Maingot, C.; McLoughlin, A.; Brennan, E.; Leroy, X.; Loscher, C. E.; Fallon, P.; Perretti, M.; Godson, C.; Guiry, P. J. Asymmetric Synthesis and Biological Evaluation of Imidazole- and Oxazole-Containing Synthetic Lipoxin A4 Mimetics (SLXms). *Eur. J. Med. Chem.* **2019**, *162*, 80–108. https://doi.org/10.1016/j.ejmech.2018.10.049.

7. NMR Spectra

(3aR,7aS)-2,2-dimethyltetrahydro-4H-[1,3]dioxolo[4,5-c]pyran-6-ol (S1)

^{1}H-NMR (400 MHz, $CDCl_3$):

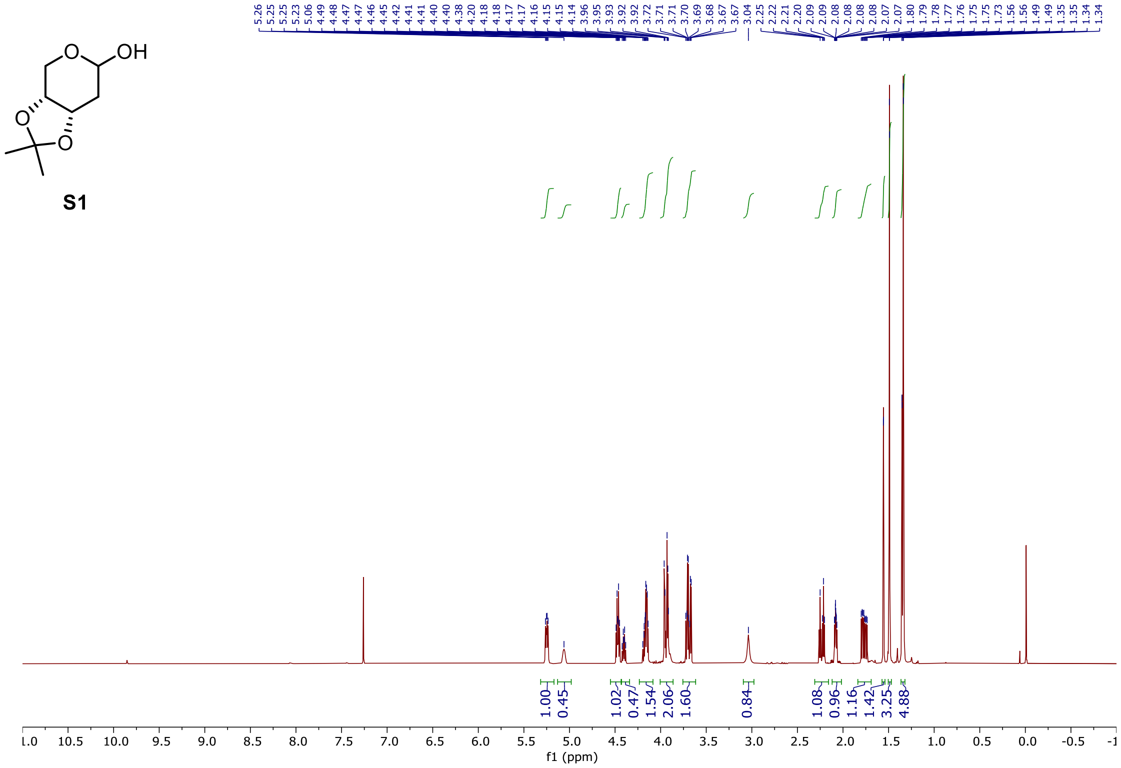

^{13}C-NMR (101 MHz, $CDCl_3$)**:**

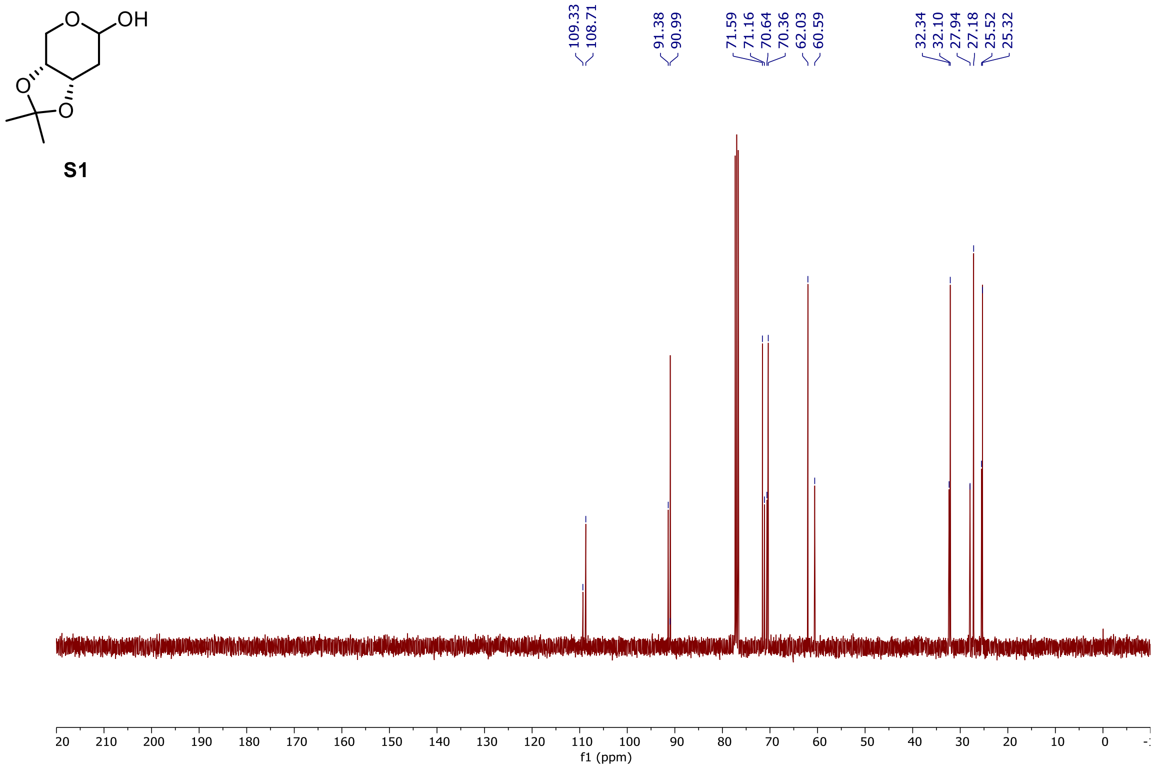

methyl-4-((4S,5R)-5-(hydroxymethyl)-2,2-dimethyl-1,3-dioxolan-4-yl)but-2-enoate (S2)

^{1}H-NMR (400 MHz, $CDCl_3$)**:**

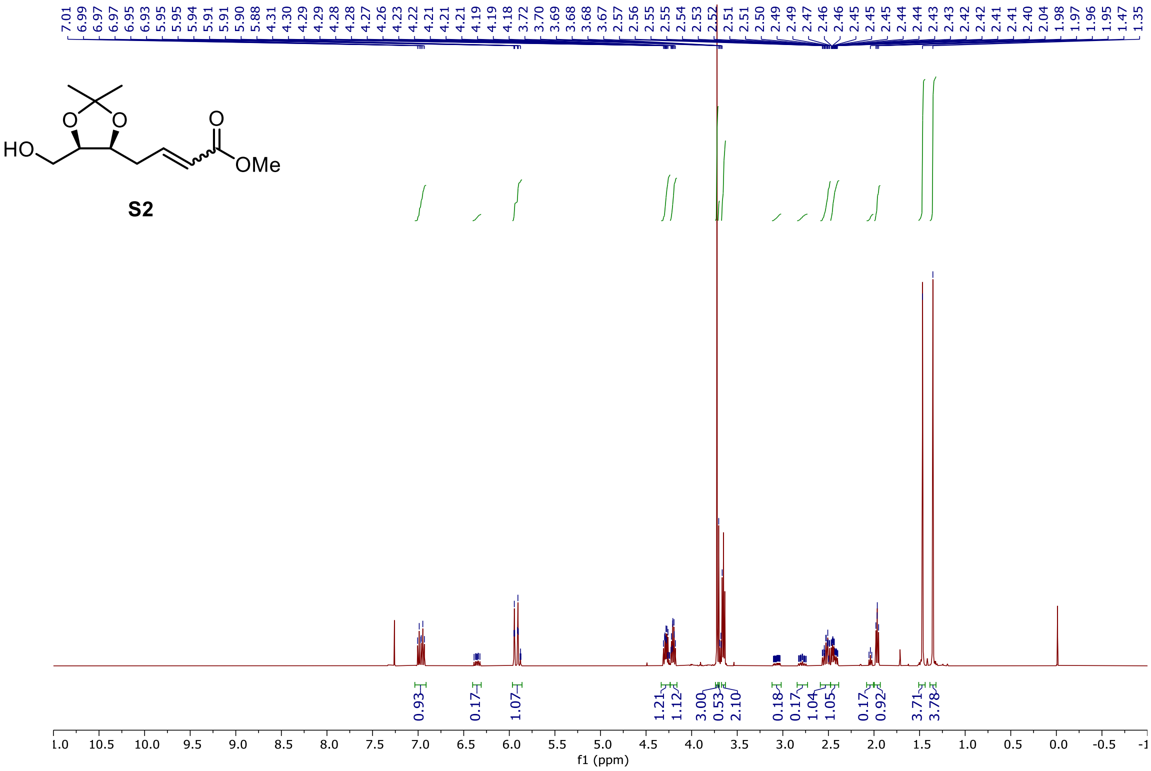

^{13}C-NMR (101 MHz, $CDCl_3$)**:**

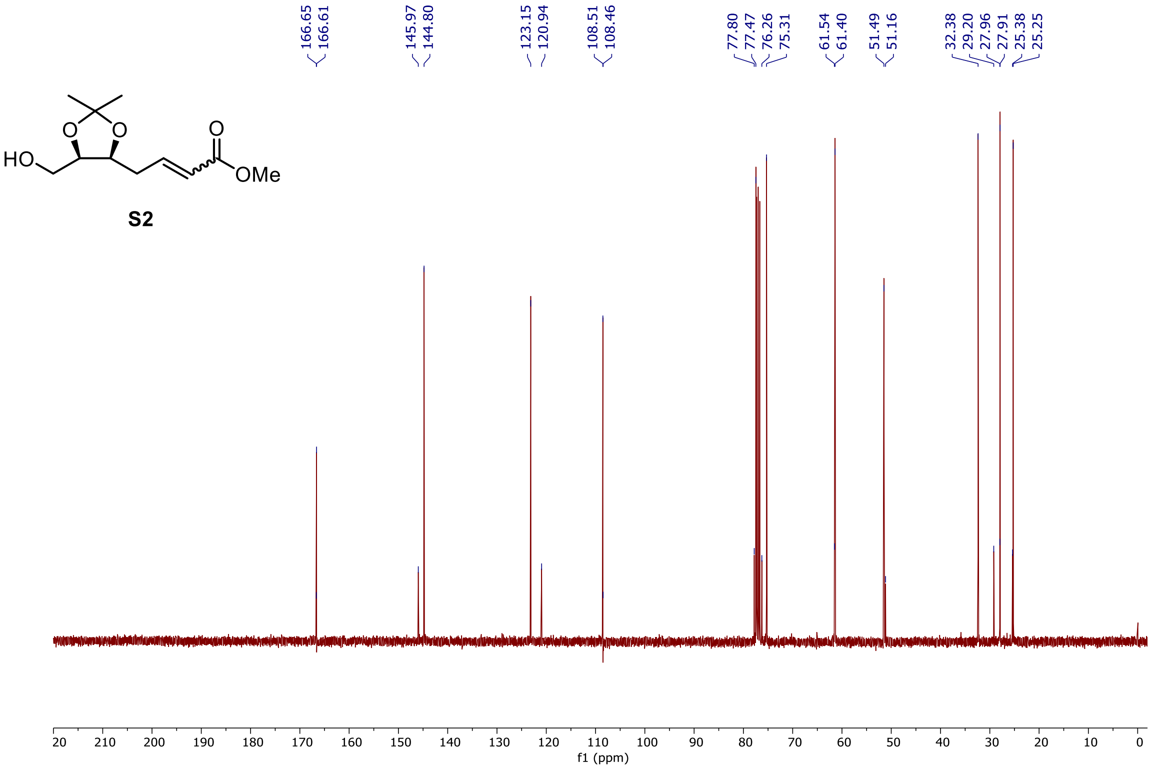

methyl 4-((4S,5R)-5-(hydroxymethyl)-2,2-dimethyl-1,3-dioxolan-4-yl)butanoate (S3)

^{1}H-NMR (400 MHz, $CDCl_3$)**:**

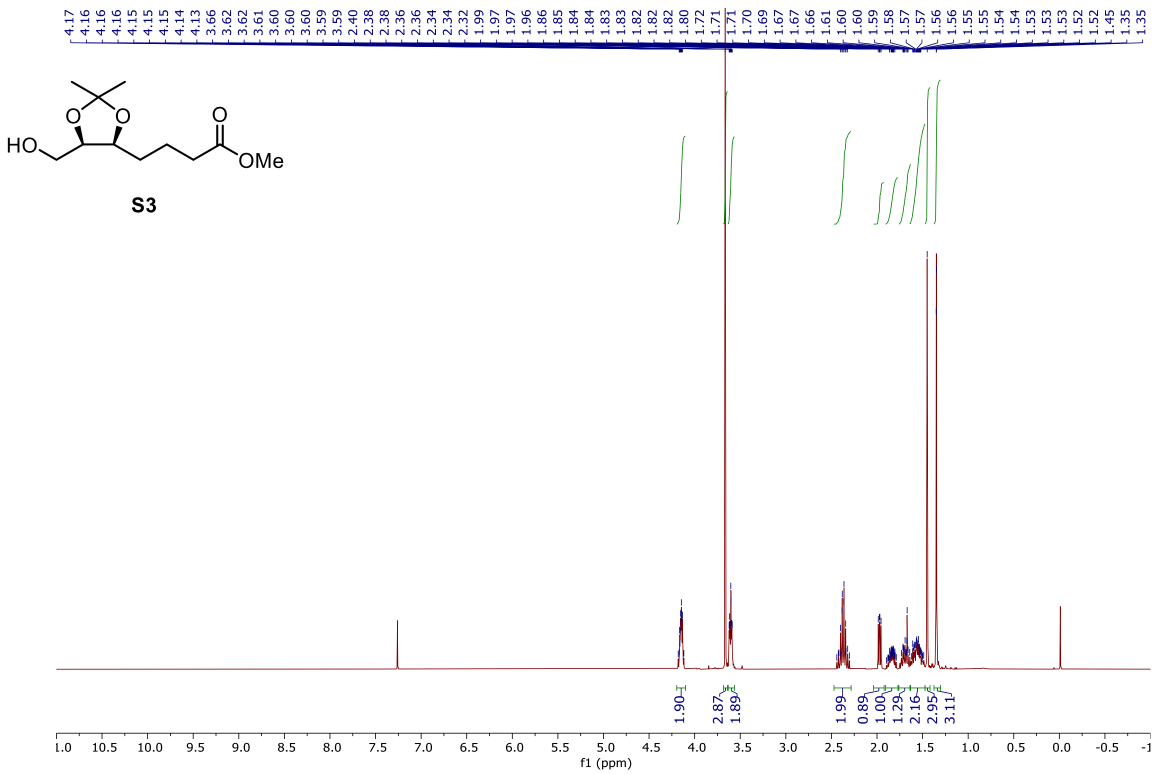

^{13}C-NMR (101 MHz, $CDCl_3$)**:**

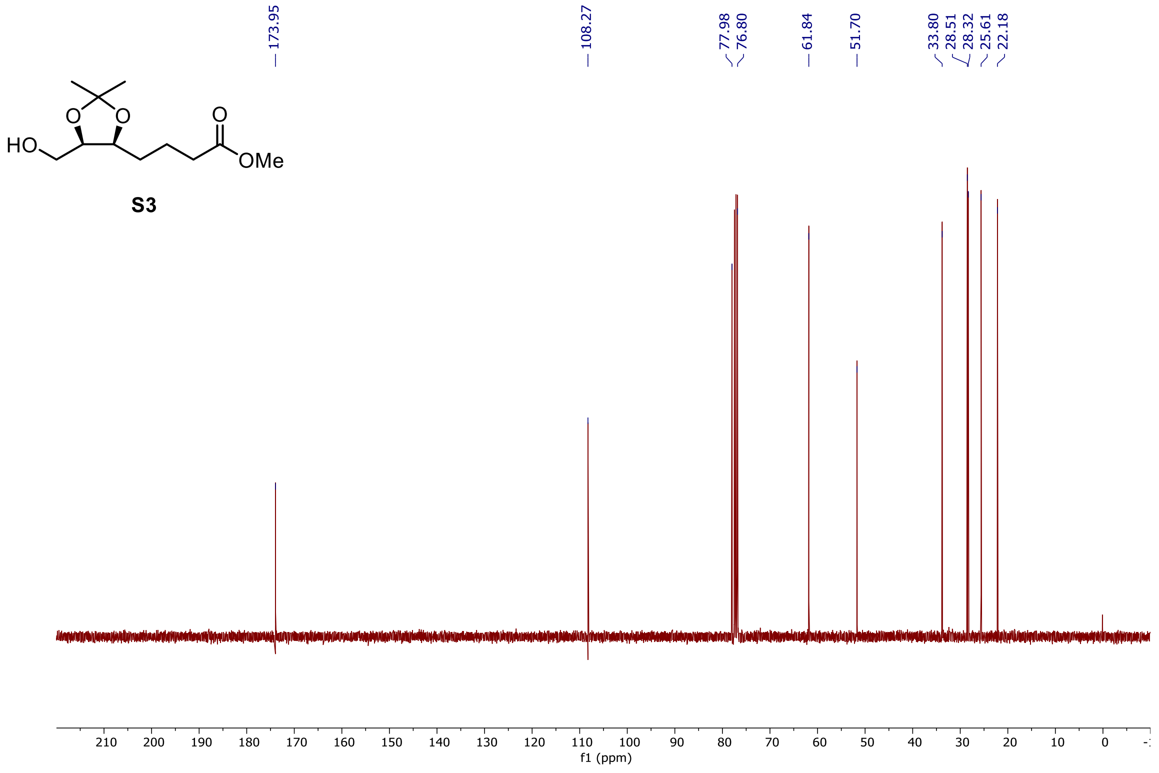

methyl 4-((4S,5S)-5-formyl-2,2-dimethyl-1,3-dioxolan-4-yl)butanoate (S4)

^{1}H-NMR (400 MHz, $CDCl_3$)**:**

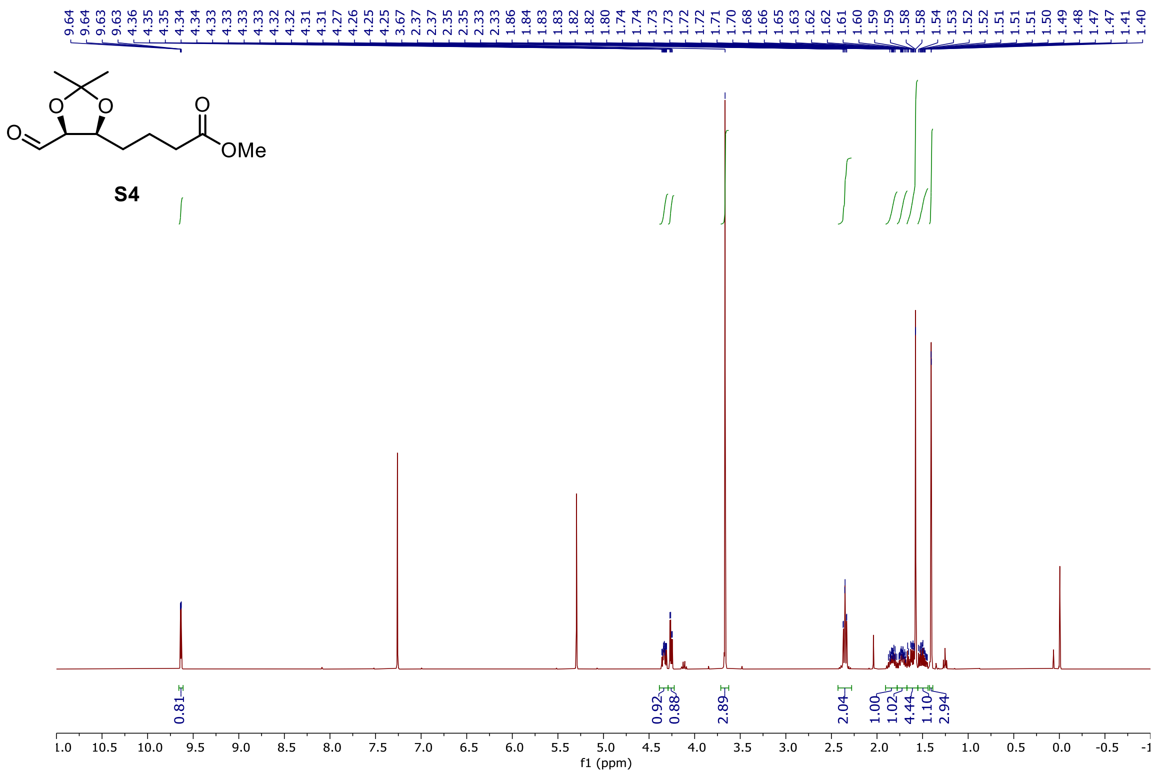

^{13}C-NMR (101 MHz, $CDCl_3$)**:**

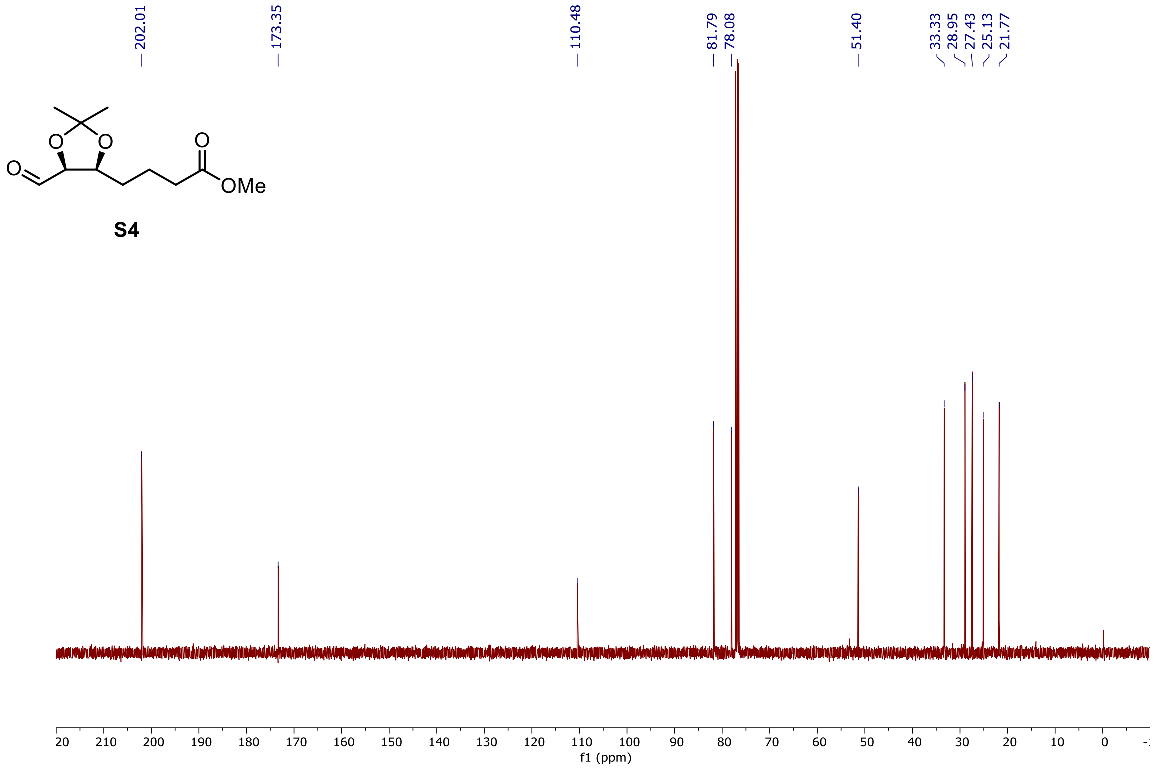

2-(dichloromethyl)-4,4,5,5-tetramethyl-1,3,2-dioxaborolane (S5)

^{1}H-NMR (400 MHz, $CDCl_3$)**:**

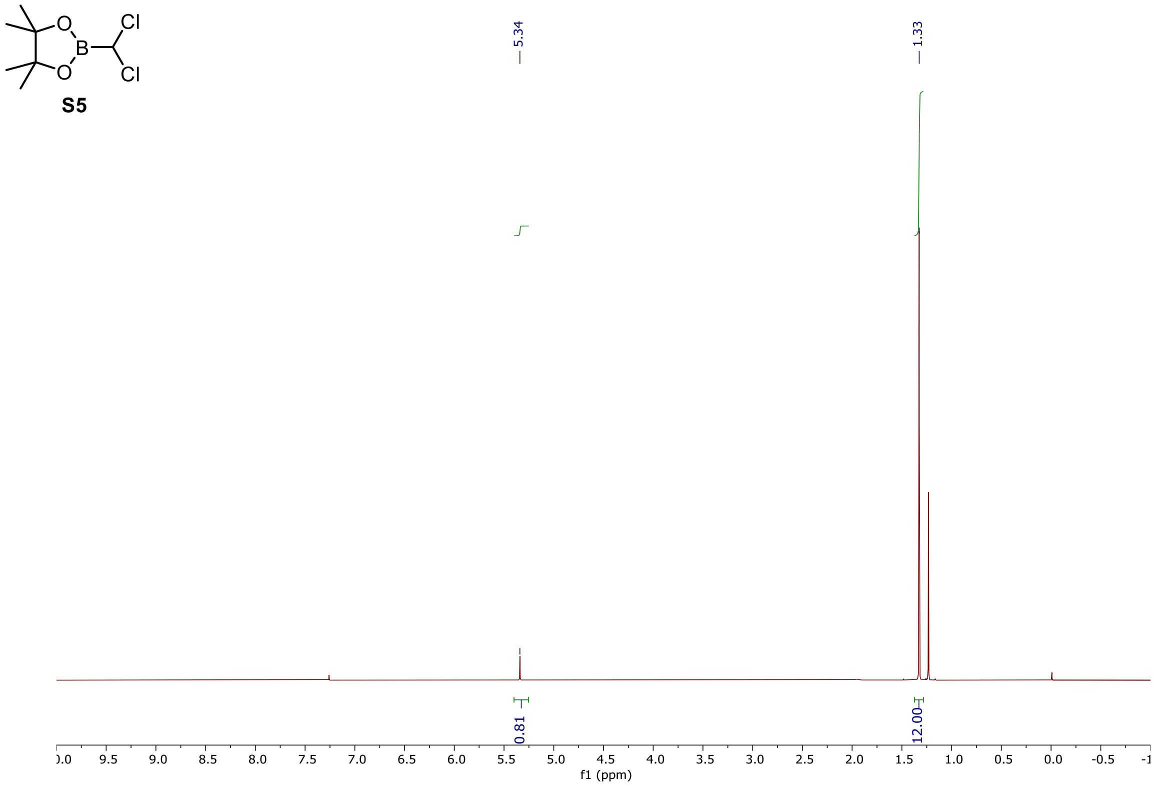

^{13}C-NMR (101 MHz, $CDCl_3$)**:**

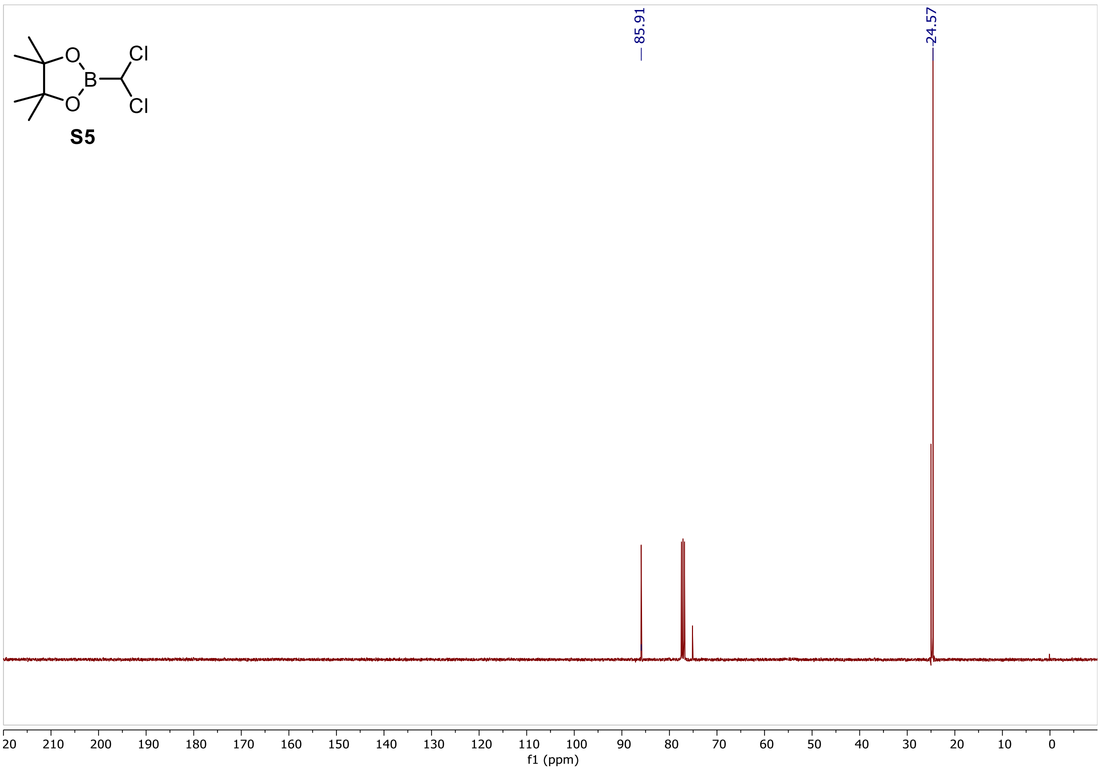

methyl 4-((4S,5R)-2,2-dimethyl-5-((E)-2-(4,4,5,5-tetramethyl-1,3,2-dioxaborolan-2-yl)vinyl)-1,3-dioxolan-4-yl)butanoate (10)

^{1}H-NMR (400 MHz, CDCl$_3$):

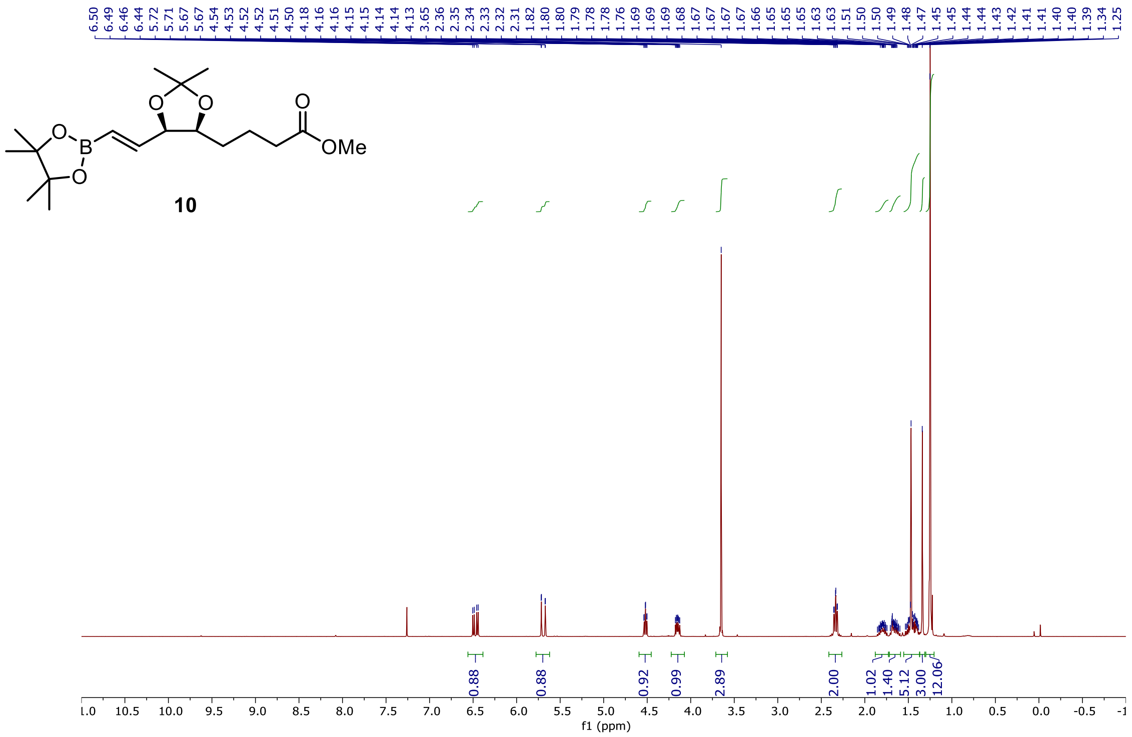

^{13}C-NMR:

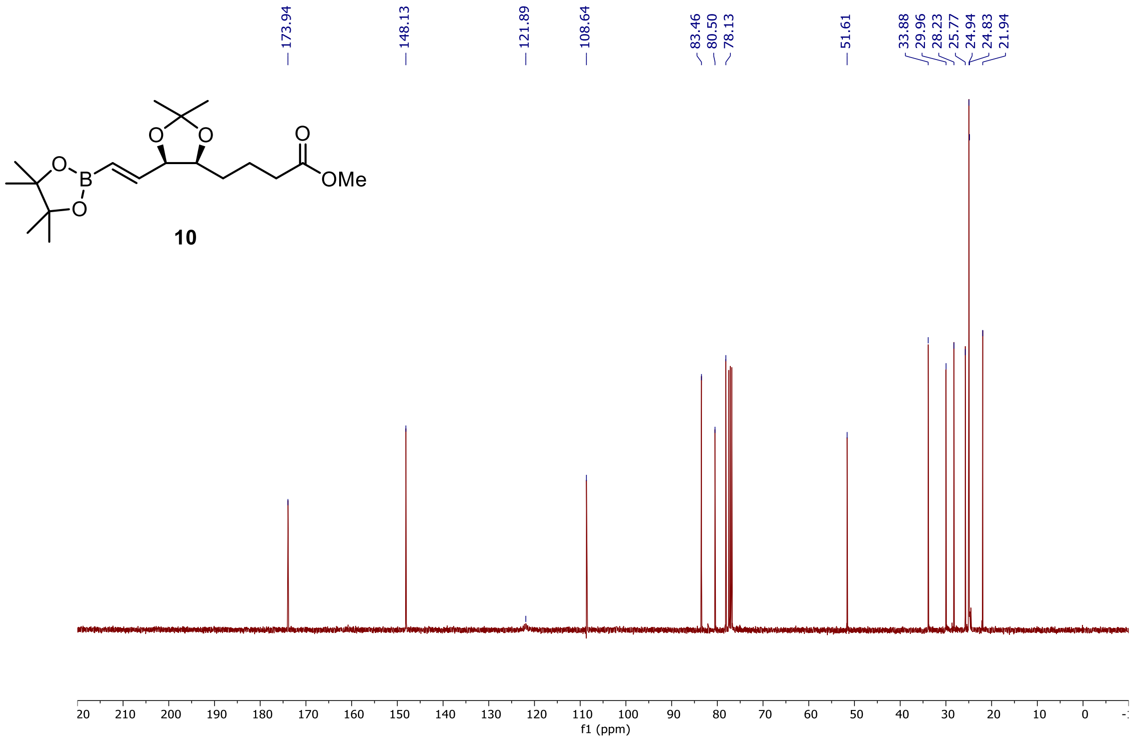

1-(2-bromophenyl)-2-iodoethan-1-one (12)

^{1}H-NMR (400 MHz, $CDCl_3$)**:**

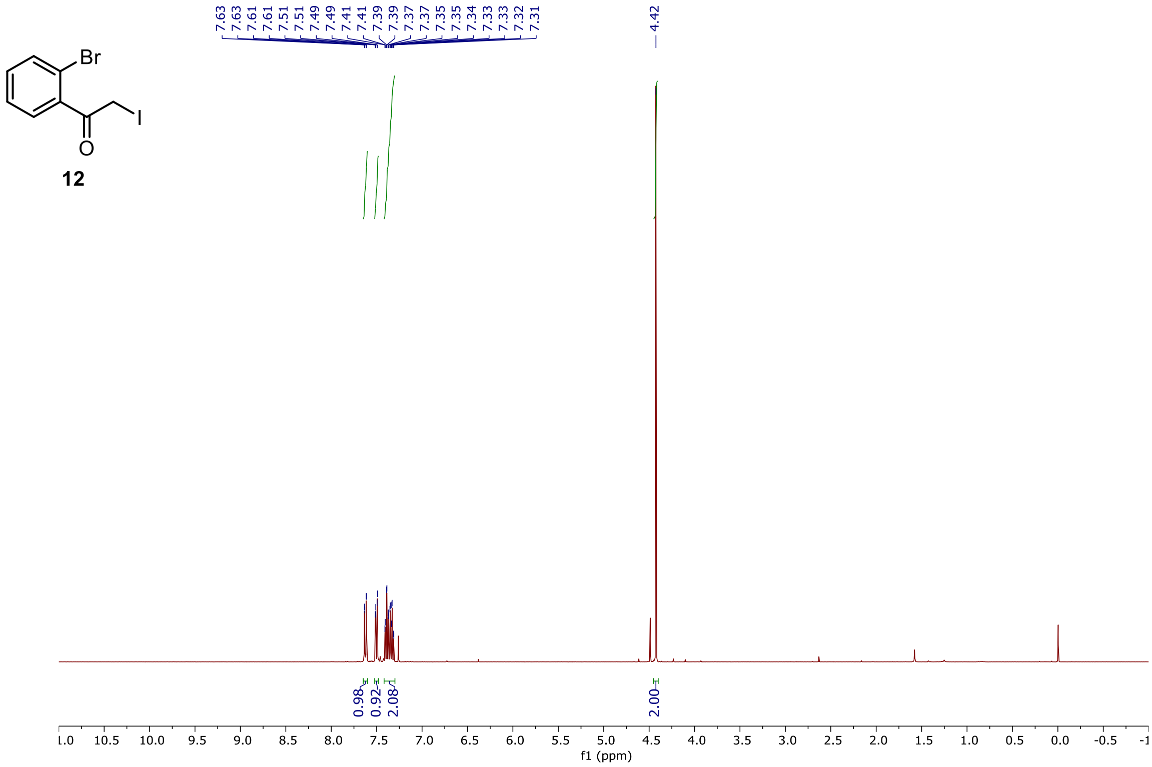

^{13}C-NMR (101 MHz, $CDCl_3$):

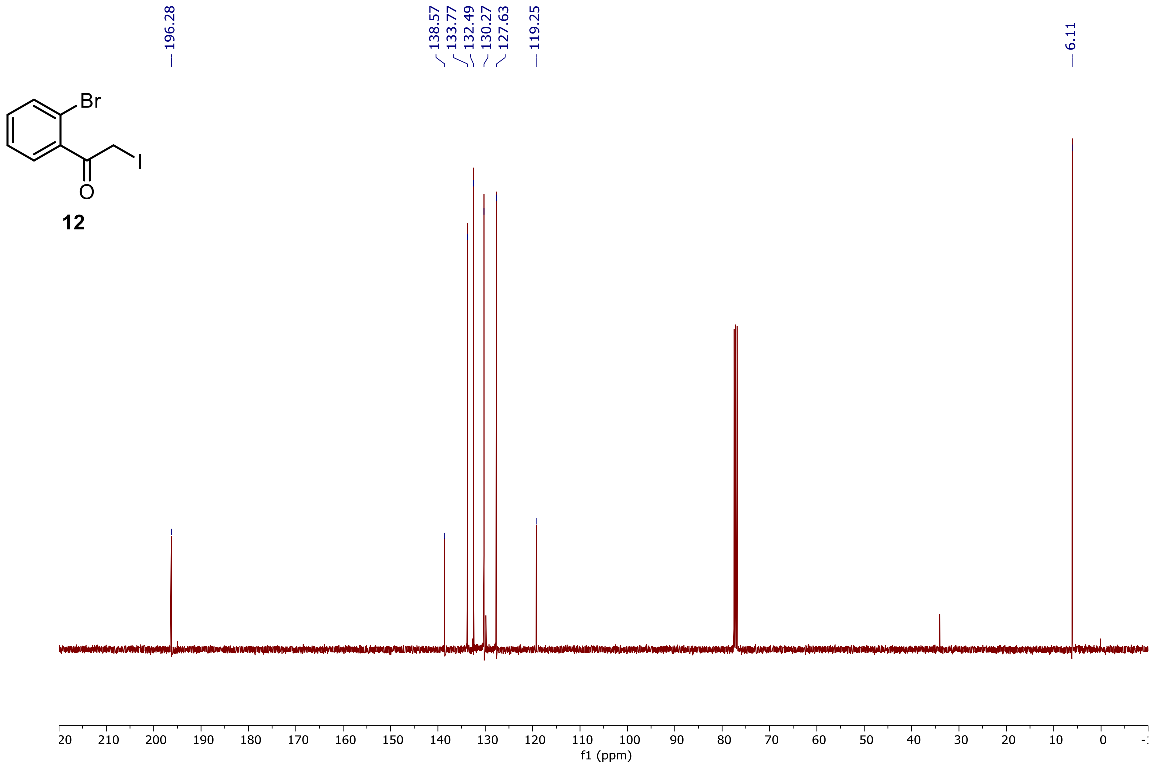

2-(bicyclo[1.1.1]pentan-1-yl)-1-(2-bromophenyl)ethan-1-one (9) and **2-(bicyclo[1.1.1]pentan-1-yl)-1-phenylethan-1-one (18)**

^{1}H-NMR (400 MHz, $CDCl_3$)**:**

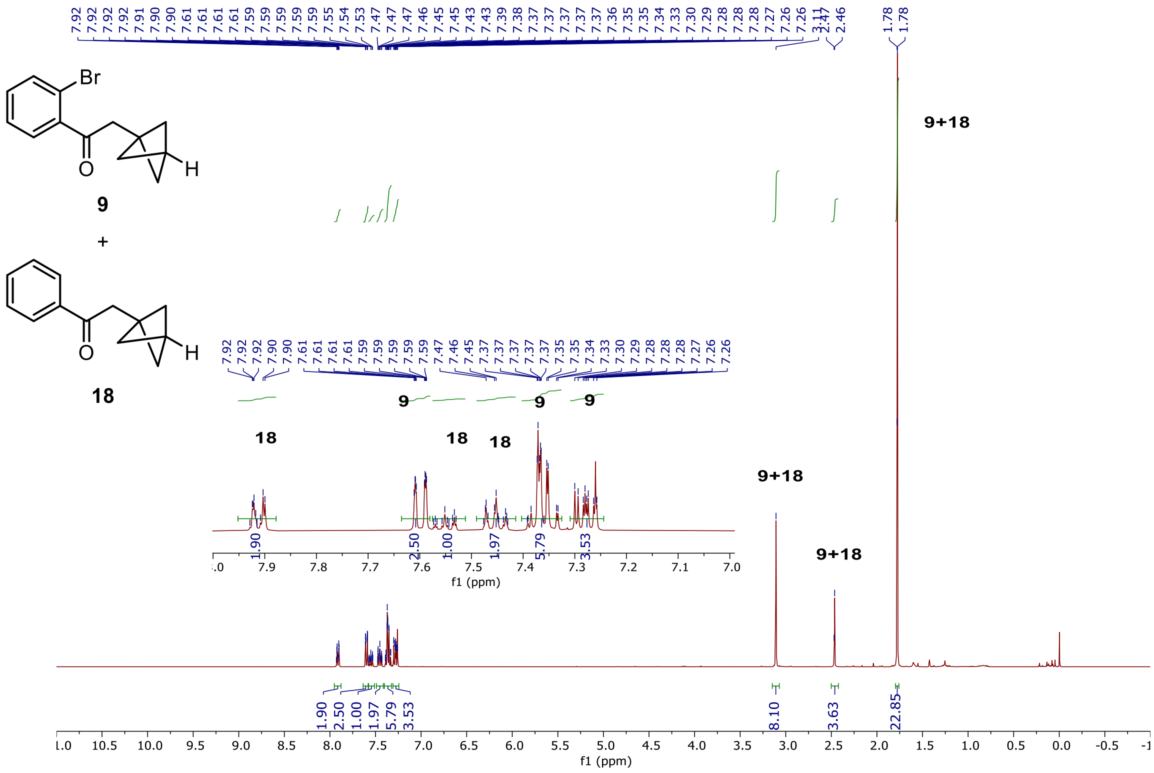

^{13}C-NMR (101 MHz, $CDCl_3$)**:**

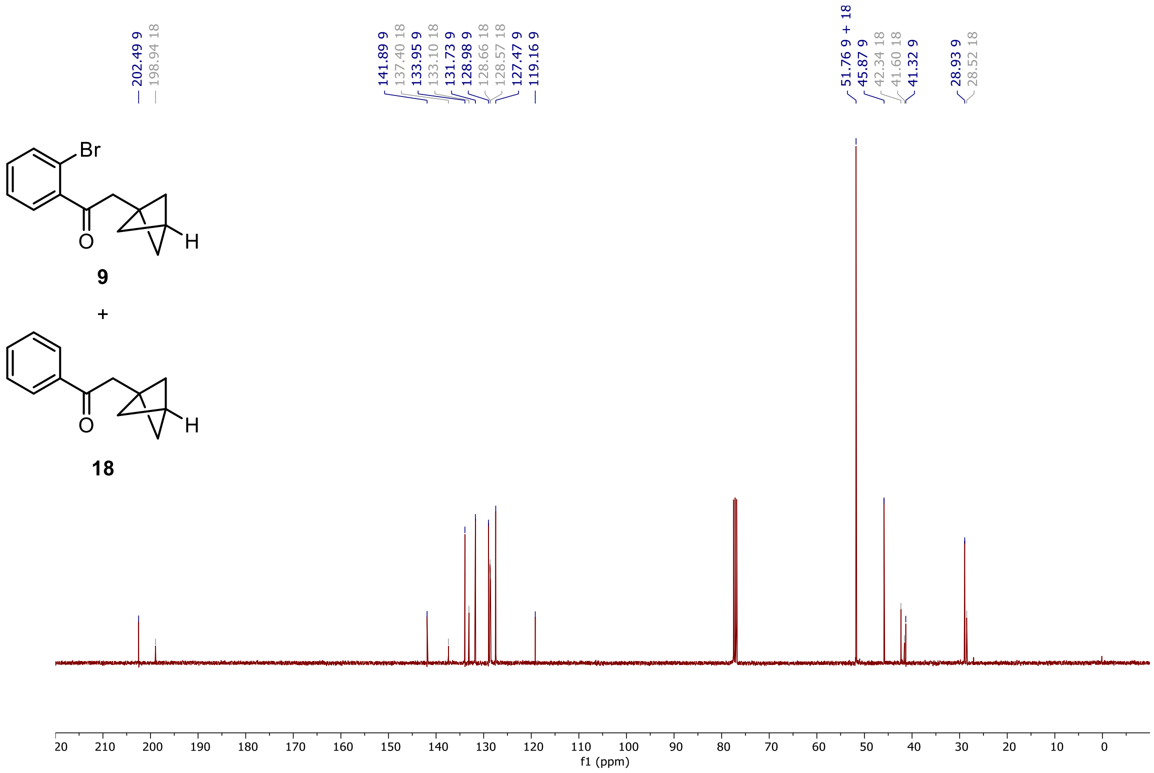

methyl 4-((4*S*,5*R*)-5-((E)-2-(2-(bicyclo[1.1.1]pentan-1-yl)acetyl)styryl)-2,2-dimethyl-1,3-dioxolan-4-yl)butanoate (7)

^{1}H-NMR (400 MHz, $CDCl_3$):

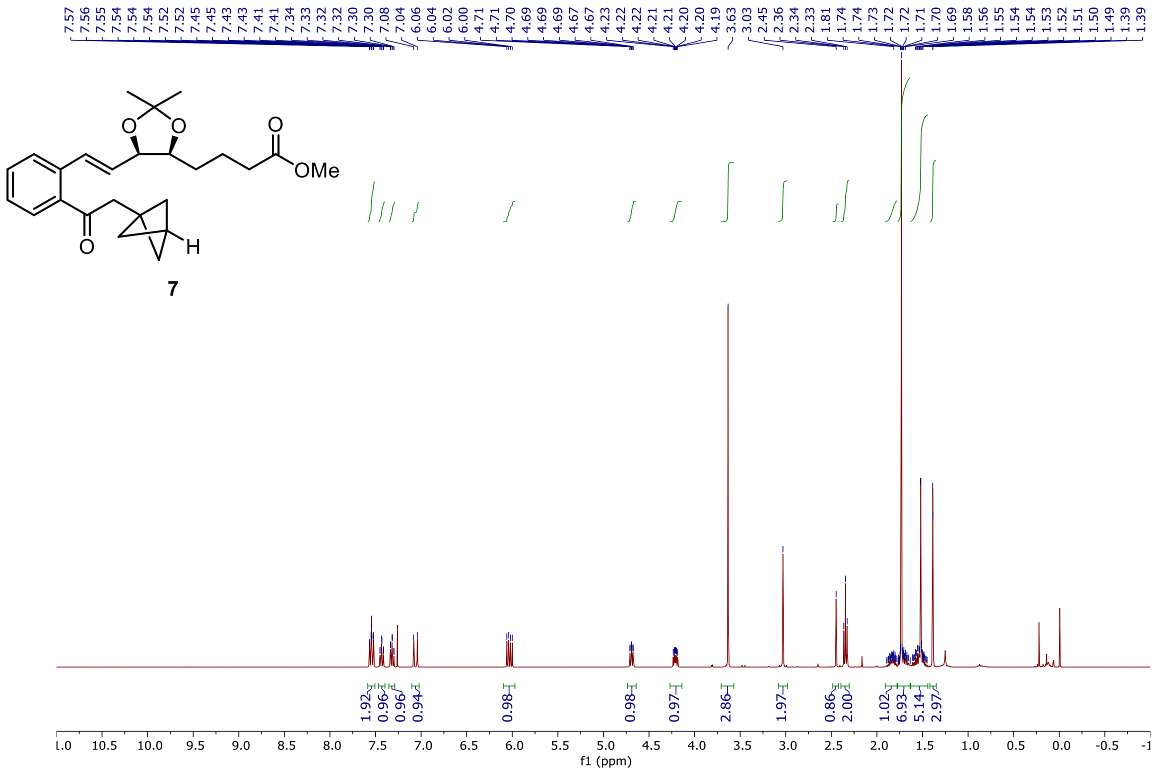

^{13}C-NMR (101 MHz, $CDCl_3$):

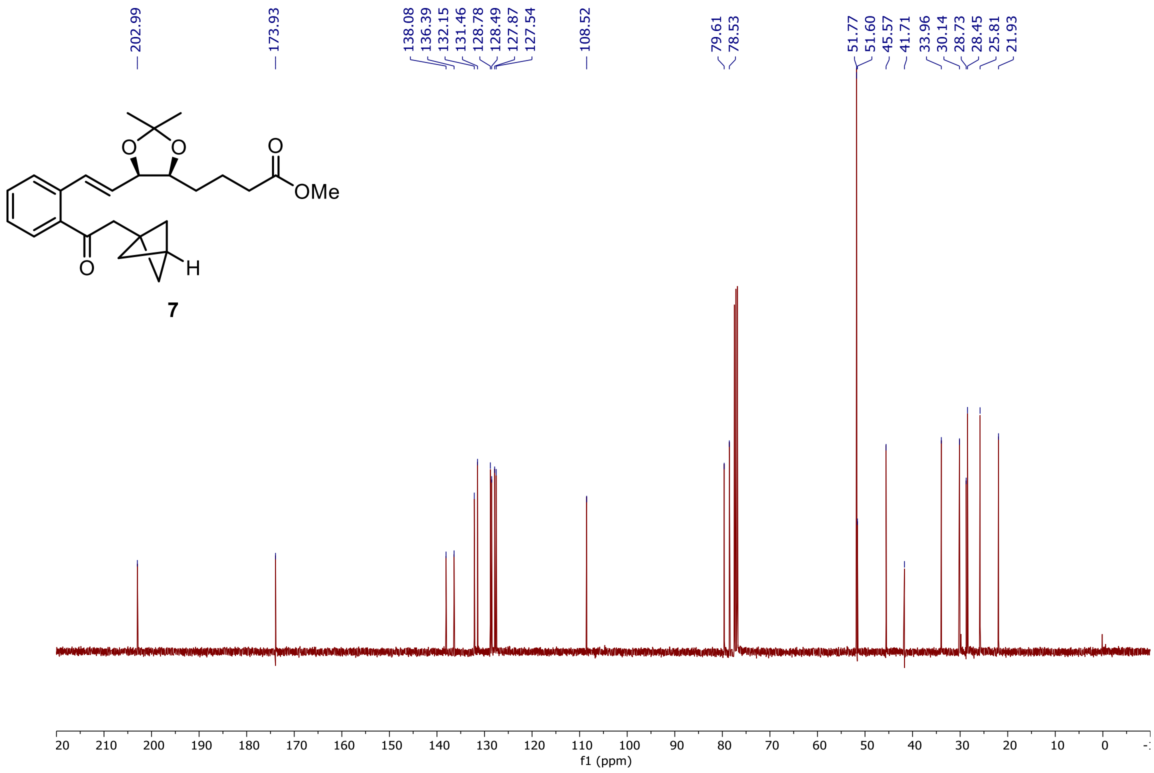

methyl 4-((4*S*,5*R*)-5-((E)-2-((*S*)-2-(bicyclo[1.1.1]pentan-1-yl)-1-hydroxyethyl)styryl)-2,2-dimethyl-1,3-dioxolan-4-yl)butanoate (19a)

^{1}H-NMR (400 MHz, $CDCl_3$)**:**

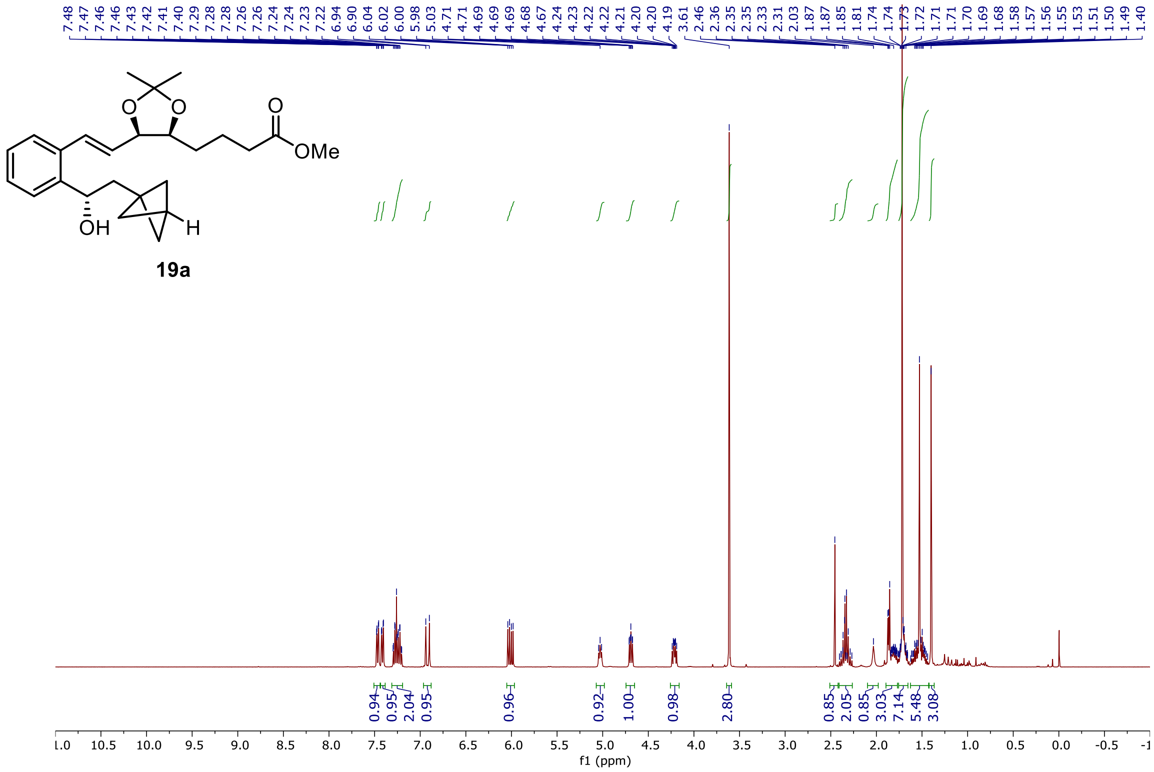

^{13}C-NMR (101 MHz, $CDCl_3$)**:**

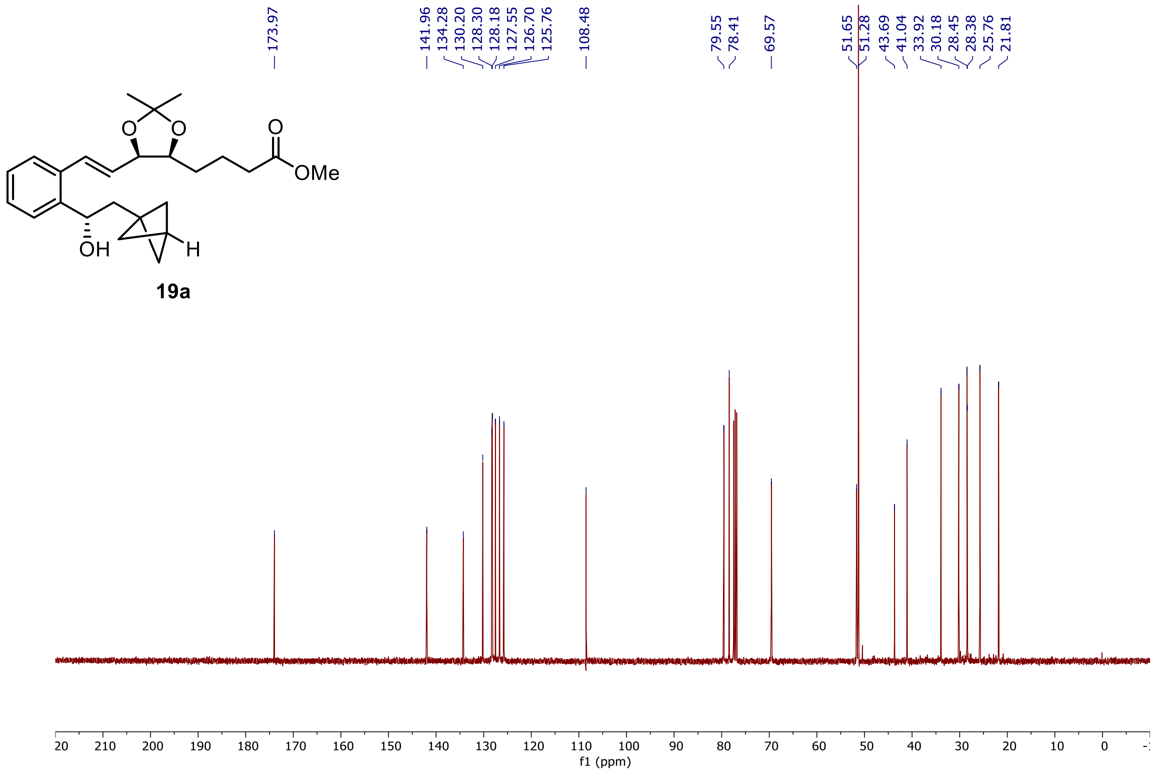

methyl 4-((4*S*,5*R*)-5-(*R*)-2-(*R*)-2-(bicyclo[1.1.1]pentan-1-yl)-1-hydroxyethyl)styryl)-2,2-dimethyl-1,3-dioxolan-4-yl)butanoate (19b)

^{1}H-NMR (400 MHz, $CDCl_3$)**:**

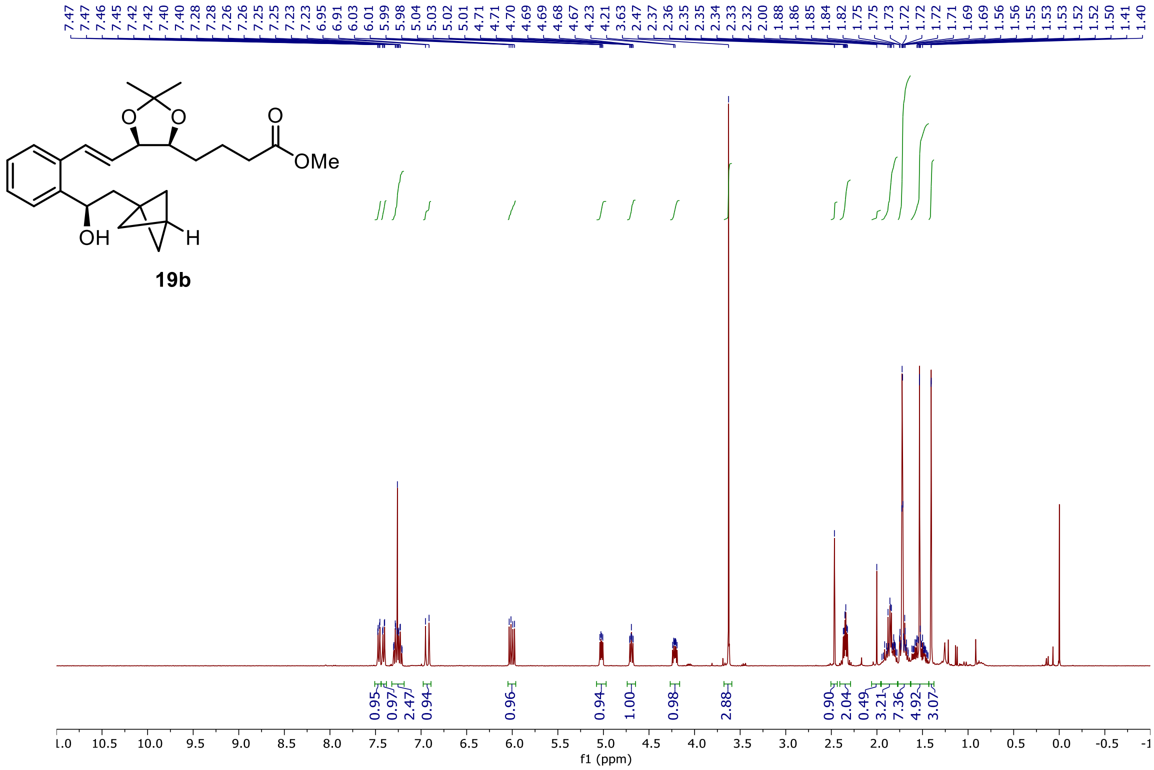

^{13}C-NMR (101 MHz, $CDCl_3$)**:**

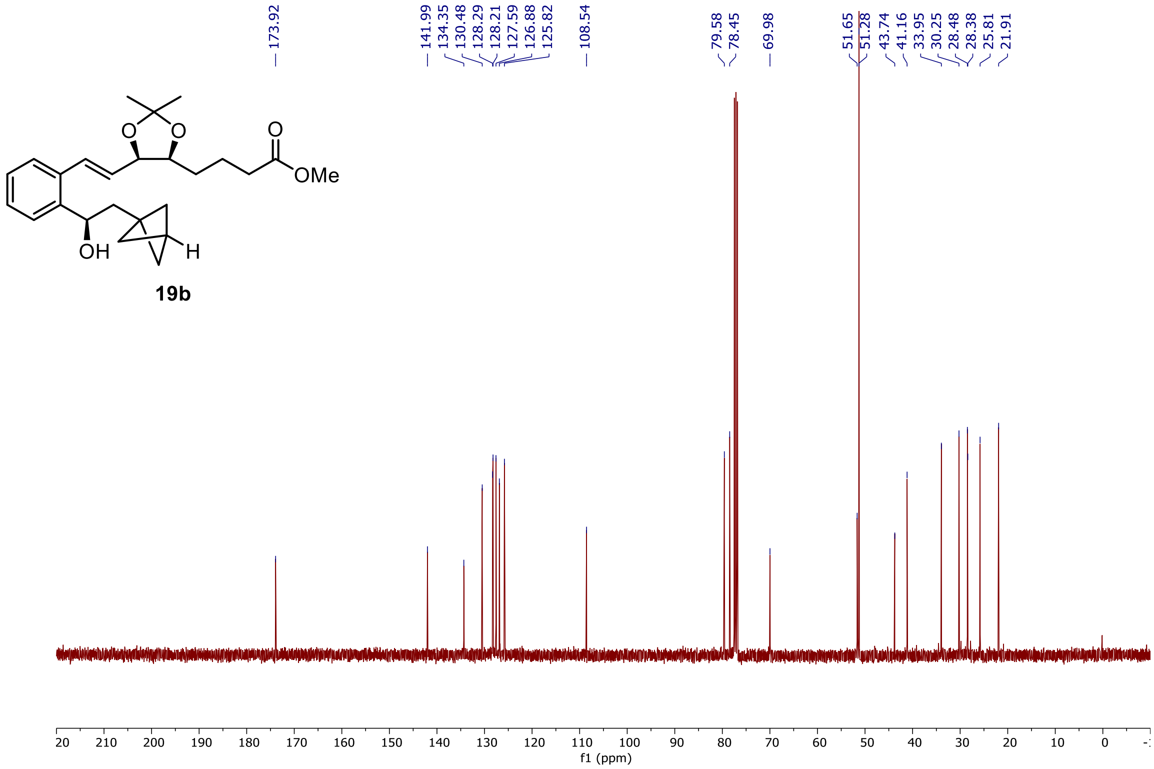

methyl (5*S*,6*R*,*E*)-8-(2-((*S*)-2-(bicyclo[1.1.1]pentan-1-yl)-1-hydroxyethyl)phenyl)-5,6-dihydroxyoct-7-enoate (5a)

^{1}H-NMR (500 MHz, $CDCl_3$):

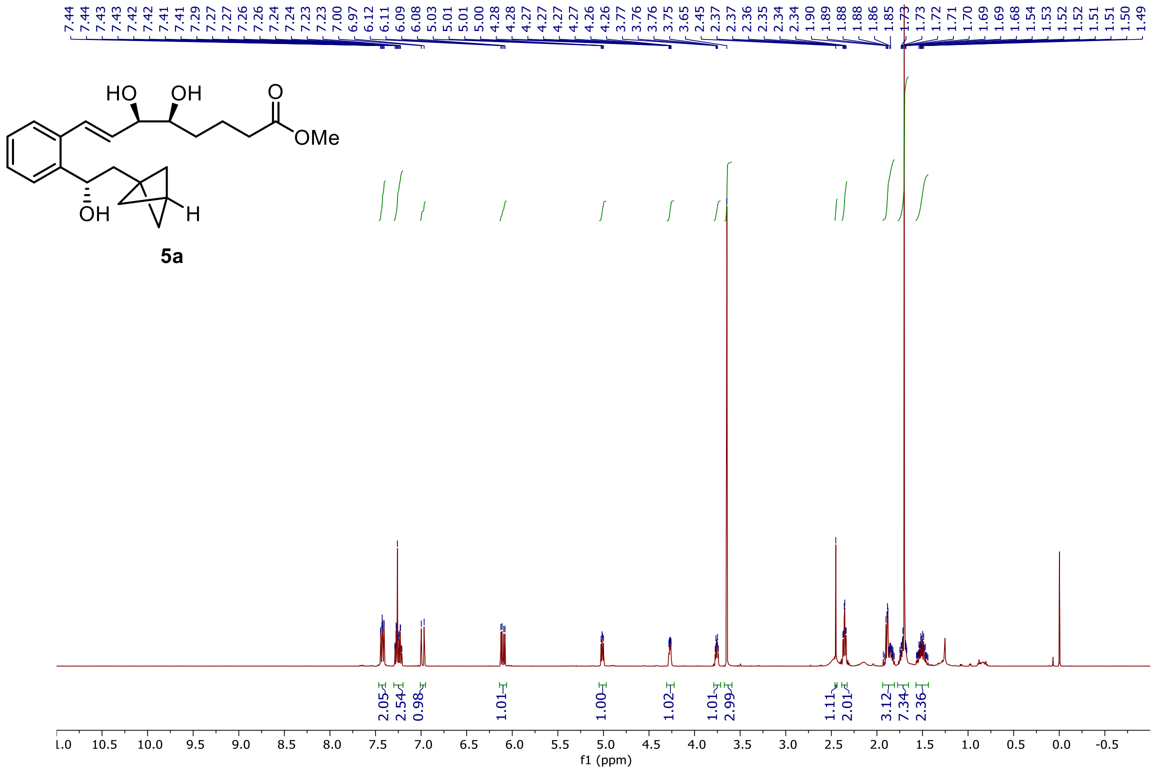

^{13}C-NMR (126 MHz, $CDCl_3$):

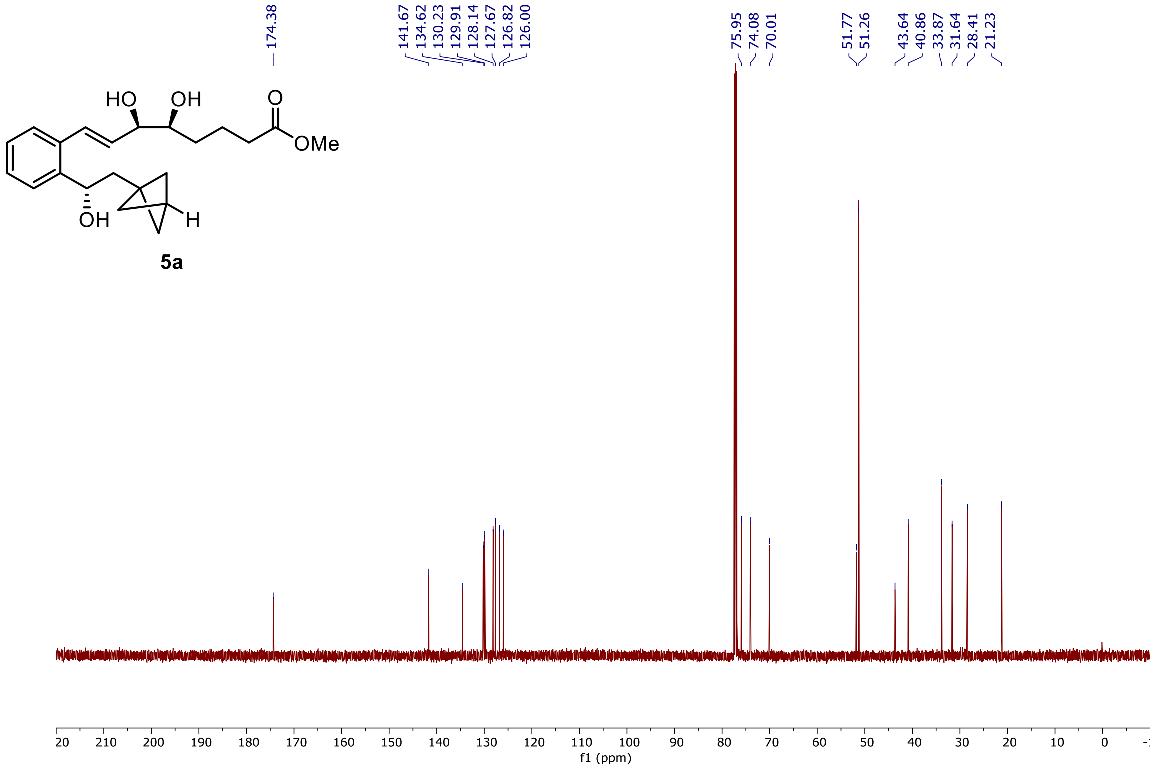

COSY:

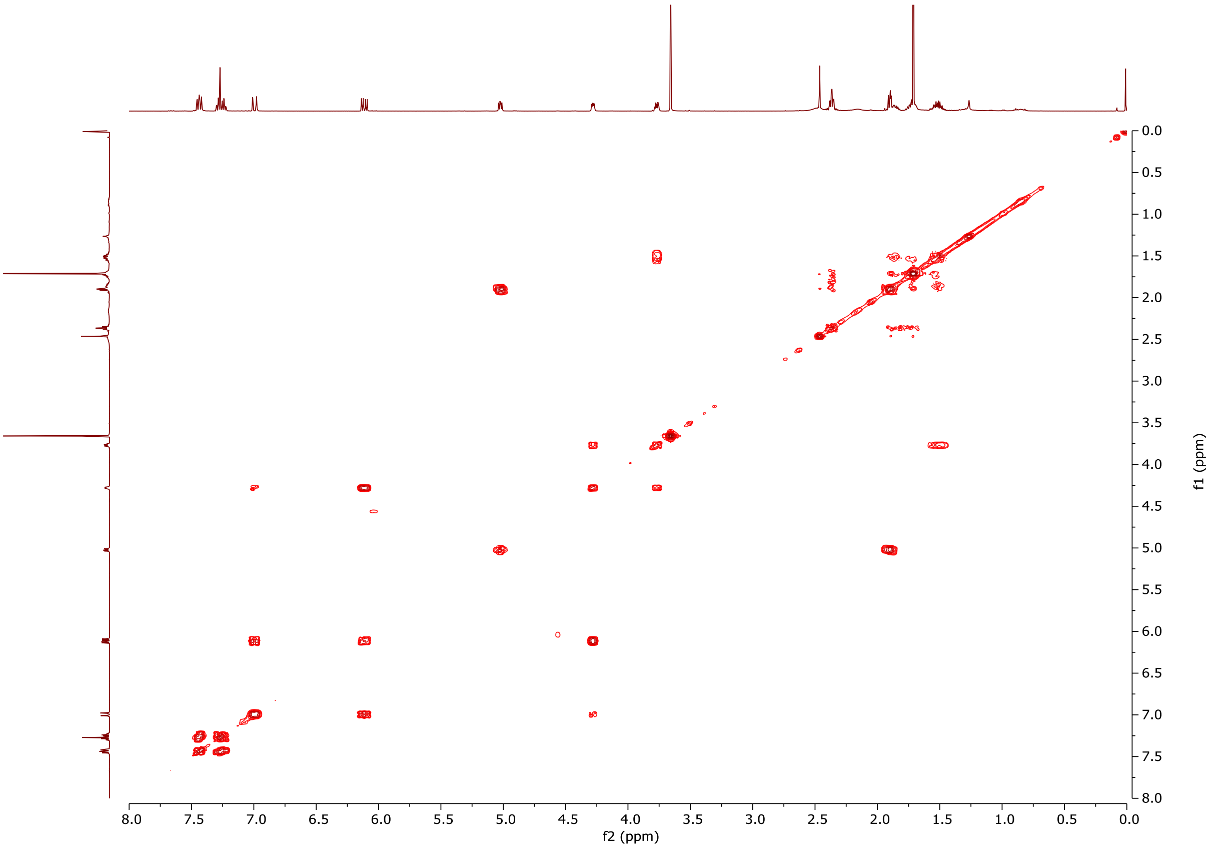

HSQC:

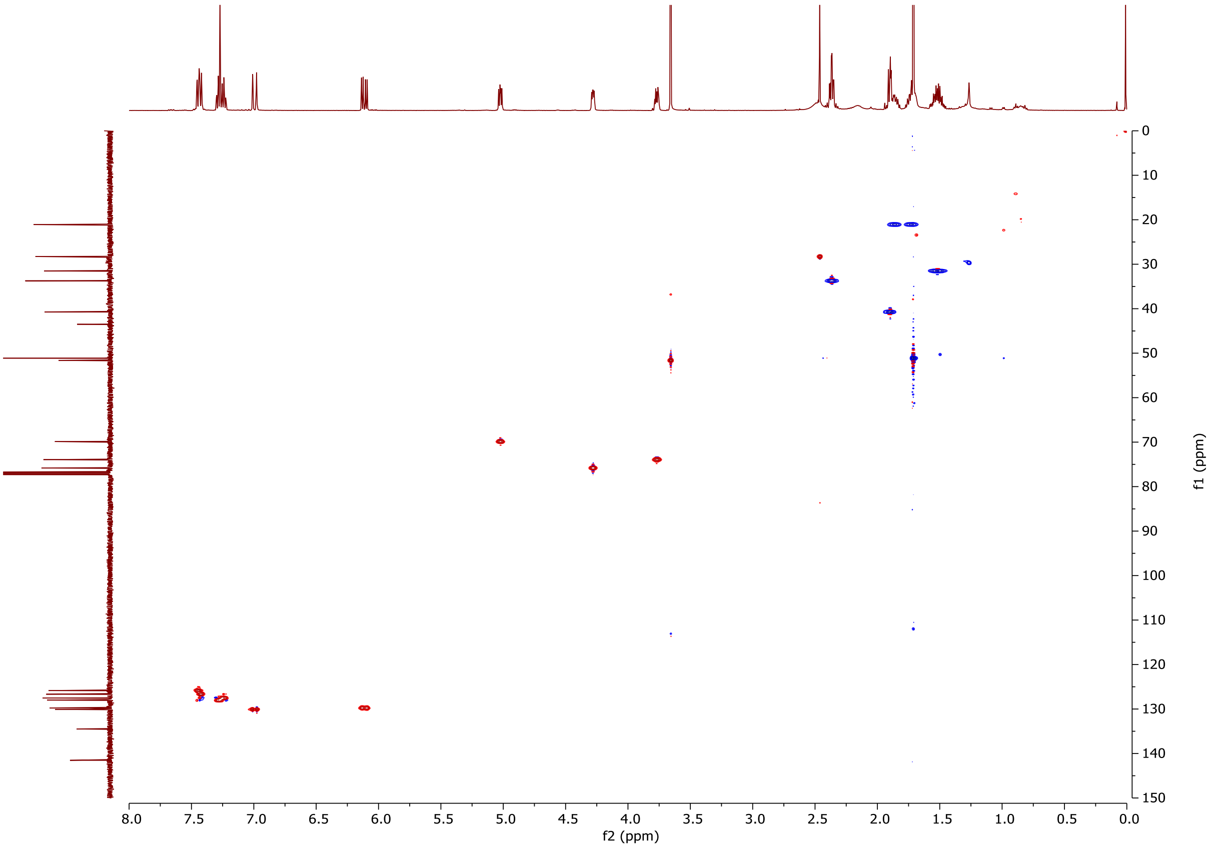

methyl (5*S*,6*R*,*E*)-8-(2-((*R*)-2-(bicyclo[1.1.1]pentan-1-yl)-1-hydroxyethyl)phenyl)-5,6-dihydroxyoct-7-enoate (5b)

[1]**H-NMR** (500 MHz, $CDCl_3$)**:**

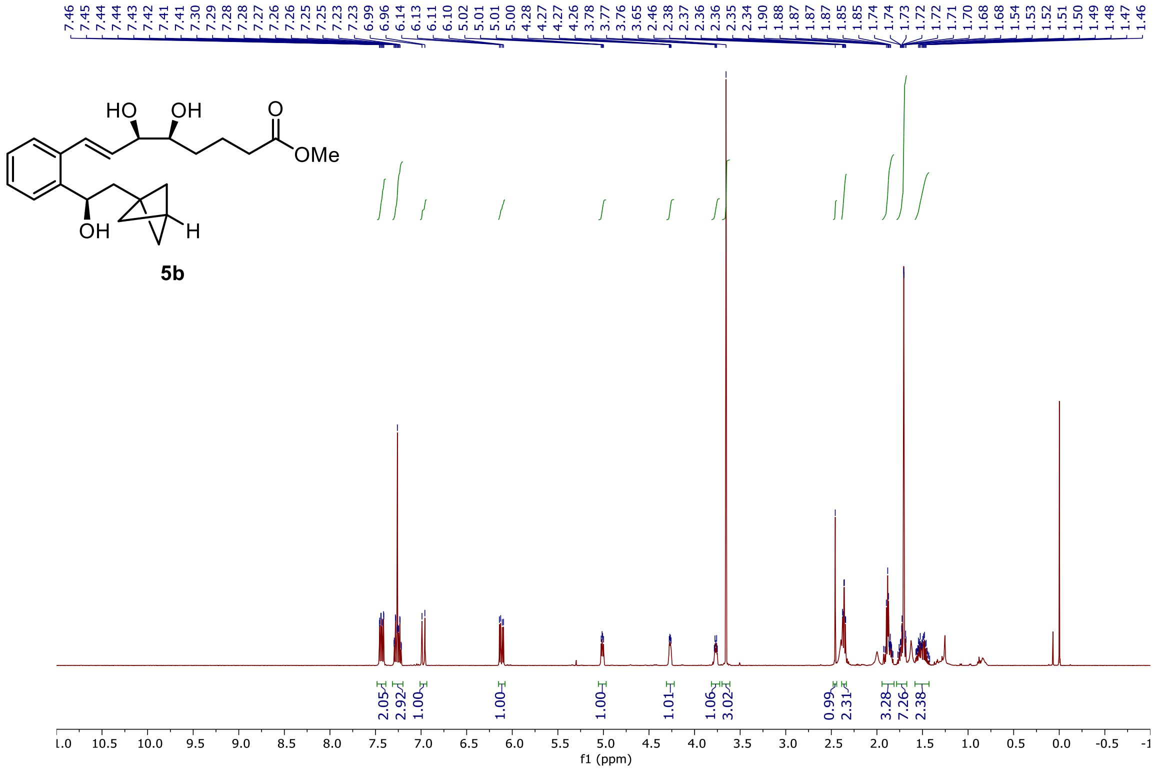

[13]**C-NMR** (126 MHz, $CDCl_3$)**:**

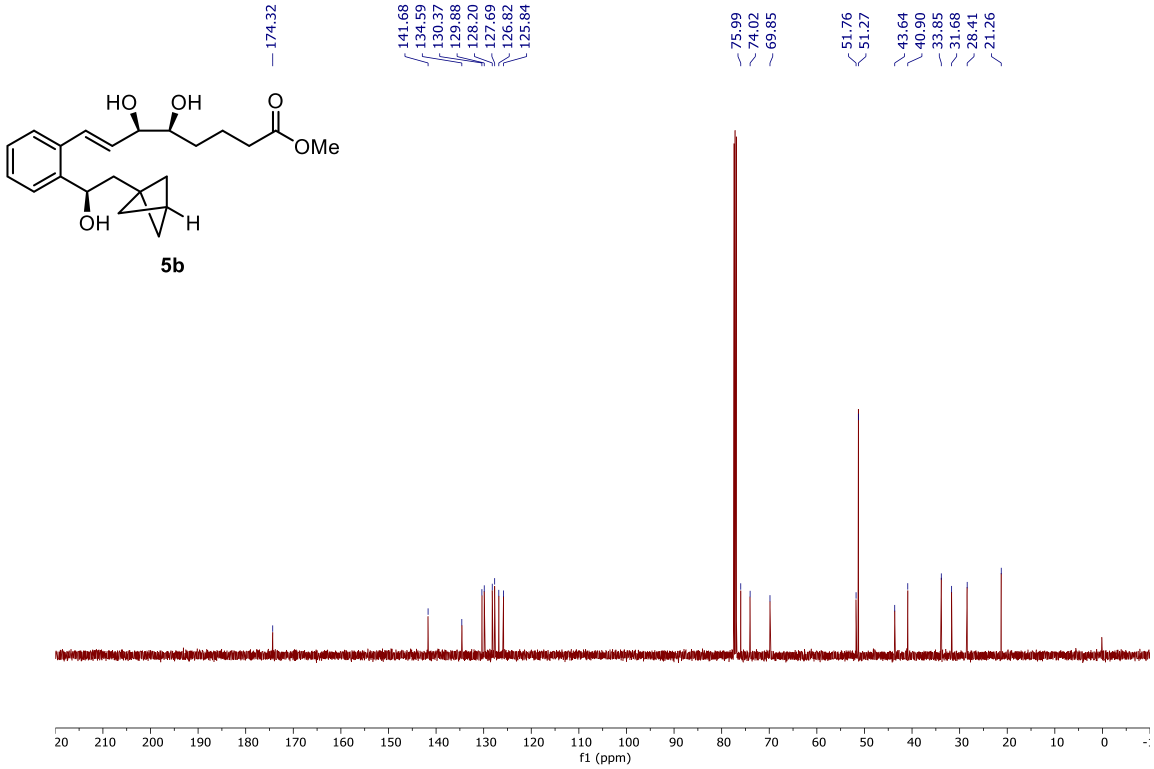

COSY:

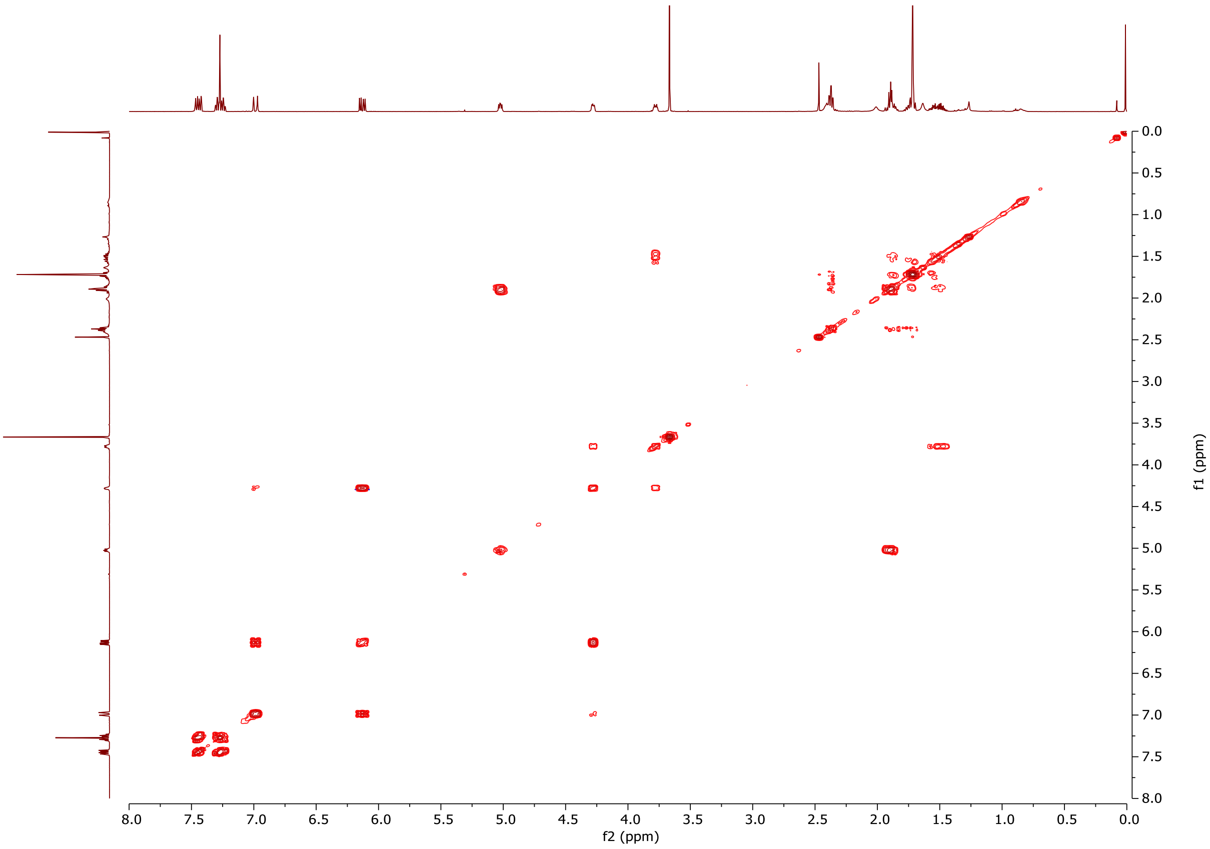

HSQC:

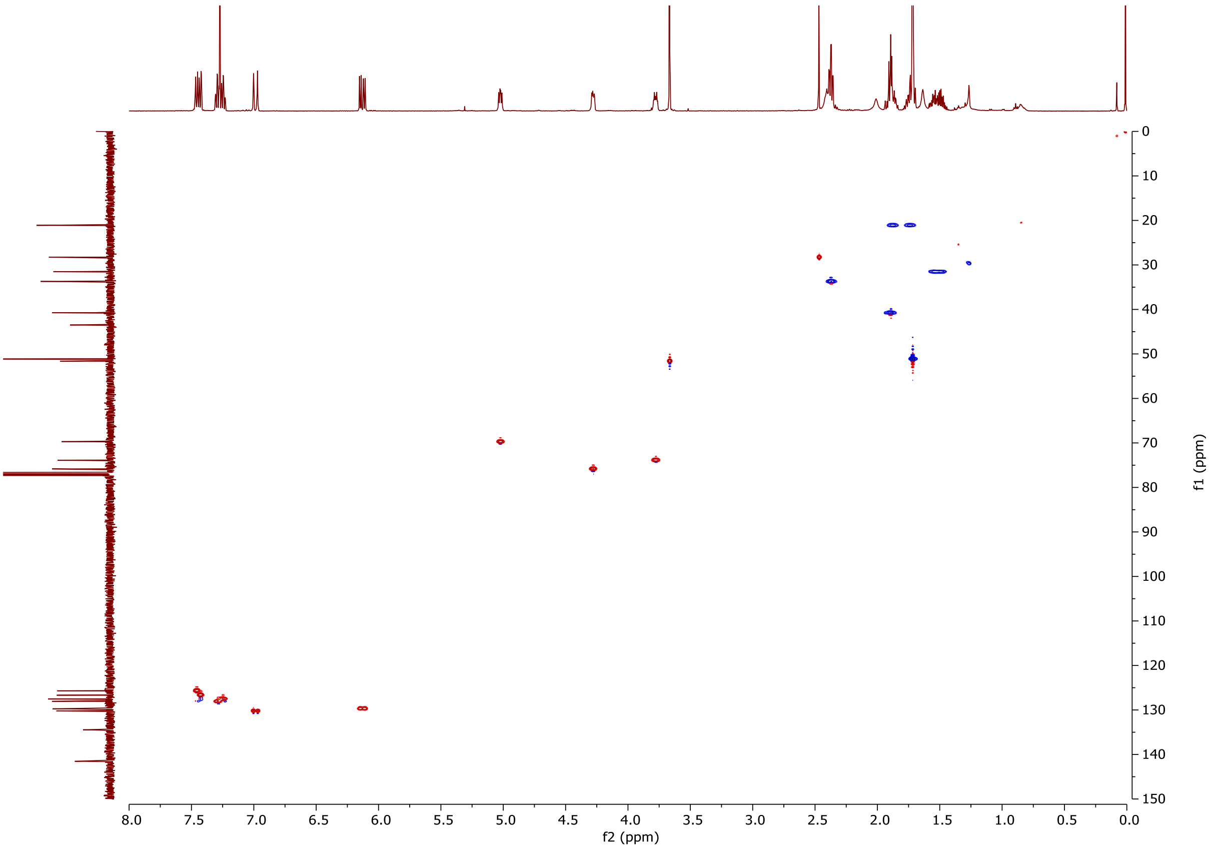

4,5-dibromo-1,2-dimethyl-1H-imidazole (21)

^{1}H-NMR (400 MHz, CDCl$_3$)**:**

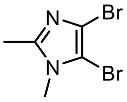

21

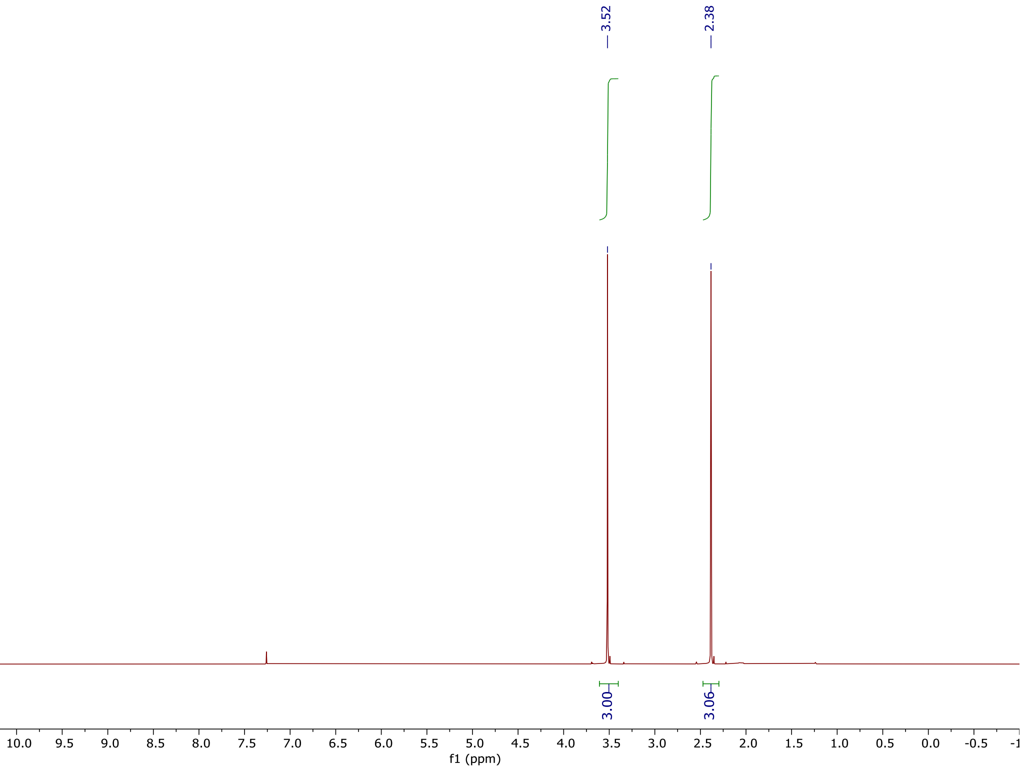

^{13}C-NMR (101 MHz, CDCl$_3$)**:**

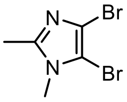

21

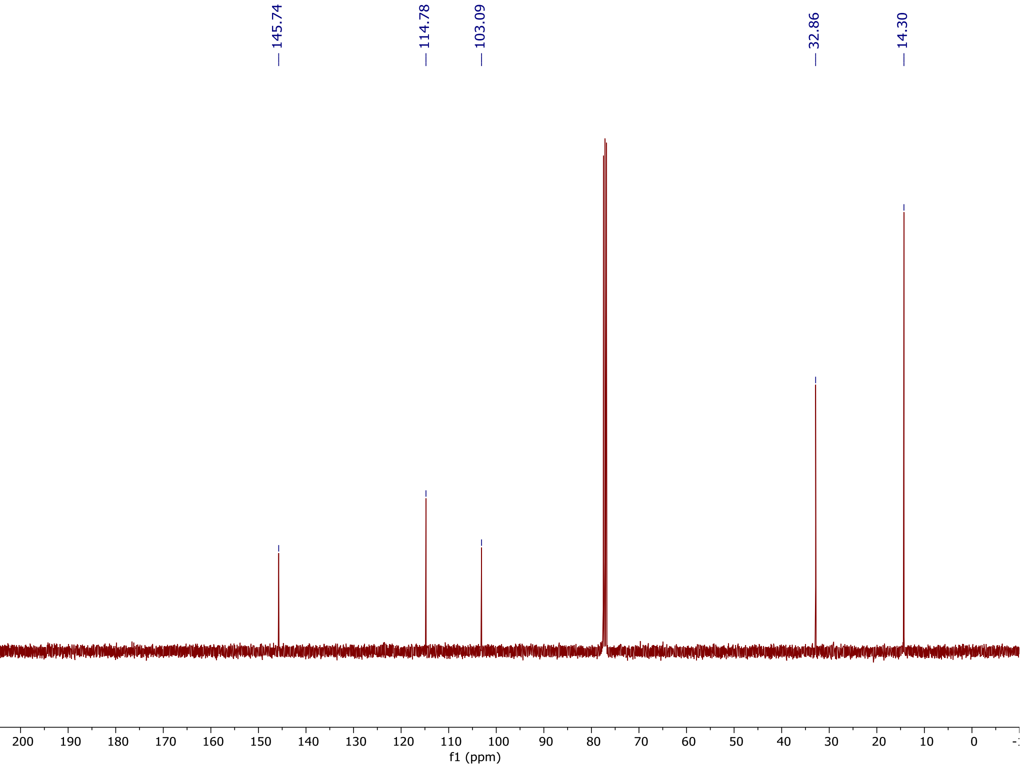

1-(1,2,4-trimethyl-1H-imidazol-5-yl)ethan-1-one (22)

^{1}H-NMR (400 MHz, $CDCl_3$)**:**

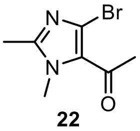

22

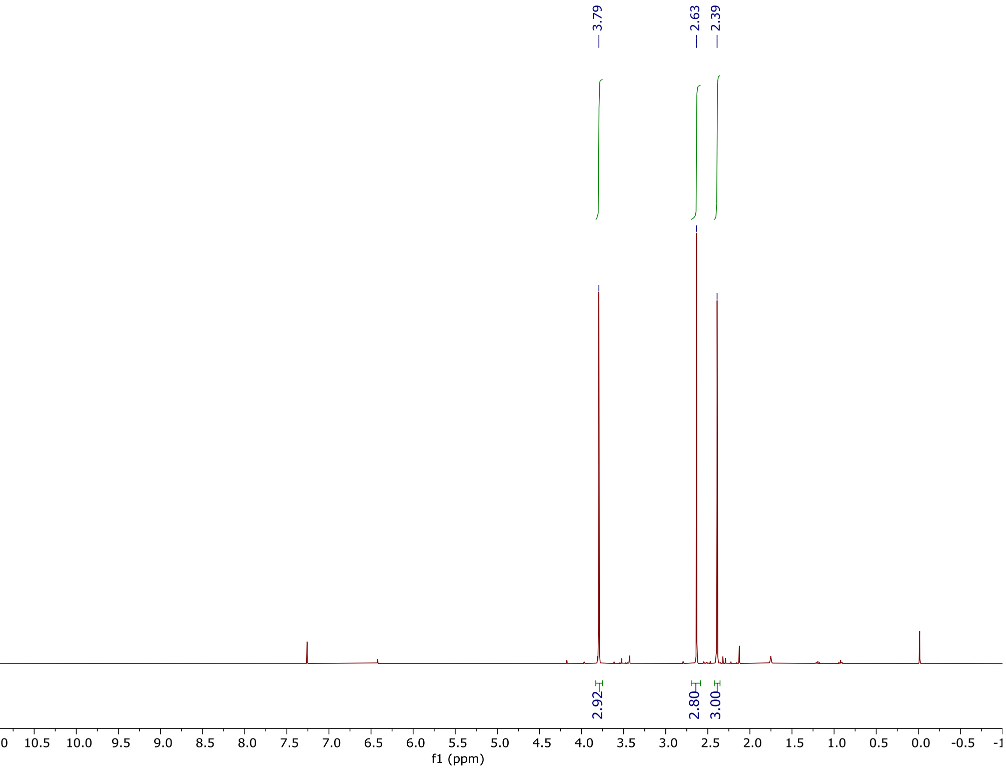

^{13}C-NMR (101 MHz, $CDCl_3$)**:**

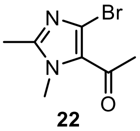

22

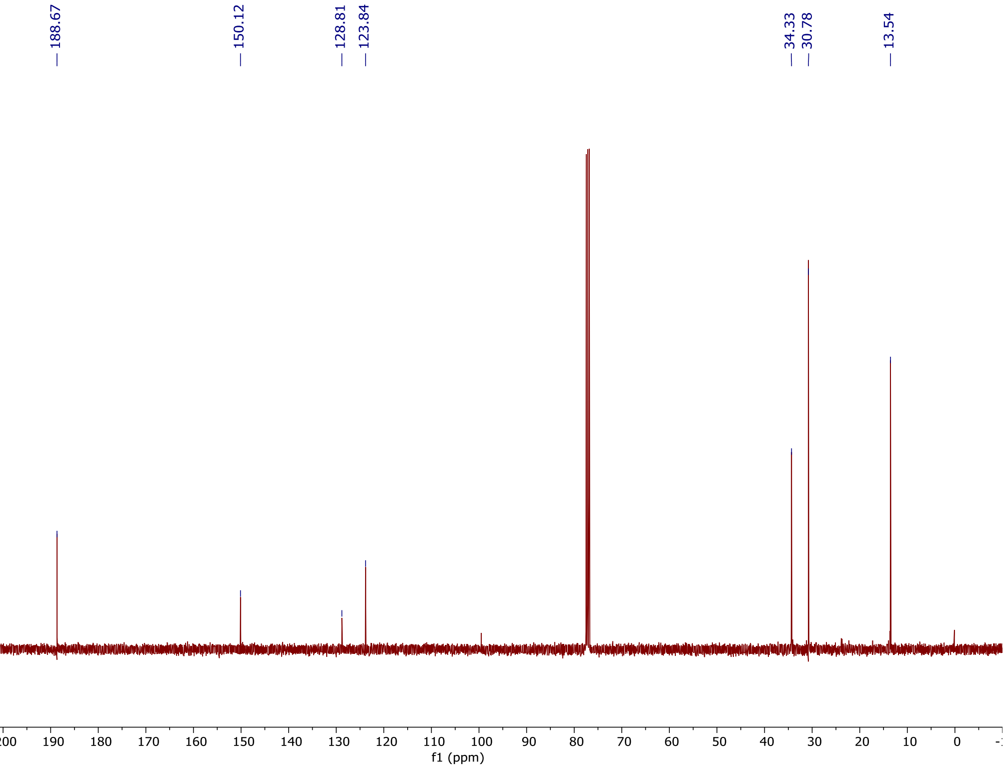

1-(4-bromo-1,2-dimethyl-1H-imidazol-5-yl)-2-iodoethan-1-one (15)

^{1}H-NMR (400 MHz, $CDCl_3$)**:**

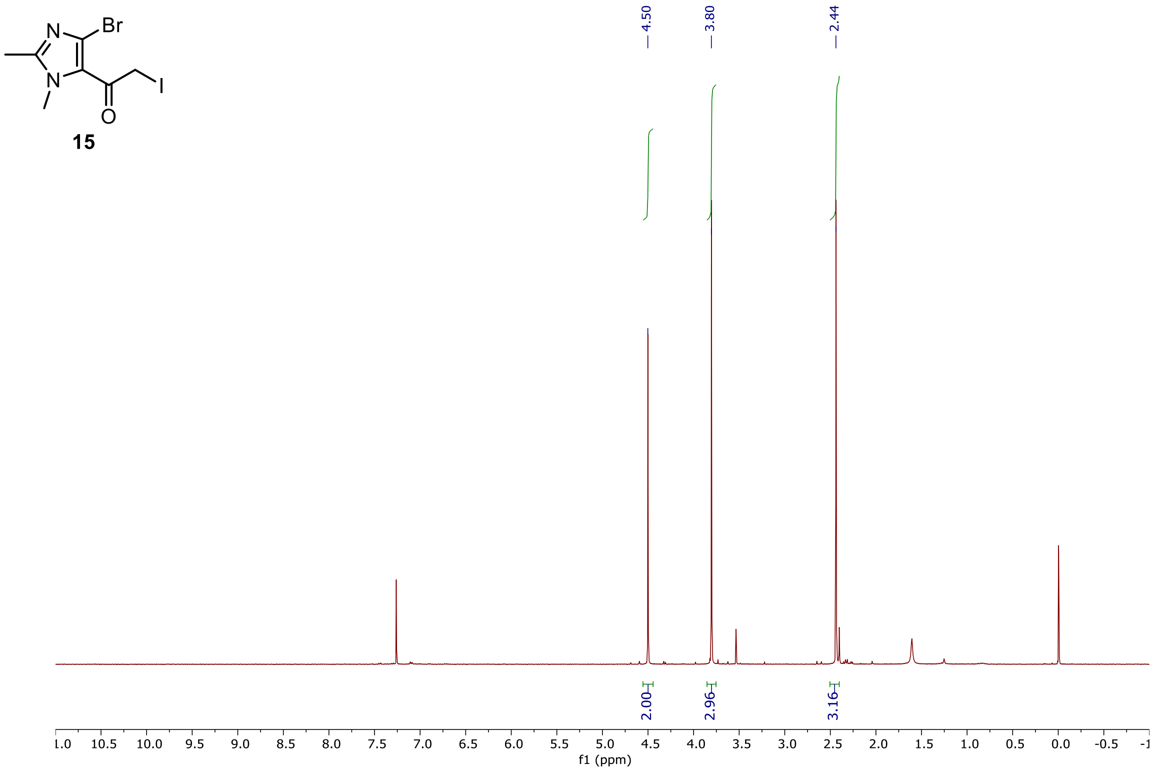

^{13}C-NMR (101 MHz, $CDCl_3$)**:**

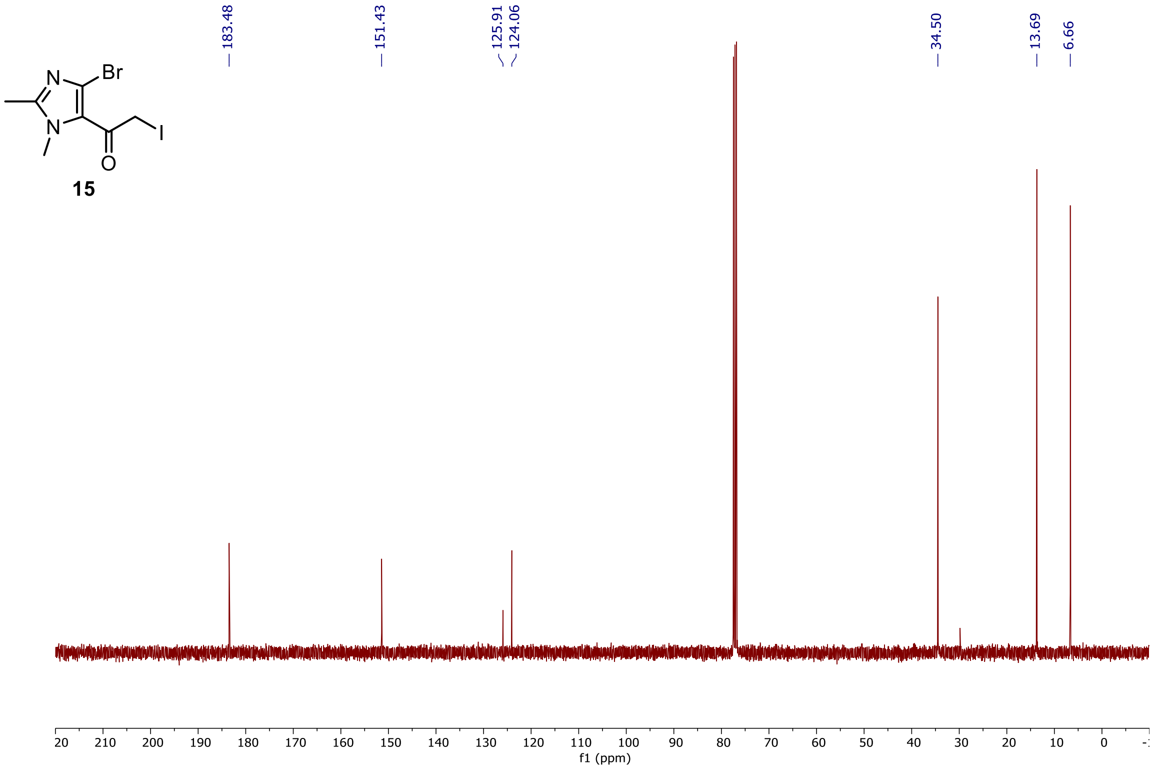

2-(bicyclo[1.1.1]pentan-1-yl)-1-(4-bromo-1,2-dimethyl-1H-imidazol-5-yl)ethan-1-one (24)

^{1}H-NMR (400 MHz, $CDCl_3$):

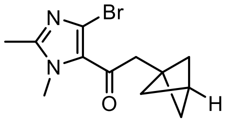

24

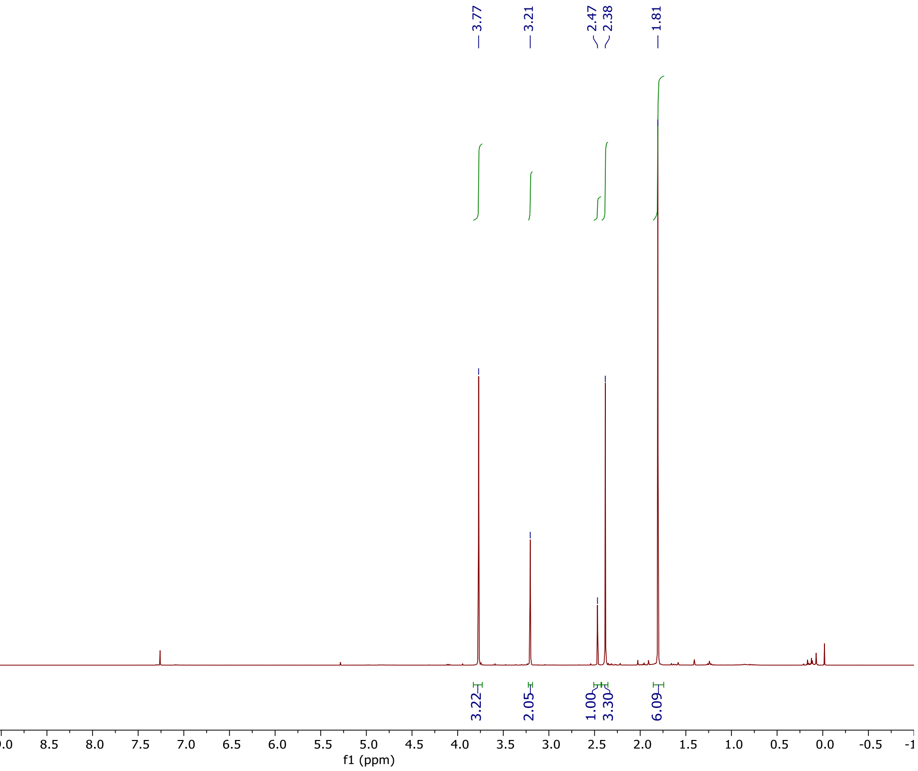

^{13}C-NMR (101 MHz, $CDCl_3$):

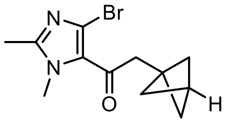

24

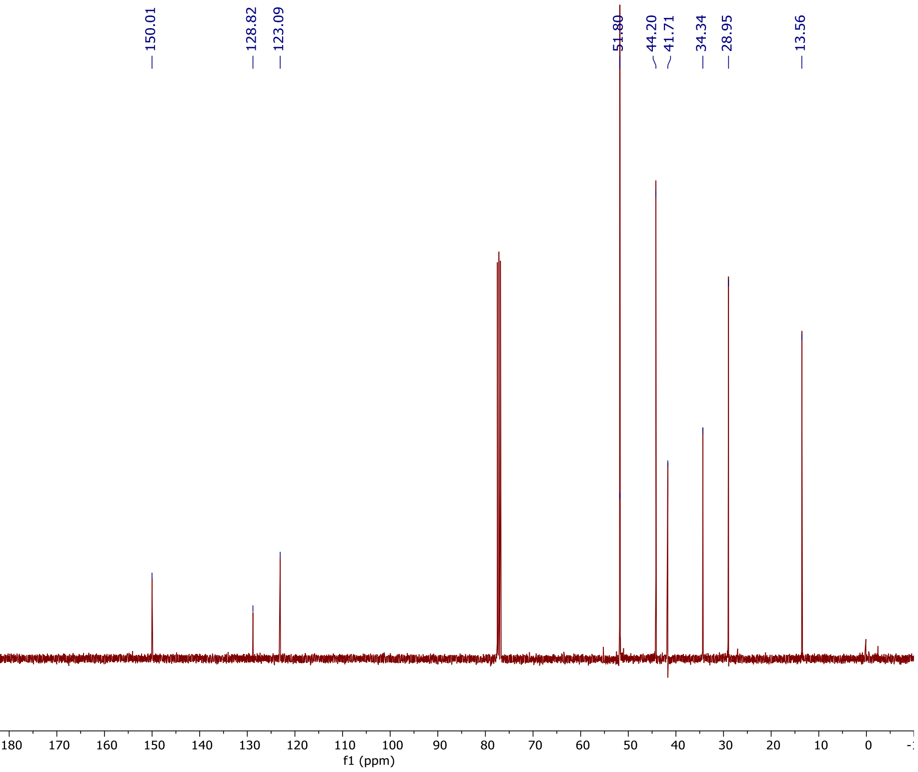

(*S*)-2-(bicyclo[1.1.1]pentan-1-yl)-1-(4-bromo-1,2-dimethyl-1H-imidazol-5-yl)ethan-1-ol ((*S*)-11)

^{1}H-NMR (500 MHz, $CDCl_3$):

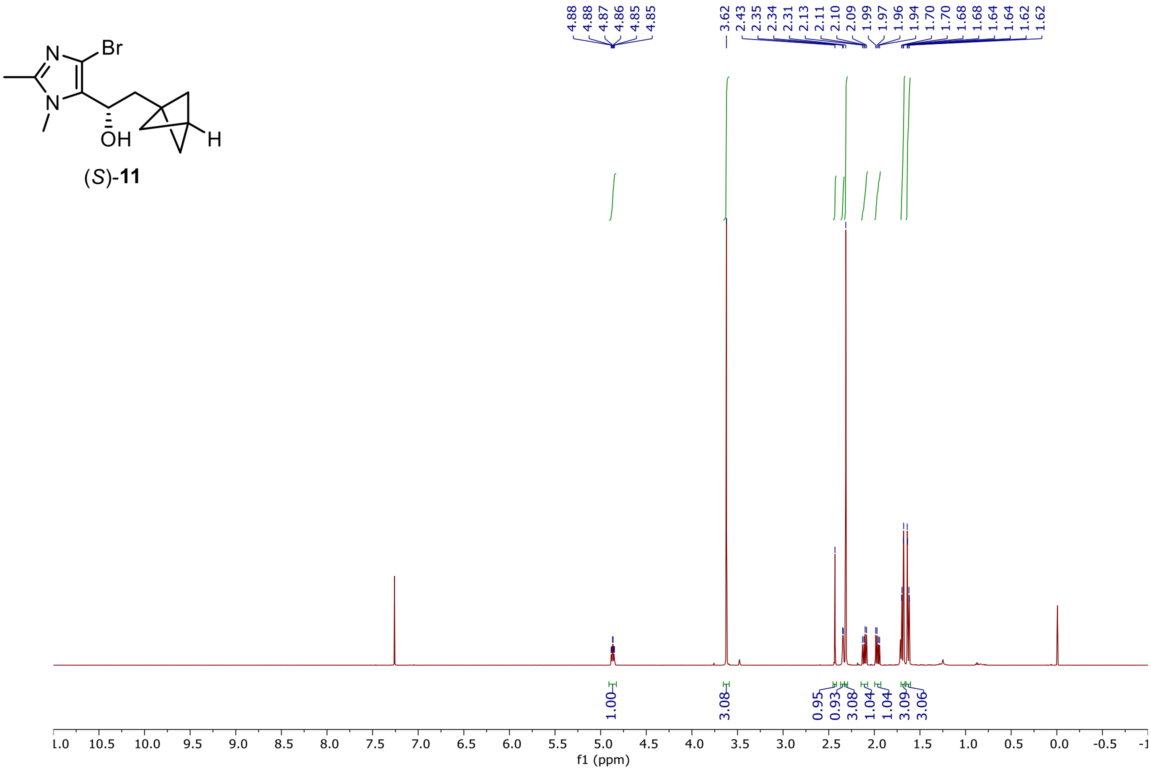

^{13}C-NMR (101 MHz, $CDCl_3$):

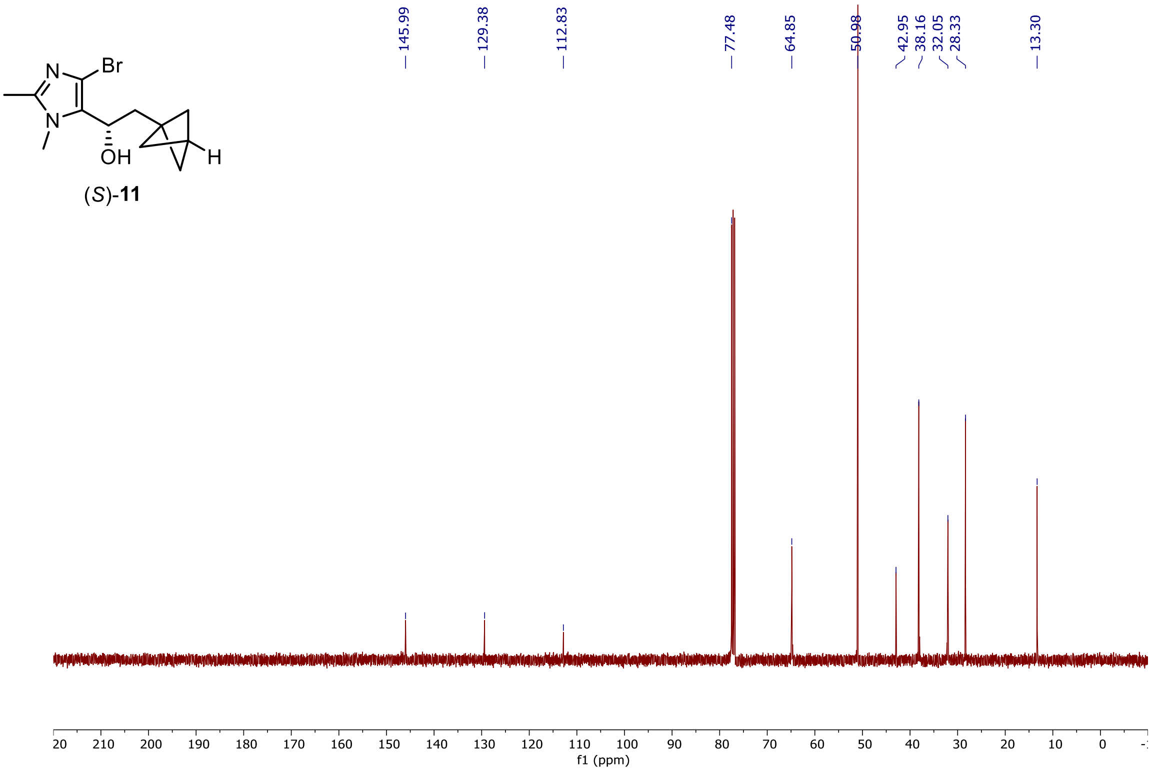

(*R*)-2-(bicyclo[1.1.1]pentan-1-yl)-1-(4-bromo-1,2-dimethyl-1H-imidazol-5-yl)ethan-1-ol ((*R*)-11)

^{1}H-NMR (500 MHz, $CDCl_3$)**:**

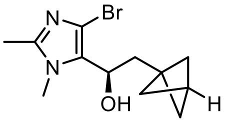

(*R*)-**11**

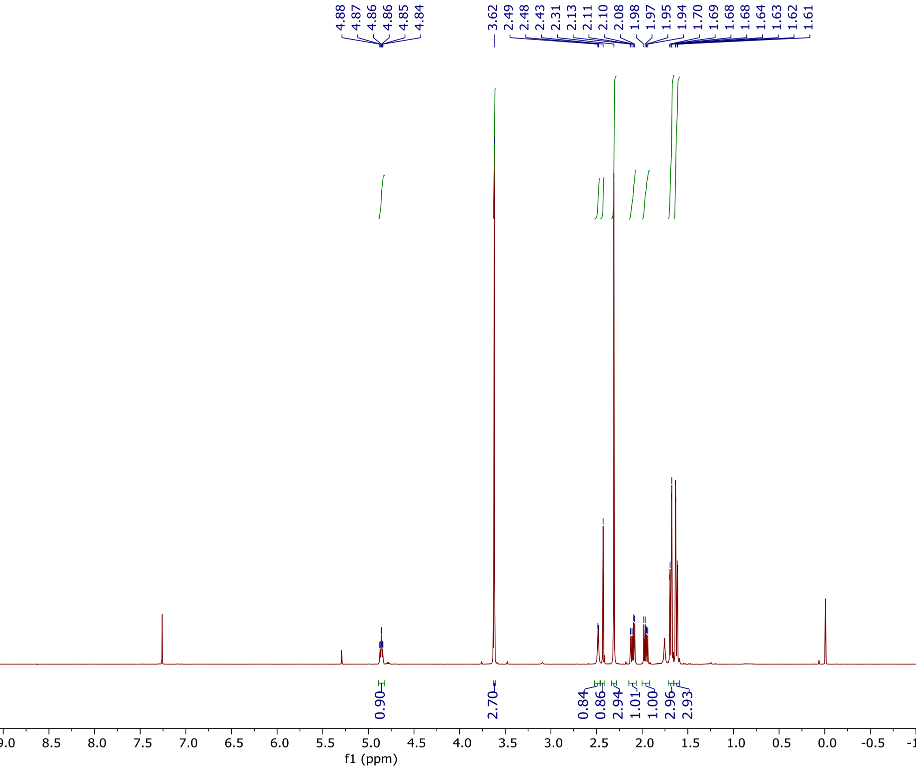

^{13}C-NMR (126 MHz, $CDCl_3$)**:**

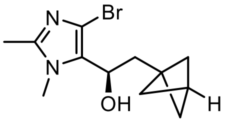

(*R*)-**11**

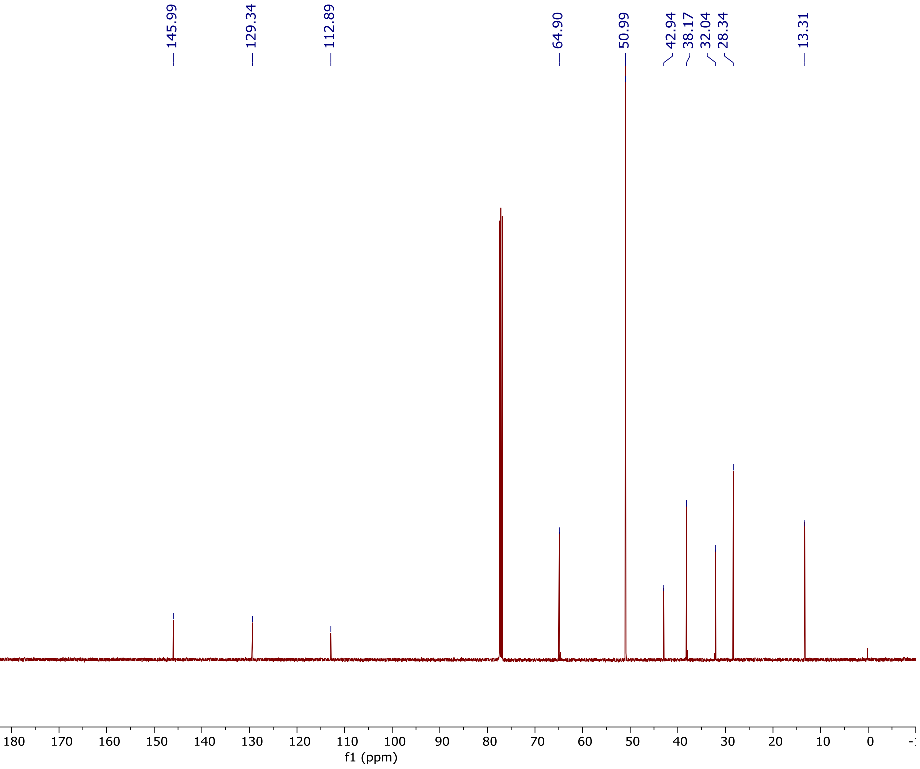

methyl 4-((4*S*,5*R*)-5-((E)-2-(5-((*S*)-2-(bicyclo[1.1.1]pentan-1-yl)-1-hydroxyethyl)-1,2-dimethyl-1H-imidazol-4-yl)vinyl)-2,2-dimethyl-1,3-dioxolan-4-yl)butanoate (8a)

^{1}H-HMR (400 MHz, $CDCl_3$):

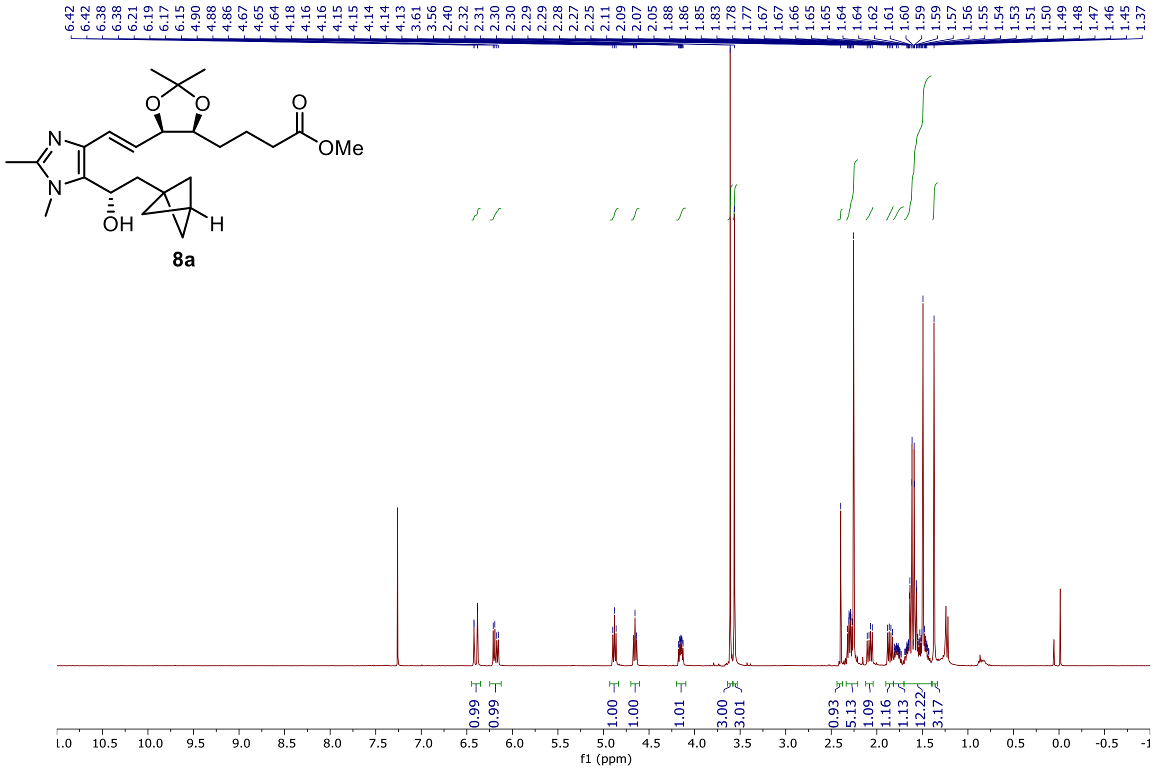

^{13}C-HMR (101 MHz, $CDCl_3$):

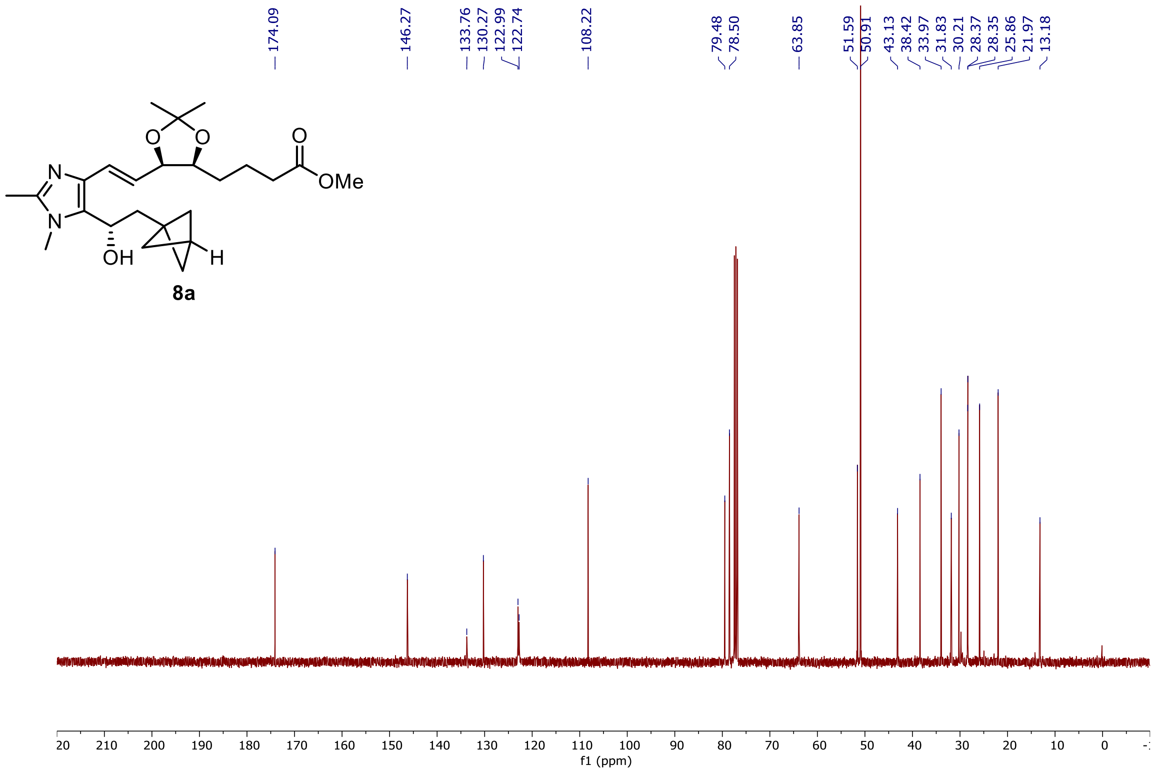

methyl 4-((4*S*,5*R*)-5-((E)-2-(5-((*R*)-2-(bicyclo[1.1.1]pentan-1-yl)-1-hydroxyethyl)-1,2-dimethyl-1H-imidazol-4-yl)vinyl)-2,2-dimethyl-1,3-dioxolan-4-yl)butanoate (8b)

[1]**H-NMR** (400 MHz, $CDCl_3$):

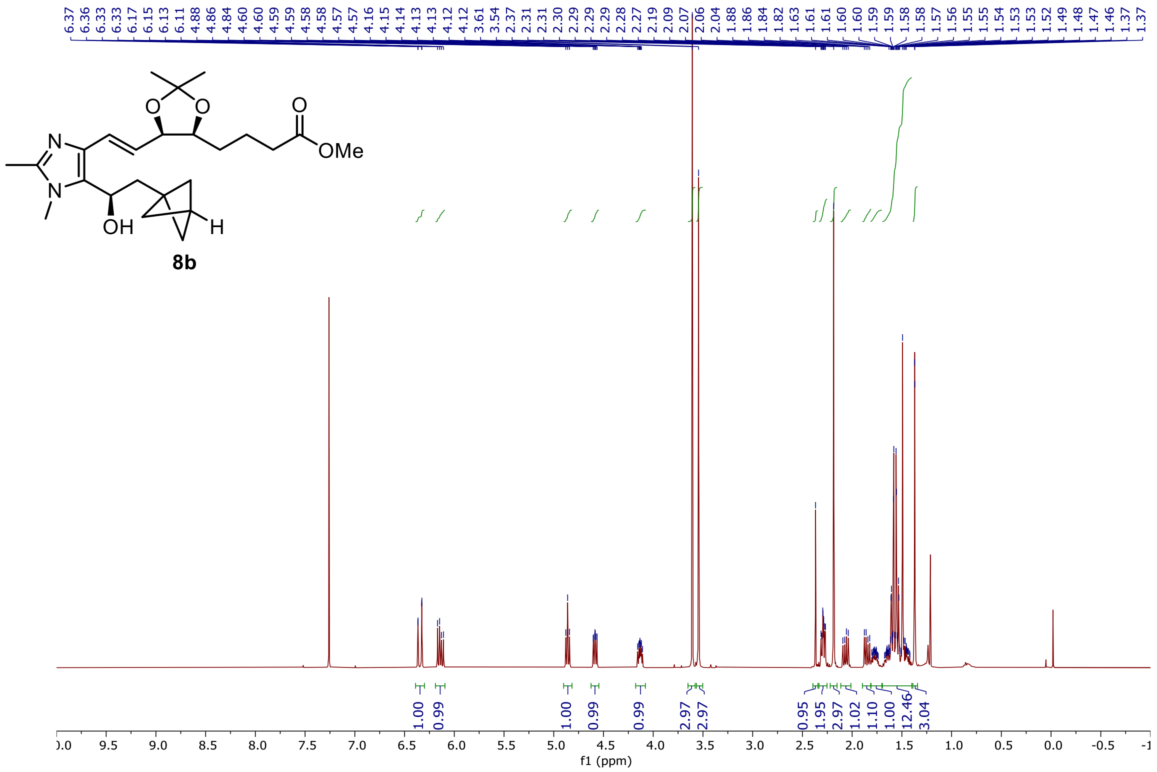

[13]**C-NMR** (101 MHz, $CDCl_3$):

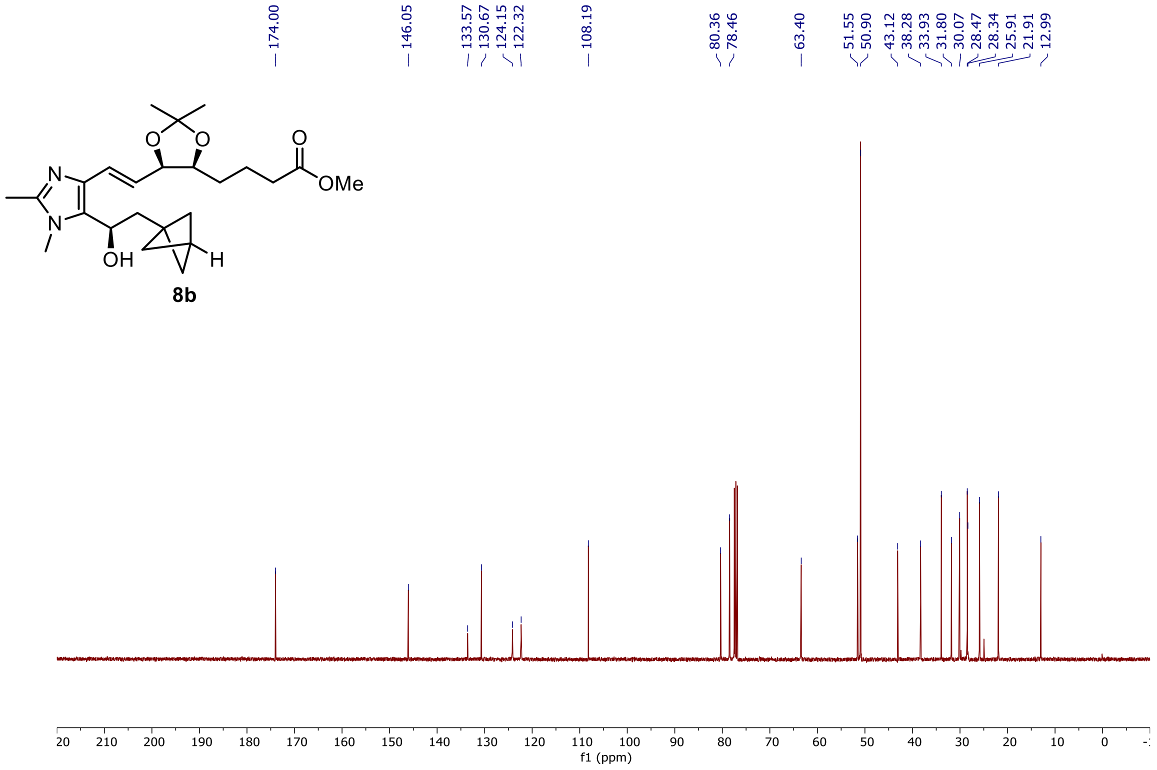

methyl (5*S*,6*R*,*E*)-8-(5-((*S*)-2-(bicyclo[1.1.1]pentan-1-yl)-1-hydroxyethyl)-1,2-dimethyl-1H-imidazol-4-yl)-5,6-dihydroxyoct-7-enoate (6a)

^{1}H-NMR (500 MHz, $CDCl_3$)**:**

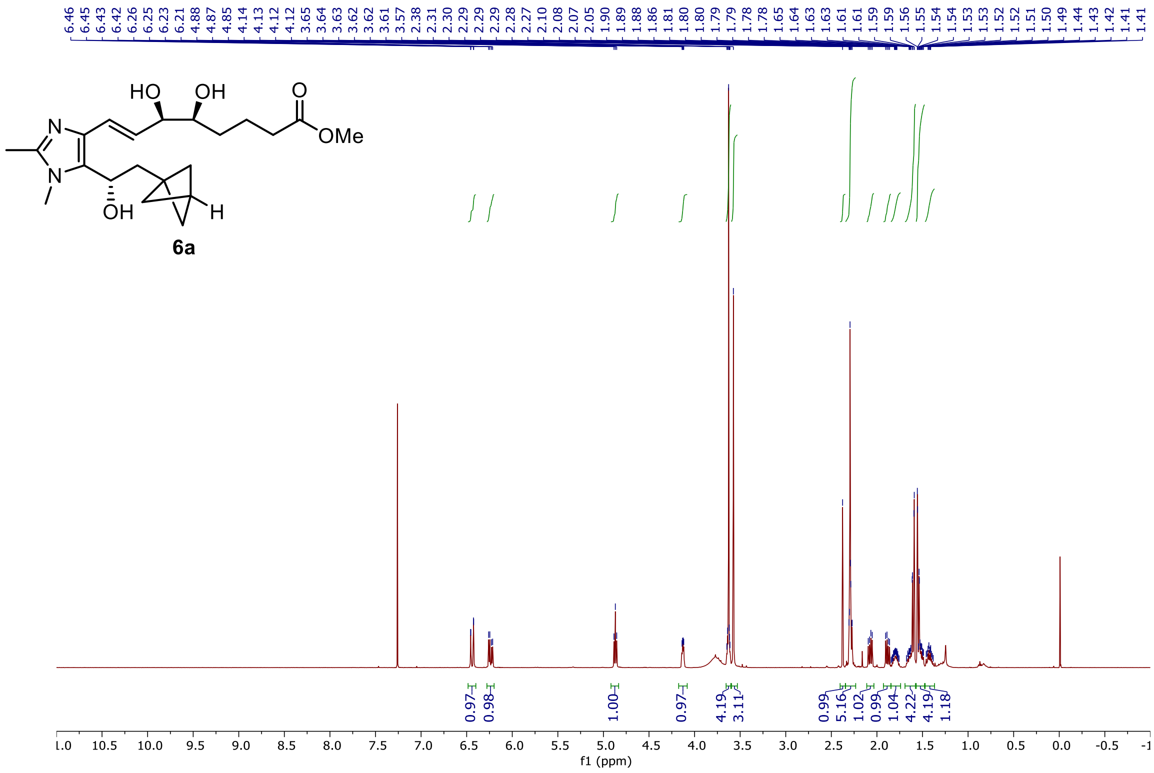

^{13}C-NMR (126 MHz, $CDCl_3$)**:**

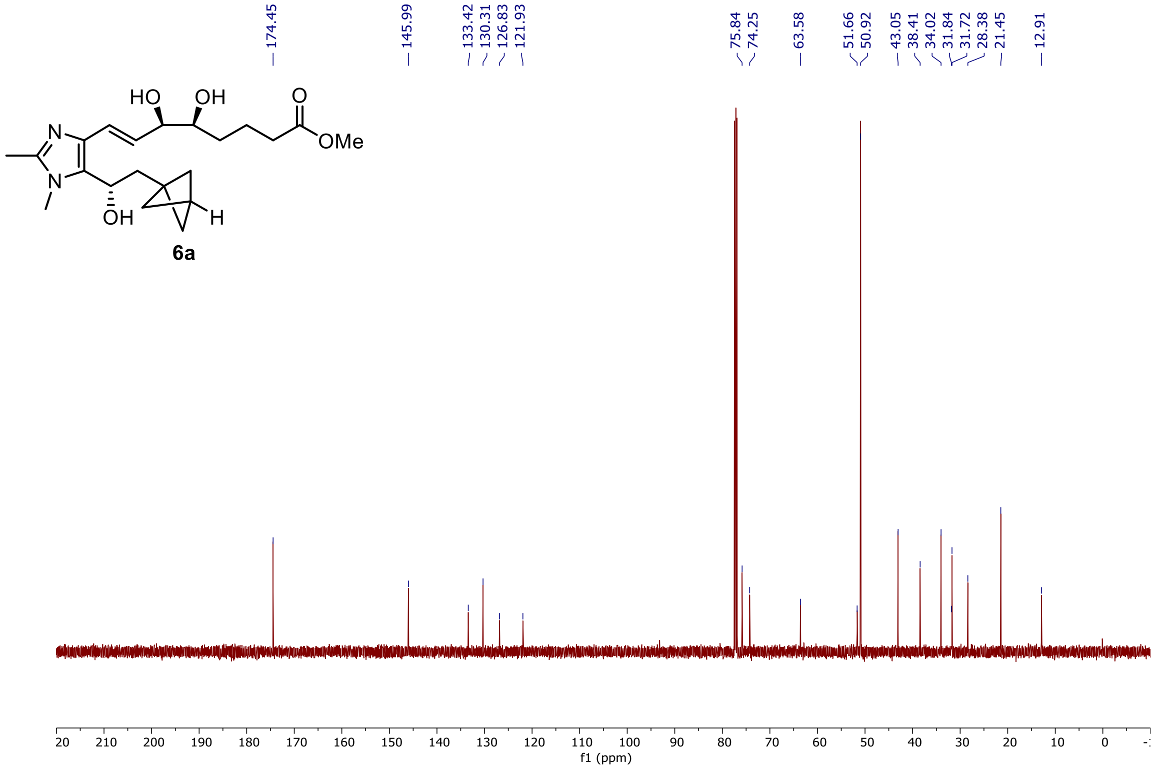

COSY:

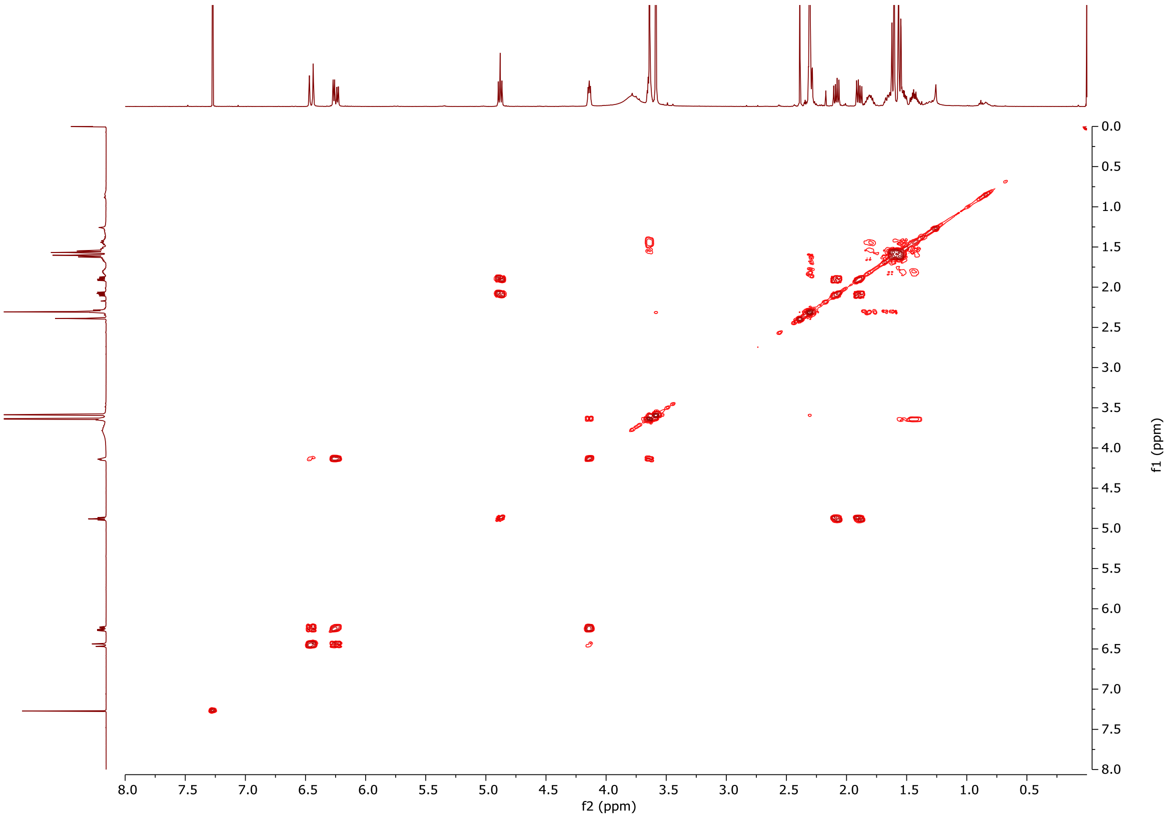

HSQC:

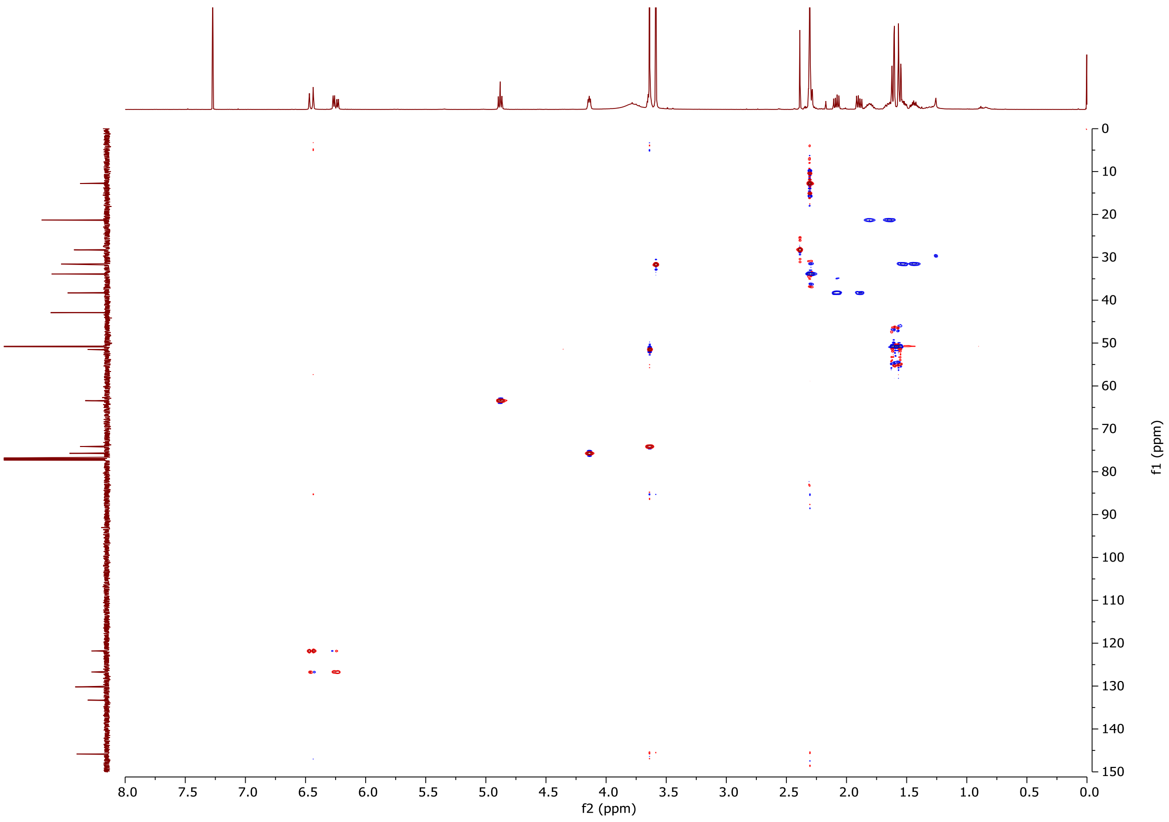

methyl (5*S*,6*R*,*E*)-8-(5-((*R*)-2-(bicyclo[1.1.1]pentan-1-yl)-1-hydroxyethyl)-1,2-dimethyl-1H-imidazol-4-yl)-5,6-dihydroxyoct-7-enoate (6b)

^{1}H-NMR (500 MHz, $CDCl_3$)**:**

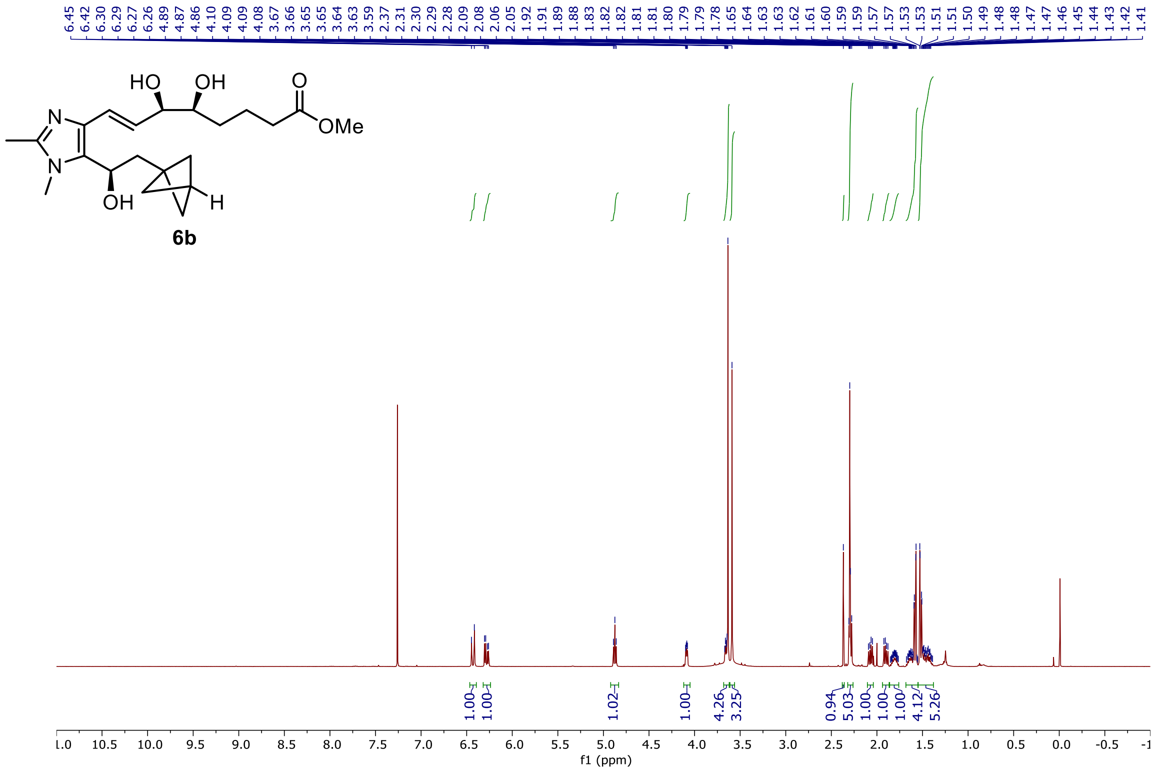

^{13}C-NMR (126 MHz, $CDCl_3$)**:**

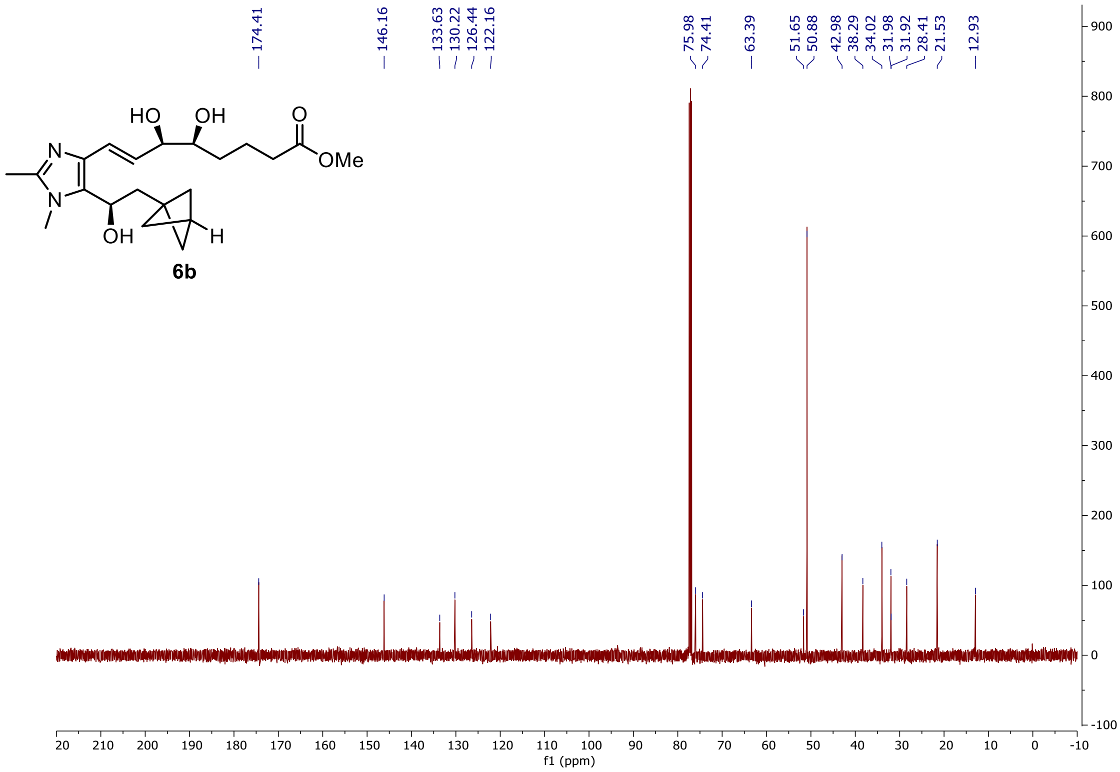

COSY:

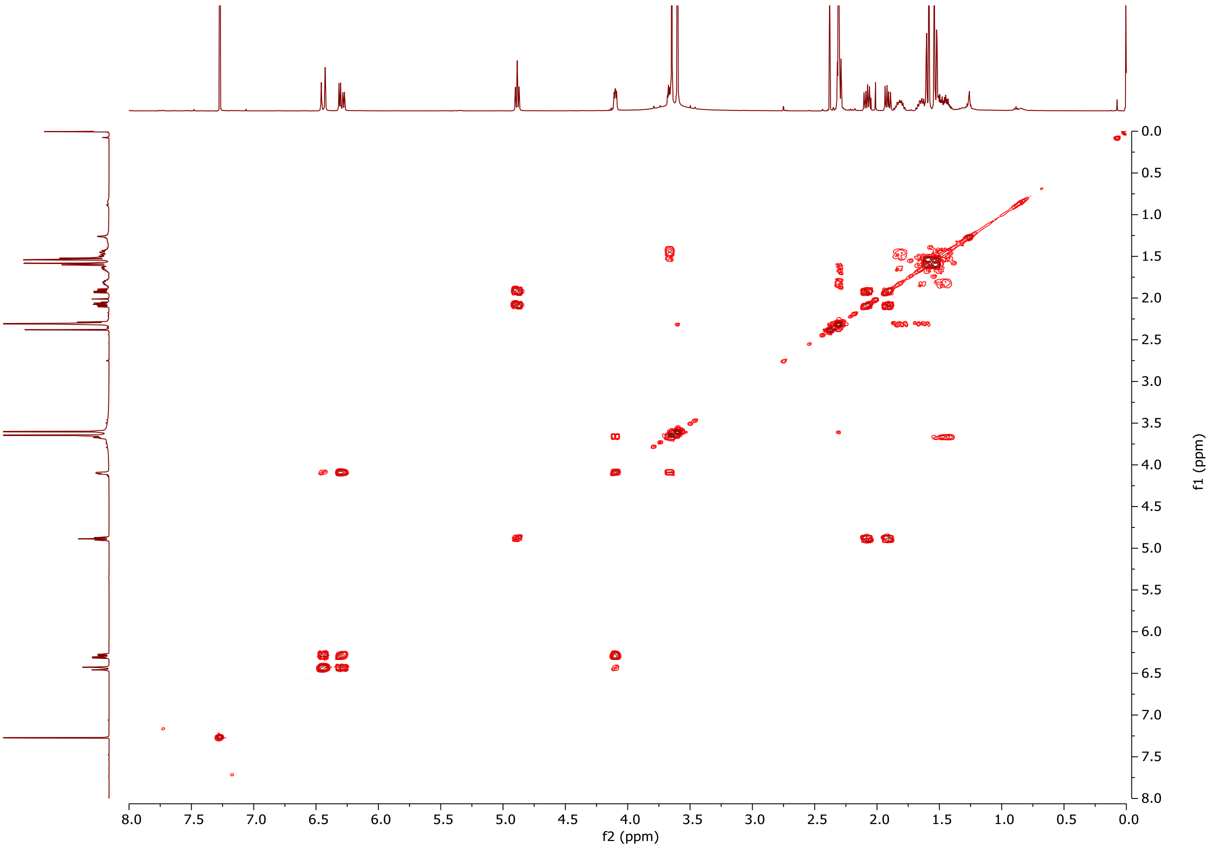

8.0
7.5
7.0
6.5
6.0
5.5
5.0
4.5
4.0
3.5
3.0
2.5
2.0
1.5
1.0
0.5
0.0
f2 (ppm)
f1 (ppm)

HSQC:

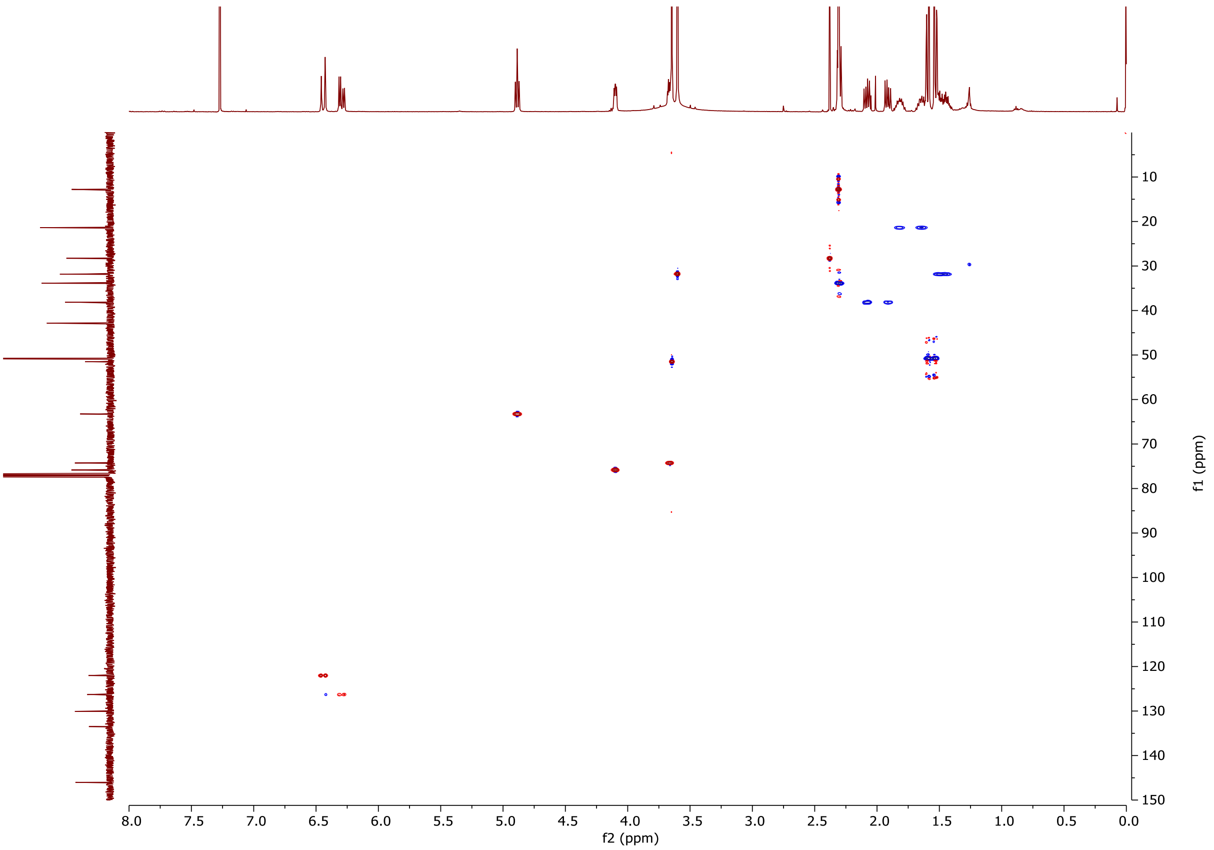

8.0
7.5
7.0
6.5
6.0
5.5
5.0
4.5
4.0
3.5
3.0
2.5
2.0
1.5
1.0
0.5
0.0
f2 (ppm)
10
20
30
40
50
60
70
80
90
100
110
120
130
140
150
f1 (ppm)
